# Design and Evaluation of Novel HIV-1 Protease Inhibitors Containing Phenols or Polyphenols as P2 Ligands with High Activity against DRV-Resistant HIV-1 Variants

**DOI:** 10.3390/ijms232214178

**Published:** 2022-11-16

**Authors:** Ling Ma, Jiajia Wen, Biao Dong, Jinming Zhou, Shangjiu Hu, Juxian Wang, Yucheng Wang, Mei Zhu, Shan Cen

**Affiliations:** 1 Institute of Medicinal Biotechnology, Chinese Academy of Medical Science and Peking Union Medical College, Beijing 100050, China; 2 Key Laboratory of the Ministry of Education for Advanced Catalysis Materials, Department of Chemistry, Zhejiang Normal University, Jinhua 321004, China

**Keywords:** HIV-1 protease inhibitors, phenol, polyphenol, enzymatic inhibitory activity, antiviral activity, darunavir-resistant HIV-1 variant

## Abstract

With the increasing prevalence of drug-resistant variants, novel potent HIV-1 protease inhibitors with broad-spectrum antiviral activity against multidrug-resistant causative viruses are urgently needed. Herein, we designed and synthesized a new series of HIV-1 protease inhibitors with phenols or polyphenols as the P2 ligands and a variety of sulfonamide analogs as the P2′ ligands. A number of these new inhibitors showed superb enzymatic inhibitory activity and antiviral activity. In particular, inhibitors **15d** and **15f** exhibited potent enzymatic inhibitory activity in the low picomolar range, and the latter showed excellent activity against the Darunavir-resistant HIV-1 variant. Furthermore, the molecular modeling studies provided insight into the ligand-binding site interactions between inhibitors and the enzyme cavity, and they sparked inspiration for the further optimization of potent inhibitors.

## 1. Introduction

Acquired immune deficiency syndrome (AIDS) is a widespread disease caused by human immunodeficiency virus (HIV), which has seriously threatened human health since the first case was detected in 1981 [1]. Its main type, HIV-1, was internationally recognized as a level one carcinogen in 2017 [2]. Fortunately, the development of anti-HIV drugs and highly active antiretroviral therapy (HAART) for HIV/AIDS over the past decades has reduced the mortality and morbidity rates dramatically [3,4,5,6,7]. HIV-1 protease inhibitors (PIs) play a critical role in inhibiting viral maturation [8]. Ten HIV-1 PIs that substantively improve the quality of life and provide more flexible treatment options have been applied in clinical settings. In spite of such progress, the continuous emergence of drug-resistant variants reduces therapeutical options. It is noteworthy that the newest second-generation synthetic peptidomimetic PI Darunavir (DRV) is clinically relatively impotent against highly DRV-resistant HIV-1 variants [9,10,11,12,13]. Thus, the development of new potent HIV-1 PIs with broad-spectrum antiviral activity against multidrug-resistant virus variants has attracted much attention.

**Figure 1:**
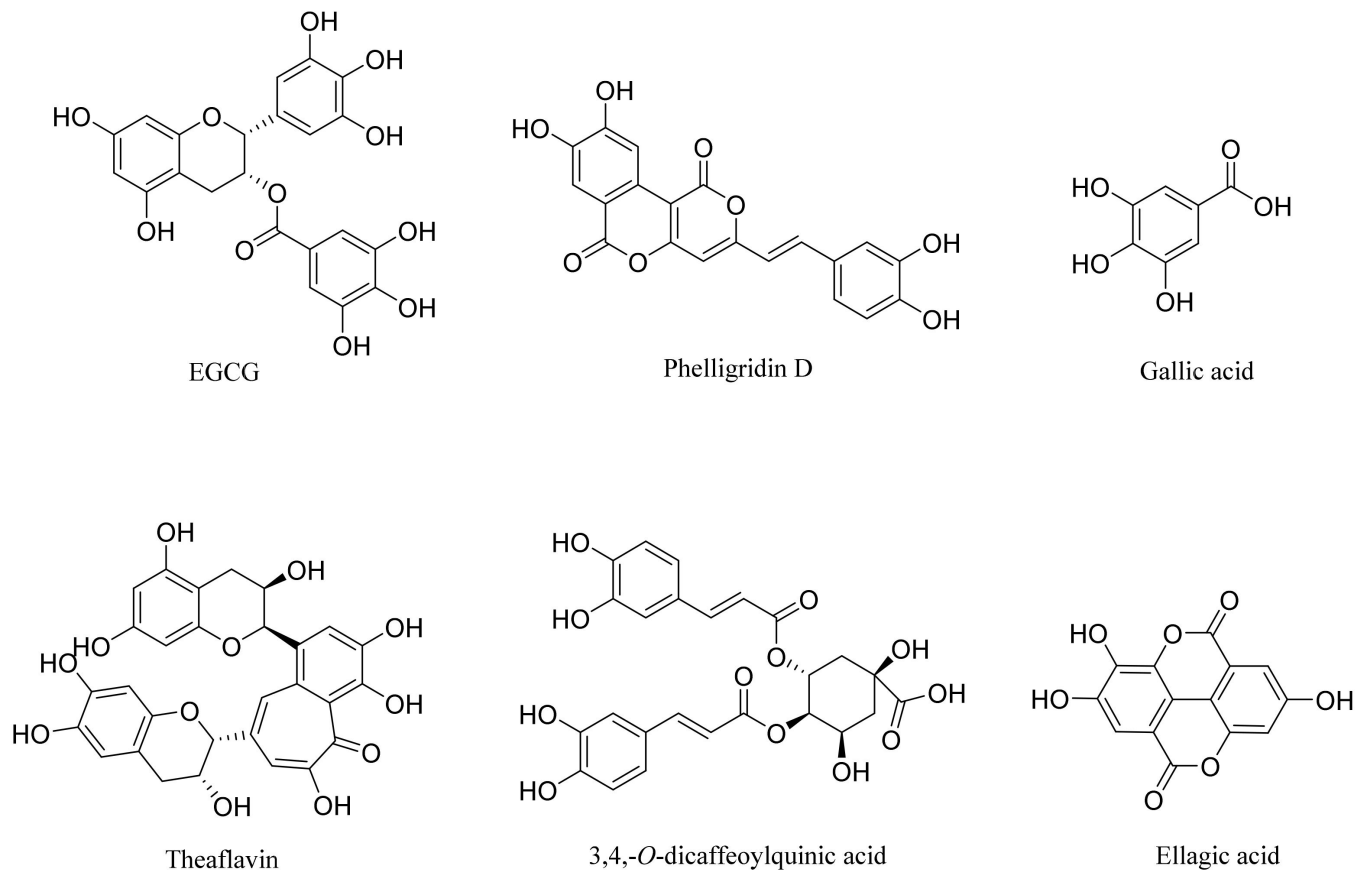
Representative chemical structures of phenols or polyphenols with antiviral activity.

HIV protease is a homodimeric aspartyl protease with C2 symmetric in the free form [14], containing 99 amino acids in both of its chains A and B. The active ligand-binding site organizes different regions of the enzyme. The active site of the protein is formed by the dimerization of the two monomers and is crowned by two identical flexible glycine rich flaps. As a member of the aspartic protease family, the protease contains a catalytic triad (Asp-Thr-Gly) in both the chains keeping functional aspartate residues at the dimer interface. Research has demonstrated that the binding characteristics between a protease inhibitor and the active site of HIV-1 protease are key factors in the development of resistance [15]. This need sparked the rational design (also referred to as structure-based design) of novel HIV-1 PIs [16]. One important strategy used to design drugs in order to combat drug resistance is to maximize the protease active site interactions and to promote extensive hydrogen bonds between the protease active site backbone atoms and the inhibitor [17,18,19,20,21]. Hence, we introduced phenols or polyphenols as the P2 ligands, which might promote hydrogen bonds with the amino groups of residues in the corresponding S2 subsite [22,23,24,25]. Moreover, phenols or polyphenols are important bioactive substances with broad-spectrum activity against an extensive range of viruses [26]. For instance, (−)-epigallocatechin-3-gallate (EGCG, Figure 1) can inhibit hepatitis C virus (HCV) entry by acting on the viral particle, and it is active against HIV-1 by inhibiting the replication of both reverse transcriptase and p24 [27,28]. Phelligridin D exhibits excellent activity against influenza virus strains H1N1, H5N1, and H3N2, with IC_50_ values of 8.8, 10.9, and 10.3 μM, respectively [29]. Furthermore, gallic acid (GA) and gallates show activity against herpes simplex virus type 1 (HSV-1) and can inhibit HIV-1 to some degree [30]. Theaflavins (TFs) from black tea have been confirmed to show activity against calicivirus, HSV-1, influenza A, HCV, and HIV-1 [31,32,33,34,35]. In addition, 3,4-*O*-dicaffeoylquinic acid suppresses HBsAg and HBeAg production and markedly decreases hepatitis B virus (HBV) covalently closed circular DNA content [36]. Moreover, ellagic acid might be potent against HBV by blocking HBeAg secretion [37].

**Figure 2:**
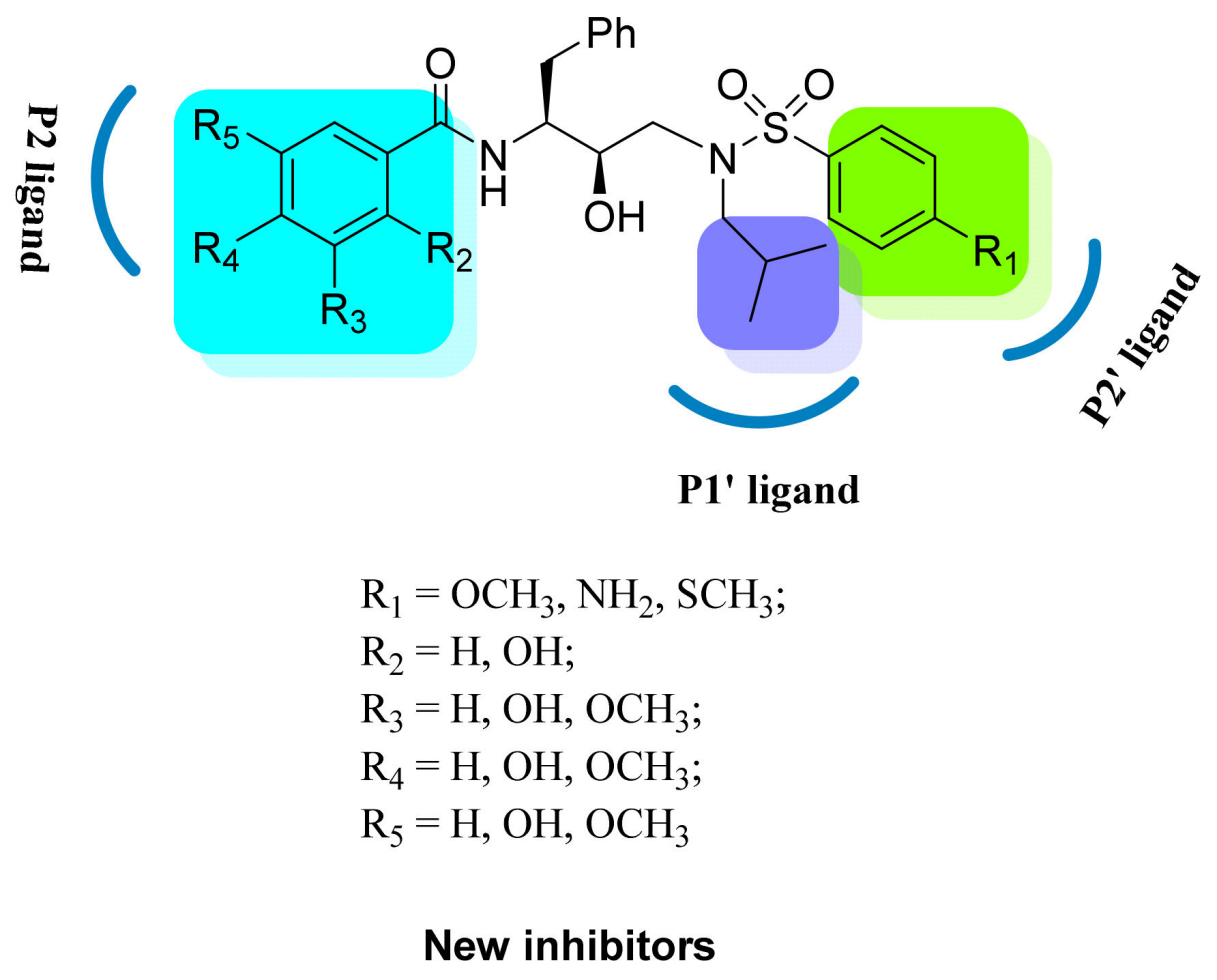
Structures of newly designed HIV-1 protease inhibitors. The canonical nomenclature for the inhibitor moiety position is indicated using DRV.

Considering the abovementioned observations, phenols or polyphenols might be conducive to enhancing anti-HIV-1 activity via introduction into the P2 ligand of PIs. More importantly, the strong hydrogen bonds formed by phenolic hydroxyl with the backbone amide of residues in the protease might be responsible for antiviral drug resistance. Herein, we designed and synthesized a series of HIV-1 PIs with phenols in the P2 ligands, isobutyl in the P1’ ligand, and electron-donating groups in the P2′ ligands as shown in Figure 2.

## 2. Results

### 2.1. Chemistry

**Scheme 1:**
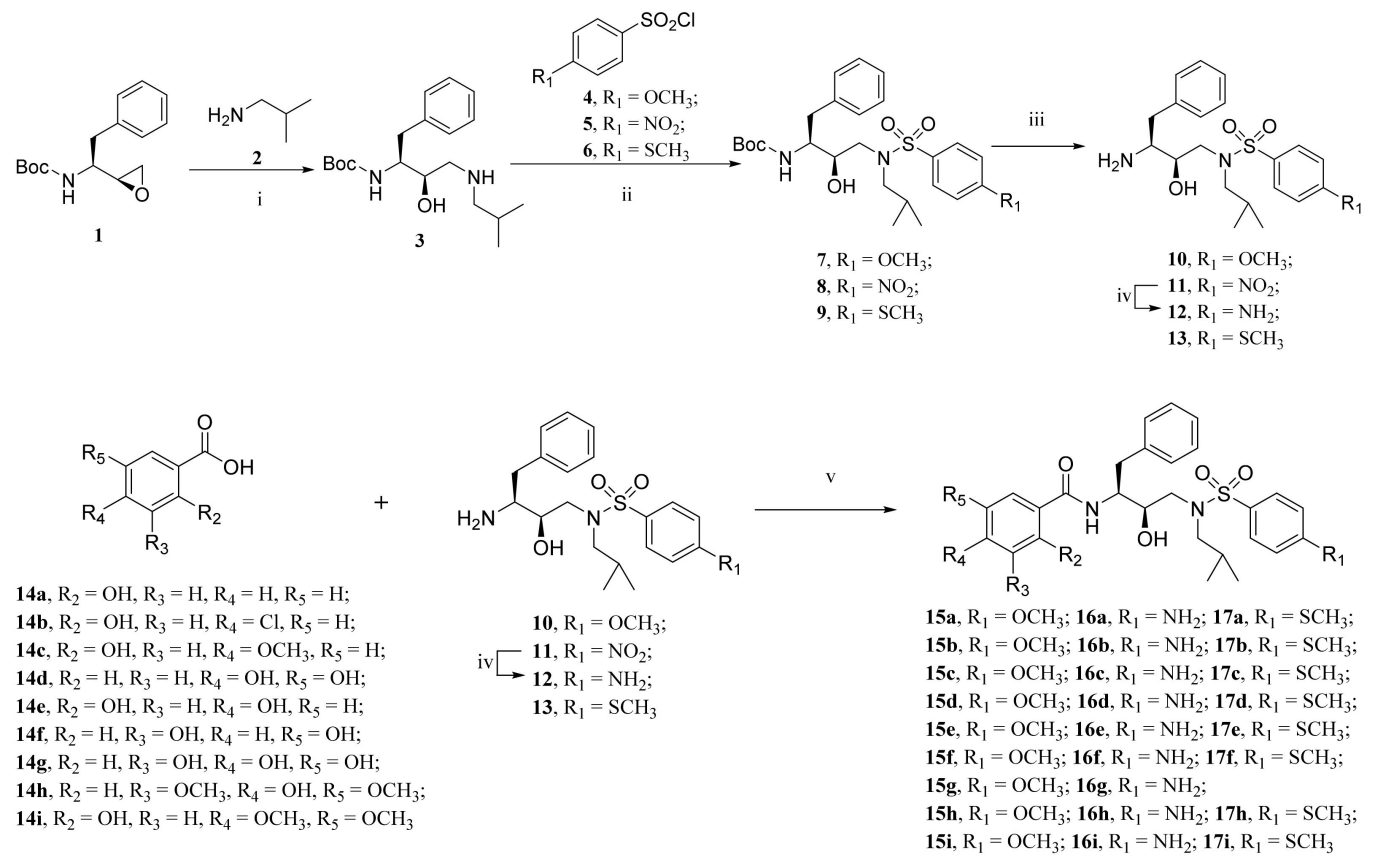
Synthesis process of compounds **15a–15i, 16a–16i, 17a–17f, and 17g–17i**. Reagents and conditions: (**i**) i-BuNH_2_, CH_3_CN, 80 °C, 6 h; (**ii**) aryl sulfonyl chloride, DIEA, DMAP, THF, 0 °C to room temperature, 3–5 h; (**iii**) CH_2_Cl_2_-CF_3_COOH (1:1), 0 °C to room temperature, 3 h; (**iv**) H_2_ (gas), 50 psi, 10% Pd/C, CH_3_OH, room temperature, 2 h; (**v**) EDCI, HOBt, DMAP, anhydrous DMF, argon, 0 °C to room temperature, 3 h.

Outlined in Scheme 1 is the synthesis process of the target inhibitors **15a**–**17i**. Amino alcohol **3** was synthesized from the commercially available materials 1 and 2 according to the literature [38]. The treatment of **3** with p-substituted benzenesulfonyl chlorides (**4**–**6**) under the catalysis of DIEA and DMAP provided sulfonamide derivatives (**7**–**9**) in good yields (82–91%), and this treatment was followed by exposure to trifluoroacetic acid at 0–25 °C for 3 h to remove the Boc group, affording the corresponding amines in yields of 78–83% [39,40]. The catalytic hydrogenation of **11** over 10% Pd-C in methanol affected the reduction of the nitro group to diamine **12** in a 94% yield [41]. The reaction of the amines with phenolic acids **14a**–**i** in anhydrous DMF in the presence of EDCI/HOBt/DMAP at 0–25 °C for 2–3 h provided the corresponding target compounds **15a**–**17i** in yields of 68–88%. Experimental details for the synthesis process and the spectroscopic characterization of the compounds can be found in the Supplementary Material.

### 2.2. HIV-1 Enzymatic Inhibitory Activity Assay

**Figure 3:**
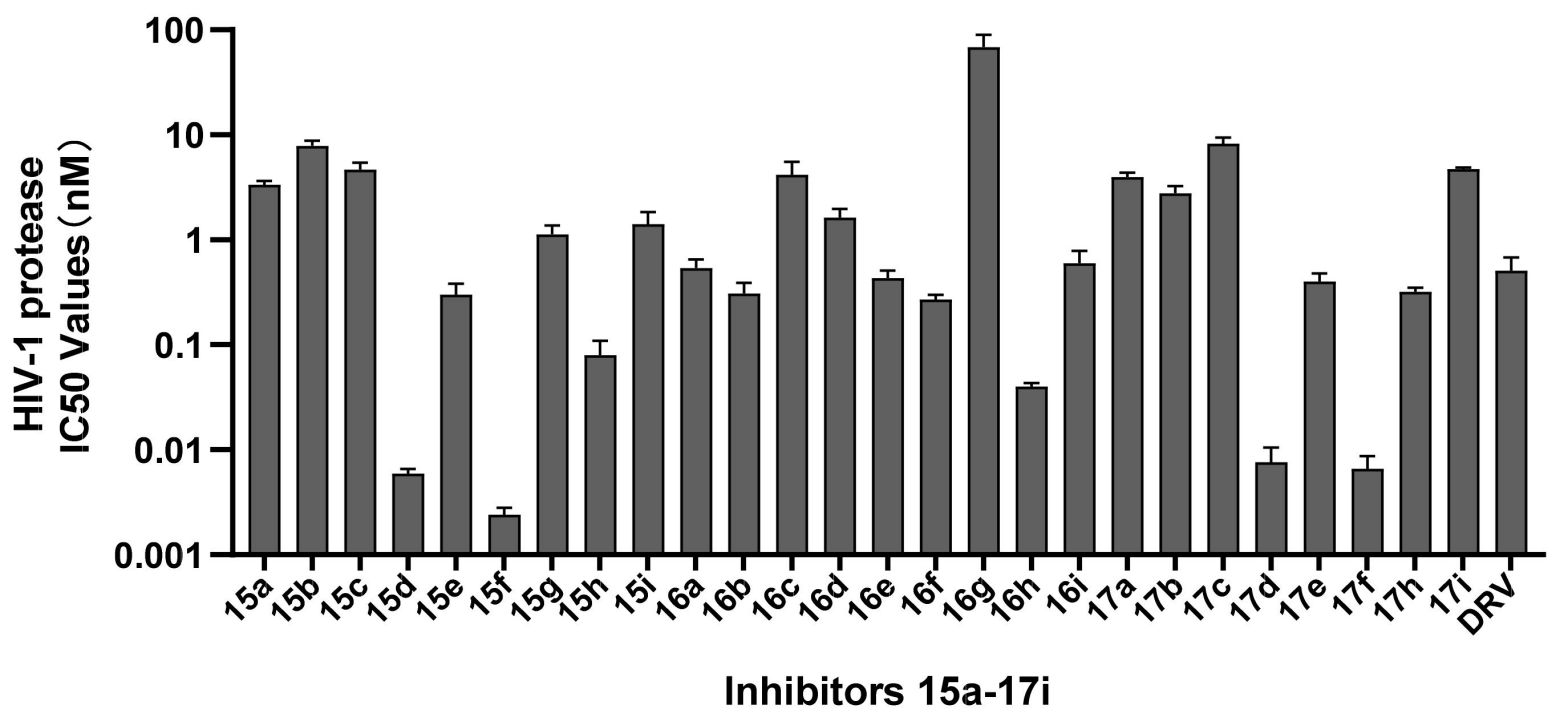
Enzymatic inhibitory activity of inhibitors. All assays were conducted in triplicate, and the data shown represent mean values (±SD) derived from the results of three independent experiments.

The fluorescence resonance energy transfer (FRET) method was used to evaluate the inhibitory activity of the new class of HIV-1 PIs with DRV as a control [42]. The results are shown in Table 1. Phenols or polyphenol derivatives as the P2 ligands were investigated in combination with electron-donating substituted phenylsulfonamides as the P2′ ligands. As can be seen in Table 1, inhibitor **15f** with 3,5-dihydroxybenzoyl as the P2 ligand and 4-methoxyphenylsulfonamide as the P2′ ligand exhibited an over 200-fold enhancement of enzymatic inhibitory activity, with an IC_50_ value of 2.4 pM, compared with DRV [43,44]. Furthermore, the inhibitors **15d**, **17d**, and **17f** also showed very potent activity at low picomolar values of 5.9–7.6 pM, which indicates the importance of a hydroxyl group in the proper position. To explain this in detail, the 3,4-disubstitution or 3,5-disubstitution of phenolic hydroxyl groups in the P2 ligand favored the inhibitory activity of compounds, which could form hydrogen bonding interactions or other van der Waals interactions between the compounds and the enzyme cavity, which can be deduced from the molecular modeling studies below. In addition, almost all the derivatives displayed inhibitory activity with IC_50_ values in a low nanomolar or picomolar range, except for **16g**, which had an IC_50_ value of 68 nM (Figure 3).

As it turned out, inhibitors with 4-methoxy or 4-methylthio phenylsulfonamide groups as the P2′ ligands exhibited generally improved antiviral activity compared with those with a 4-aminophenylsulfonamide P2′ ligand and those containing dihydroxy- or trihydroxy-benzoyl as the P2 ligands, such as **15f** and **17f** vs. **16f**, **15d** and **17d** vs. **16d**, **15e** and **17e** vs. **16e**, and **15g** vs. **16g**. Contrary to the results presented above, a substantial reduction in antiviral activity was observed among the inhibitors with 4-methoxy or 4-methylthio phenylsulfonamide groups compared with those with 4-aminophenylsulfonamide as the P2′ ligands and with monohydroxy, methoxyl, or chlorine substituent groups in the P2 ligands, for instance, **15a** and **17a** vs. **16a**, **15b** and **17b** vs. **16b**, **15c** and **17c** vs. **16c**, **15h**, and **17h** vs. **16h**, and **15i** and **17i** vs. **16i**.

Furthermore, the biological activity decreased significantly when the hydroxyl group was replaced by chlorine or a methoxyl group in the disubstituted phenyl in the P2 ligand, such as **15e** vs. **15b** and **15c**. The main reason for this was that the 3-hydroxyl group of phenolic acid lost the opportunity to form van der Waals interactions with the active site backbone atoms of the protease, which was also verified in the molecular modeling of inhibitor **15f** in Figure 5 [45]. Additionally, the incorporation of symmetric substituents as the P2 ligands improved the activity; compare, for example, **15f** vs. **15d**, **15h** vs. **15i**, **16f** vs. **16d**, **16h** vs. **16i**, **17f** vs. **17d**, and **17h** vs. **17i**.

However, inhibitors with trihydroxyphenyl in the P2 ligand showed an obvious loss of enzyme inhibitory activity compared with those with dihydroxyphenyl in the P2 ligand, for example, **15g** vs. **15d** and **15f** or **16g** vs. **16d** and **16f**. More hydroxyl groups might negatively affect the efficacy of forming additional hydrogen bonds in the S2 subsite due to intramolecular hydrogen bonds, steric bulk, or increased hydrophilicity.

### 2.3. HIV-1 Infectivity Assay

**Figure 4:**
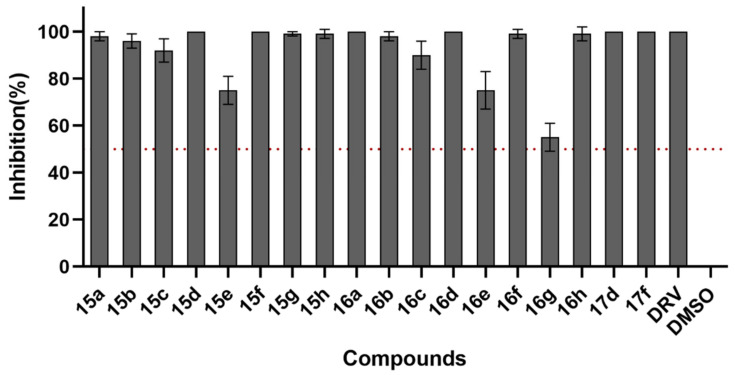
Effects of inhibitors on late-stage HIV-1. All assays were conducted in triplicate, and the data shown represent mean values (±SD) derived from the results of three independent experiments.

In preliminary studies, we evaluated the effectivity of selected inhibitors in a single-round infection assay using HIV-1 pseudotyped with vesicular stomatitis virus G protein (VSVg), in which virus-producing cells were treated as described previously [39,46]. Surprisingly, **15d**, **15f**, **16a**, **16d**, **17d**, and **17f** were equipotent with DRV. Moreover, the majority of inhibitors showed significant antiviral activity, with inhibition above 90%, except for **15e**, **16e**, and **16g**, as shown in Table 2 and Figure 4, which is in agreement with their excellent activity against HIV-1 protease in vitro.

### 2.4. Molecular Modeling Studies

**Figure 5:**
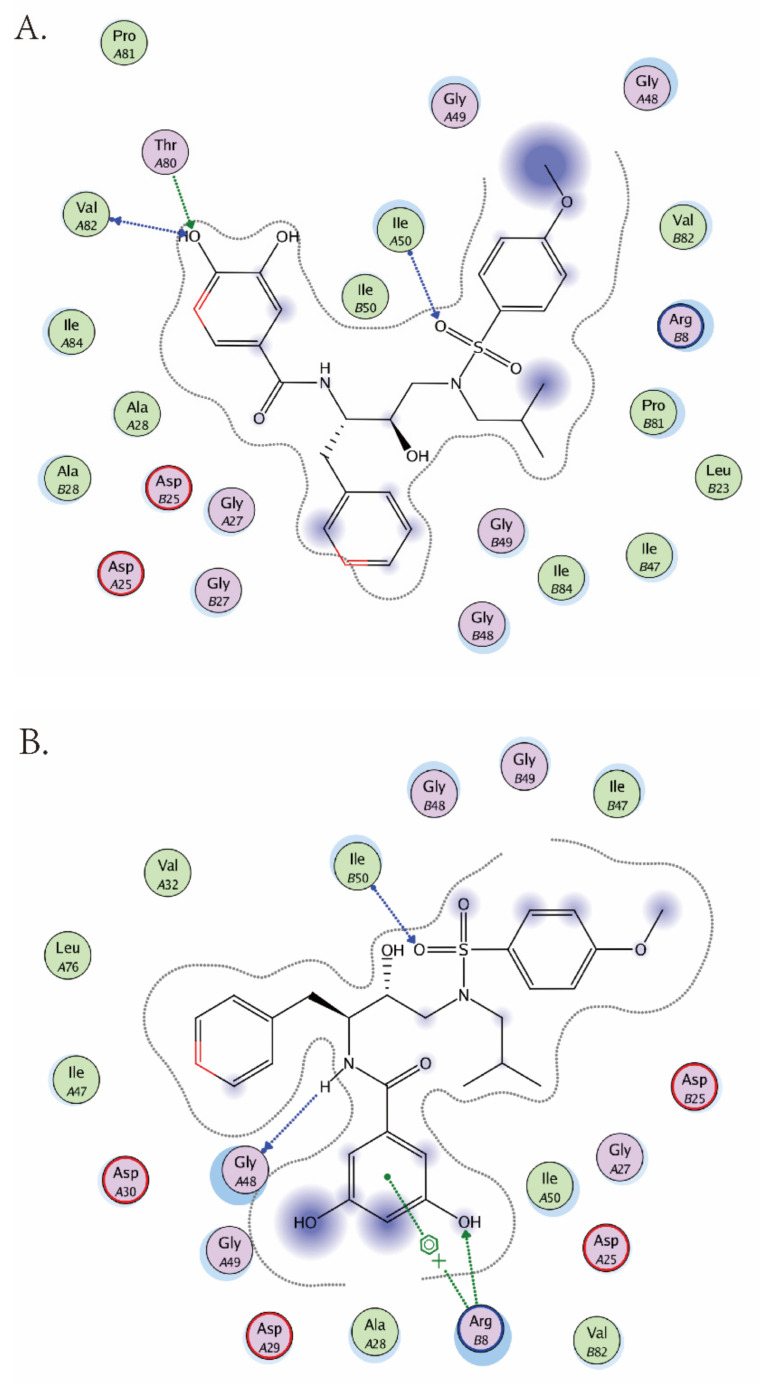
Molecular modeling of inhibitors **15d** and **15f**. (**A**) Ligplot interaction of **15d**. (**B**) Ligplot interaction of **15f**. Ligand exposure is represented as purple spheres, hydrogen bonding is depicted as blue or green arrows, and π–π interactions are depicted as green lines.

These inhibitors with phenols or polyphenols as the P2 ligands were specifically designed to promote extensive hydrogen bond formation or van der Waals interactions with the HIV-1 protease active site backbone atoms. Molecular modeling studies were conducted using the Molecular Operating Environment (MOE) (version 2009.06, Chemical Computing Group Inc., Montreal, QC, Canada) to verify the concepts and to provide insight into their ligand-binding site interactions. Inhibitors **15d** and **15f**, two molecules exhibiting the most activity in vitro and in cells among all the tested compounds, were selected for molecular modeling (Figure 5) [47]. The protease structure (PDB-ID: 4mc9) was taken from the Protein Data Bank [48].

The new ligand fit well into the S2 site of the protease and showed good van der Waals interactions with some key amino acid residues. As can be seen, strong hydrogen bonds formed between the hydroxyl in the P2 ligand of inhibitor **15d** and amino acid residues Val82 and Thr80 in the S2 subsite. Furthermore, one of the sulfonamide oxygens formed hydrogen bonds with the backbone NH group of Ile50 located in the flaps [49]. In addition, the methoxy group in the P2′ ligand showed polar interactions with the S2′ subsite of the protease (Figure 5A).

The P2 polyphenol ligand in inhibitor **15f** showed enhanced van der Waals interactions in the S2 site compared with **15d** (Figure 5B). One of the hydroxyls in the P2 ligand formed strong hydrogen bonds with the amino acid residue Arg8′, and the other hydroxyl showed polar interactions with the outer atoms of the S2 subsite. In particular, it showed a π–π interaction between the benzene ring in the P2 ligand and Arg8′ in the S2 subsite. Furthermore, the hydrogen atom in the amide group formed hydrogen bonds with the chain atom of Gly48. In addition, one of the sulfonamide oxygens formed hydrogen bonds with the backbone NH group of Ile50’, and the second oxygen atom showed favorable van der Waals interactions in the flaps. The methoxy group in the P2′ ligand also formed hydrophobic contacts with the amino acid residues in the S2′ subsite. The network of extensive interactions with the HIV-1 protease backbone in inhibitor **15f** is of crucial importance for its ability to combat drug resistance.

### 2.5. Correlation of Phenol or Polyphenol Analogs

**Figure 6:**
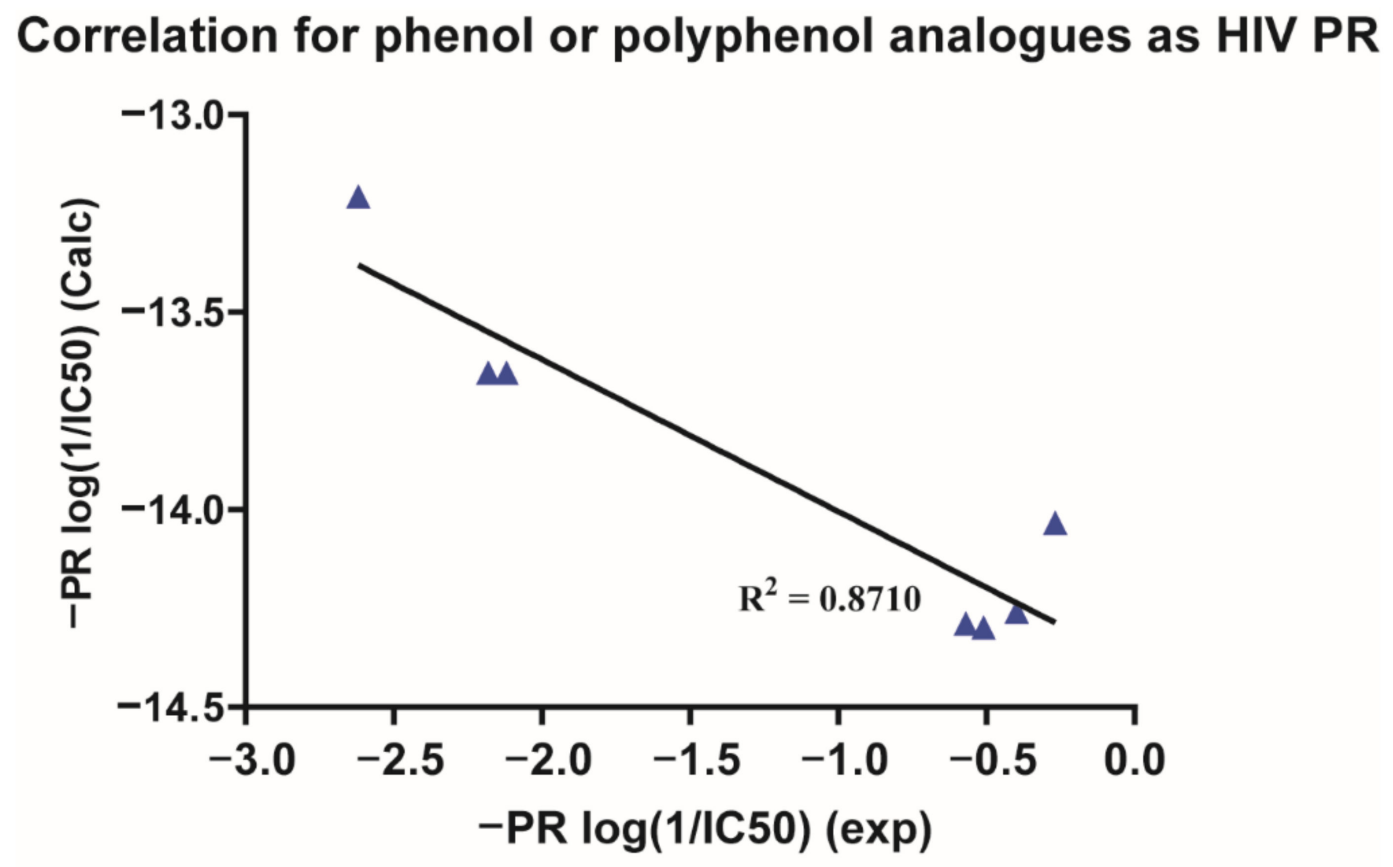
Strong correlation of docked phenol or polyphenol analogs supports a common mode of binding for HIV protease.

As shown in Figure 6, further validation was carried out by analyzing the structure–activity relationship (SAR) of docked inhibitors **15f**, **16a**, **16b**, **16f**, **17d**, **17e**, and **17f**. The correlation observed between these two sets of IC_50_ data (expected vs. calculated, coefficient of correlation = 0.87) supported the molecular modeling with a common mode of binding as a valid platform for HIV-1 PI design.

### 2.6. Binding Assay

**Figure 7:**
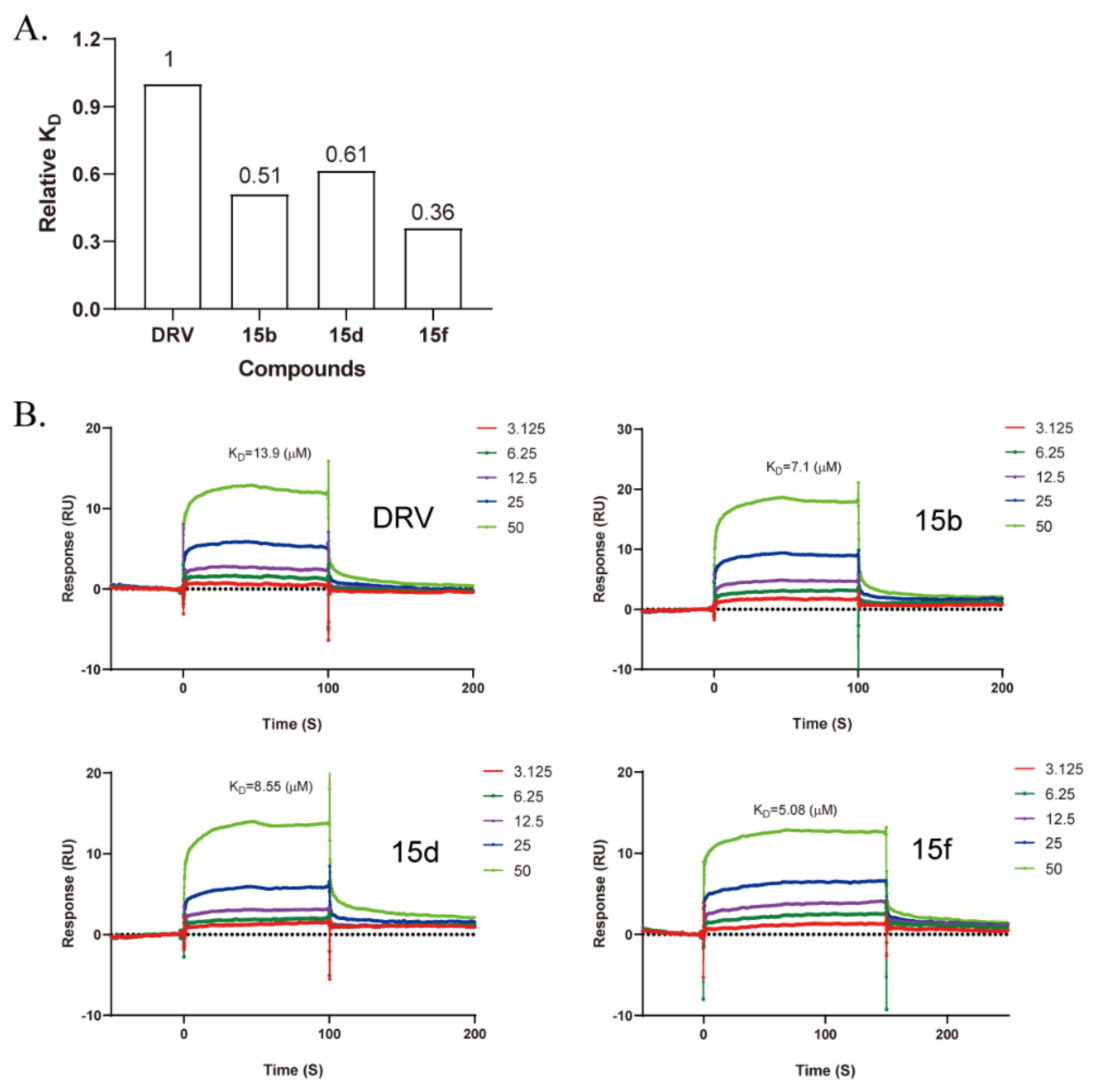
Kinetic and equilibrium binding analysis of selected inhibitors binding to the protease. Purified HIV-1 protease was immobilized on a CM5 sensor chip using a Biacore T200 instrument. The association and dissociation curves of these compounds are shown. Compounds were dissolved with a series of increasing concentrations, and KD values were acquired from fitting to a 1:1 binding model using Biacore T200 evaluation software 2.0. KD, equilibrium dissociation constant. All assays were conducted in triplicate, and the data shown represent one of three independent experiments.

To find inhibitors with better affinity and to validate the SAR, we next measured the binding affinity of the inhibitors with HIV-1 protease using the SPR assay in vitro. Since the inhibitors 15b, 15d, and 15f exhibit better activity than the other tested molecules, they were selected for the SPR studies. First, the HIV-1 protease was immobilized on a CM5 chip. Then, compounds flowed across the surface. We found that inhibitors **15b**, **15d**, and **15f** bound to the protease efficiently, with equilibrium dissociation constant (KD) values ranging from 5.08 to 13.9 μM in our binding system (Figure 7B). As shown in Figure 7A, we compared the KDs of **15b**, **15d**, and **15f** binding to the protease with that of DRV, and we found that the inhibitors exhibited stronger binding affinity.

### 2.7. Antiviral Activity against the DRV-Resistant HIV-1 Variant

**Figure 8:**
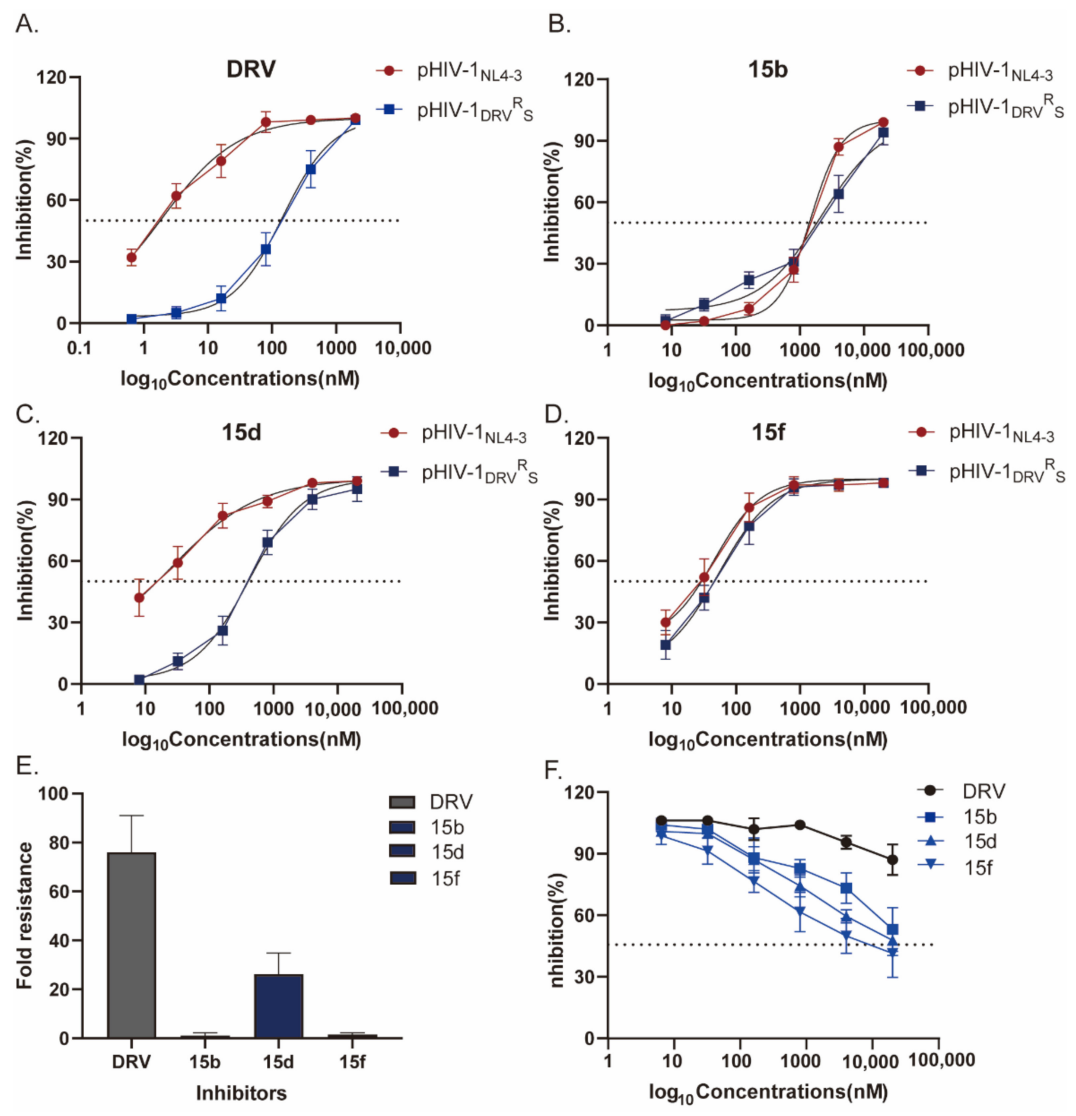
Antiviral activity of **15b**, **15d**, and **15f** against multidrug-resistant HIV-1 variants. (**A**–**D**) Dose–response relationship of compounds DRV, **15b**, **15d**, and **15f** against WT HIV-1 and DRV-resistant mutant. (**E**) Fold resistance is defined as EC_50_ (mutant)/EC_50_ (WT). (**F**) 293T cells were treated with these inhibitors as indicated. The CC_50_ values were measured with the cell counting kit-8. All assays were conducted in triplicate, and the results shown are the average of three independent experiments; error bars indicate SD.

In view of the efforts to develop potent PIs with a high genetic barrier against multi-PI-resistant HIV-1 variants, especially against the DRV-resistant HIV-1 variant, we tested the inhibitors **15b**, **15d**, and **15f** for activity against DRV-sensitive or -resistant pseudotyped HIV-1 via a single-round infection assay. Four amino acid substitutions (V32I, L33F, I54M, and I84V), which conferred high resistance to DRV, were introduced into pNL4-3-E^-^R^-^ (pHIV-1_NL4-3_), resulting in DRV-resistant HIV-1 proviral DNA pHIV-1_DRV_^R^_S_ [11]. As shown in Table 3 and Figure 8, the activity of DRV against the highly DRV-resistant HIV-1 variants was strongly reduced, with a 76-fold increase in the EC_50_ value. By contrast, inhibitors **15b**, **15d**, and **15f** maintained potent activity against the DRV-resistant HIV-1 variant compared with the wild-type virus, with EC_50_ values increasing 1.3- to 26-fold. However, in comparison with the superb antiviral activity, the loss of the cellular potency of these inhibitors might be attributed to increased hydrophobicity according to the calculated partition coefficient (LogP) values being higher than those of the control DRV, which indicates that membrane transport was a key factor for cellular activity [50,51].

Furthermore, all three compounds (**15b**, **15d**, and **15f**) had relatively low toxicity to 293T cells, with CC_50_ > 20 μM (Table 1 and Figure 8F). The CC_50_ values of these compounds were much higher than their EC_50_ values (Table 2). These results indicate that the inhibition of HIV-1 and the pseudovirus variants was not due to the cytotoxicity of the tested inhibitors. The design of prodrugs with enhanced intracellular antiretroviral activity will be our next research focus.

## 3. Discussion

We designed a novel series of HIV-1 PIs with phenols or polyphenols in the P2 ligand to promote hydrogen bond formation with backbone atoms of the S2 subsite. A number of these inhibitors exhibited very potent activity against multidrug-resistant HIV-1 variants. Notably, inhibitors **15d** and **15f** containing dihydroxyl in the P2 ligand and 4-methoxyphenylsulfonamide as the P2′ ligand exhibited superb enzymatic inhibitory activity in the low picomolar range. Furthermore, inhibitor **15f** maintained excellent activity against DRV-resistant HIV-1 variants, with only a 1.5-fold increase in EC_50_ compared with that of the wild-type (WT) virus. It should be noted that the phenolic compounds tested in this work may alter FRET values due to the substrate-dependent quenching effect, falsely resulting in high potency. The reported FRET values herein were not calibrated with an inherent quenching control (Table 1), even though the possible quenching effect of primary hits was assessed later.

The molecular modeling studies revealed that the new P2 phenol/polyphenol ligand filled the pocket of the S2 subsite and formed significant van der Waals interactions with the residues. In particular, besides hydrogen bonds, the new approach of promoting π–π interactions in inhibitor **15f** with the backbone residues might be of importance for the superb activity and potency against highly resistant HIV-1 strains. This kind of P2 scaffold may serve as an excellent source of inspiration for the further optimization of potent HIV-1 PIs. Furthermore, prodrugs with enhanced cellular potency might be designed, which is our current research focus.

## 4. Materials and Methods

### 4.1. Cells, Viruses, Plasmids, and Reagents

HEK293T cells (ATCC, Manassas, VA, USA) were cultured in DMEM (GBICO, Billings, MT, USA) supplemented with 10% fetal bovine serum (FBS) (GBICO). SupT1 cells (ATCC) were maintained in RPMI-1640 (GBICO) containing 10% FBS. VSV-G-pseudotyped HIV-1 pNL4-3Luc(R-E-) was described previously [52]. The substrate peptide (Arg-Glu (EDANS)-Ser-Gln-Asn-Tyr-Pro -Ile-Val-Gln-Lys(DABCYL)-Arg) of HIV-1 protease was purchased from AnaSpec (Fremont, CA, USA). CCK8 Assay Kit was purchased from Beyotime (Nantong, China).

### 4.2. In Vitro Assay for HIV-1 Protease Inhibition

HIV-1 protease was cloned, heterologously expressed in *Escherichia coli*, and purified as described previously [53]. The HIV-1 PI activities of compounds were measured using FRET as described previously [42]. Compounds were dissolved in DMSO and diluted to appropriate concentrations. Protease and compounds were mixed in reaction buffer (0.1 M sodium acetate, 1 M sodium chloride, 1 mM ethylenediaminetetraacetic acid (EDTA), 1 mM dithiothreitol (DTT), 2% DMSO, and 1 mg/mL bovine serum albumin (BSA), at pH 4.7) in a 96-well plate and incubated for 20–30 min at room temperature, and then the substrate was added. Each reaction was recorded for about 10 min. Fluorescence readings were measured using Enspire (Perkin Elmer, Waltham, MA, USA) at excitation wavelength (λex) at 340 nm and emission wavelength (λem) at 490 nm.

### 4.3. Cytotoxicity Assay

The cytotoxicity of compounds was measured using the CCK8 Assay Kit [46]. HEK293T cells were treated with compounds at various concentrations. DMSO-treated cells were used as the control. Twenty-four hours post-treatment, the samples were subjected to the CCK8 Assay Kit following the manufacturer manual. The samples were analyzed at OD 450 using an EnVision multilabel reader (PerkinElmer, Waltham, MA, USA).

### 4.4. HIV-1 Infectivity Assay

HIV-1 infectivity assay was determined using a single-round HIV-1 infectivity assay [39,46]. HEK293T cells were co-transfected with either plasmid pNL4-3-E^-^R^-^ (pHIV-1_NL4-3_) or DRV-resistant pNL4-3-E^-^R^-^ variants (pHIV-1_DRV_^R^_S_) and pHCMV-G (VSV-G) to produce VSV-G-pseudotyped HIV-1. Compounds dissolved in DMSO and diluted to appropriate concentrations were added to culture medium at 5 h post-transfection. After incubation for 48 h at 37 °C, 10 μL supernatant was used to infect SupT1 cells. Forty-eight hours later, SupT1 cells were lysed, and firefly luciferase activities were determined using a firefly Luciferase Assay System (Promega, Madison, WI, USA).

### 4.5. Construction of DRV-Resistant pNL4-3-E^-^R^-^ Cloning (pHIV-1_DRV_^R^_S_)

To generate HIV-1 clones carrying the intended mutations, a site-directed mutagenesis kit (SBS Genetech) was used. V32I, L33F, I54M, and I84V mutations in the protease were introduced into pNL4-3-E^-^R^-^ according to the manufacturer’s instructions [11]. The primers used for mutations were 32/33 (F: 5′-ACAGGAGCAGATGATACAATATTTGAAGAAATGAATTTGCCA-3′, R: 5′-TGGCAAATTCATTTCTTCAAATATTGTATCATCTGCTCCTGT-3′), 54 (F: 5′-GGGAATTGGAGGTTTTATGAAAGTAAGACAGTATGAT-3′, R: 5′-ATCATACTGTCTTACTTTCATAAAACCTCCAATTCCC-3′), and 84 (F: 5′-GGACCTACACCTGTCAACGTAATTGGAAGAAATCTGT-3′, R: 5′-ATCATACTGTCTTACTTTCATAAAACCTCCAATTCCC-3′). The plasmids were sequenced by BBI Life Sciences Corporation. All desired mutations, but no unintended mutations, were found.

### 4.6. Molecular Modeling

The docking was performed through the “DOCK” module in the MOE using the alpha triangle placement method. Refinement of the docked poses was carried out using the Forcefield refinement scheme and scored using both the affinity dG and the London dG scoring system [43]. The HIV-1 protease crystal structure (PDB-ID: 4mc9) was obtained from the Protein Data Bank [46].

### 4.7. Binding Assay by SPR

Compound solutions with a series of increasing concentrations (0–50 μM at 2-fold dilution) were applied to all four channels at a flow rate of 30 μL/min. Purified HIV-1 protease was immobilized on a CM5 sensor chip using standard amine coupling with running buffer HBS-EP+ (10.5 mM HEPES, 157.5 mM NaCl, 3.15 mM EDTA, 0.0525% surfactant P-20, pH 7.4) using a Biacore T200 instrument. HIV-1 protease was immobilized to flow channel 2, and the immobilization level of flow channel 2 was ~3800 RU. The resulting data were fit to a 1:1 binding model using Biacore T200 evaluation software 2.0.

## Figures and Tables

**Table 1:**
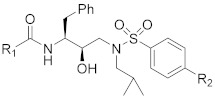
HIV-1 protease inhibitory and antiviral activity, and cytotoxicity of inhibitors **15a**−**17i**.

Comp.	R_1_	R_2_	IC_50_ (nM) ^a^	CC_50_ (μM) ^b^
**15a**	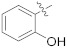	OCH_3_	3.34 ± 0.28	>100
**15b**	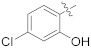	OCH_3_	7.82 ± 0.93	33.86
**15c**	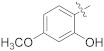	OCH_3_	4.68 ± 0.74	>100
**15d**	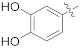	OCH_3_	0.0059 ± 0.0007	38.22
**15e**	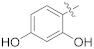	OCH_3_	0.30 ± 0.08	78.76
**15f**	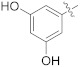	OCH_3_	0.0024 ± 0.0004	>100
**15g**	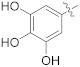	OCH_3_	1.13 ± 0.23	>100
**15h**	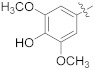	OCH_3_	0.08 ± 0.03	84.66
**15i**	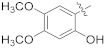	OCH_3_	1.41 ± 0.43	>100
**16a**	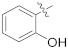	NH_2_	0.54 ± 0.11	>100
**16b**	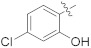	NH_2_	0.31 ± 0.08	>100
**16c**	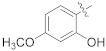	NH_2_	4.14 ± 1.38	>100
**16d**	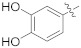	NH_2_	1.63 ± 0.33	>100
**16e**	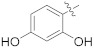	NH_2_	0.43 ± 0.08	>100
**16f**	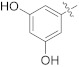	NH_2_	0.27 ± 0.03	>100
**16g**	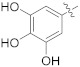	NH_2_	68.16 ± 21.35	57.64
**16h**	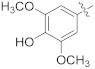	NH_2_	0.04 ± 0.003	>100
**16i**	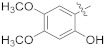	NH_2_	0.60 ± 0.18	>100
**17a**	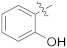	SCH_3_	3.97 ± 0.41	>100
**17b**	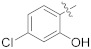	SCH_3_	2.79 ± 0.44	>100
**17c**	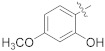	SCH_3_	8.21 ± 1.20	>100
**17d**	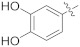	SCH_3_	0.0076 ± 0.0029	>100
**17e**	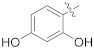	SCH_3_	0.40 ± 0.08	>100
**17f**	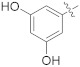	SCH_3_	0.0066 ± 0.0021	>100
**17h**	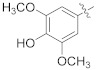	SCH_3_	0.32 ± 0.03	>100
**17i**	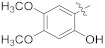	SCH_3_	4.71 ± 0.18	>100
DRV	-	-	0.51 ± 0.17	>100

^a^ All assays were conducted in triplicate, and the data shown represent mean values (±SD) derived from the results of three independent experiments. ^b^ All assays were conducted in triplicate.

**Table 2:** Effects of inhibitors on late-stage HIV-1.

Compounds	Inhibition (%) (10 μM) ^a^	Compounds	Inhibition (%) (10 μM) ^a^
**15a**	98 ± 2	**16c**	90 ± 6
**15b**	96 ± 3	**16d**	100
**15c**	92 ± 5	**16e**	75 ± 8
**15d**	100	**16f**	99 ± 2
**15e**	75 ± 6	**16g**	55 ± 6
**15f**	100	**16h**	99 ± 3
**15g**	99 ± 1	**17d**	100
**15h**	99 ± 2	**17f**	100
**16a**	100	DRV	100
**16b**	98 ± 2	DMSO	0

^a^ All assays were conducted in quadruplicate.

**Table 3:** Antiviral activity of **15b**, **15d**, and **15f** against multidrug-resistant HIV-1 variants.

Compounds	cLogP ^a^	Mean EC_50_ (nM, ± SD) ^b^		Fold Resistance ^c^
		HIV-1_NL4−3_	HIV_DRV_^R^s	
**15b**	6.695	1402 ± 23.00	1843 ± 39.60	1.31
**15d**	4.303	15.36 ± 2.17	402.4 ± 47.32	26.19
**15f**	4.372	28.89 ± 9.21	45.53 ± 12.11	1.57
DRV	2.887	1.80 ± 0.73	136.80 ± 1.12	76

^a^ cLogP values were calculated using ChemDraw 14. ^b^ All assays were conducted in triplicate, and the data shown represent mean values (±1 SD) derived from the results of three independent experiments. ^c^ Fold resistance is defined as EC_50_ (mutant)/EC_50_ (WT).

## Data Availability

Not applicable.

## Supplementary Materials

The supporting information can be downloaded at: https://www.mdpi.com/article/10.3390/ijms232214178/s1.

## References

[1] Friedman-Kien A.E. (1981). Disseminated Kaposi’s sarcoma syndrome in young homosexual men. J. Am. Acad. Dermatol..

[2] Bevan R.J., Harrison P.T.C. (2017). Threshold and non-threshold chemical carcinogens: A survey of the present regulatory landscape. Regul. Toxicol. Pharmacol..

[3] Palmisano L., Vella S. (2011). A brief history of antiretroviral therapy of HIV infection: Success and challenges. Ann. Ist. Super. Sanita.

[4] Montaner J.S., Lima V.D., Barrios R., Yip B., Wood E., Kerr T., Shannon K., Harrigan P.R., Hogg R.S., Daly P. (2010). Association of highly active antiretroviral therapy coverage, population viral load, and yearly new HIV diagnoses in British Columbia, Canada: A population-based study. Lancet.

[5] Braitstein P., Brinkhof M.W., Dabis F., Schechter M., Boulle A., Miotti P., Wood R., Laurent C., Sprinz E., Seyler C. (2006). Mortality of HIV-1-infected patients in the first year of antiretroviral therapy: Comparison between low-income and high-income countries. Lancet.

[6] Este J.A., Cihlar T. (2010). Current status and challenges of antiretroviral research and therapy. Antivir. Res..

[7] Ghosh A.K., Osswald H.L., Prato G. (2016). Recent Progress in the Development of HIV-1 Protease Inhibitors for the Treatment of HIV/AIDS. J. Med. Chem..

[8] Kohl N., Emini E., Schleif W., Davis L.J., Heimbach J.C., Dixon R., Scolnick E.M., Sigal I.S. (1988). Active human immunodeficiency virus protease is required for viral infectivity. Proc. Natl. Acad. Sci. USA.

[9] Mitsuya Y., Liu Tommy F., Rhee S.Y., Fessel W.J., Shafer Robert W. (2007). Prevalence of Darunavir Resistance–Associated Mutations: Patterns of Occurrence and Association with Past Treatment. J. Infect. Dis..

[10] Ghosh A.K., Rao K.V., Nyalapatla P.R., Osswald H.L., Martyr C.D., Aoki M., Hayashi H., Agniswamy J., Wang Y.-F., Bulut H. (2017). Design and Development of Highly Potent HIV-1 Protease Inhibitors with a Crown-Like Oxotricyclic Core as the P2-Ligand To Combat Multidrug-Resistant HIV Variants. J. Med. Chem..

[11] Aoki M., Das D., Hayashi H., Aoki-Ogata H., Takamatsu Y., Ghosh A.K., Mitsuya H. (2018). Mechanism of Darunavir (DRV)’s High Genetic Barrier to HIV-1 Resistance: A Key V32I Substitution in Protease Rarely Occurs, but Once It Occurs, It Predisposes HIV-1 To Develop DRV Resistance. MBio.

[12] Ghosh A.K., Nyalapatla P.R., Kovela S., Rao K.V., Brindisi M., Osswald H.L., Amano M., Aoki M., Agniswamy J., Wang Y.-F. (2018). Design and Synthesis of Highly Potent HIV-1 Protease Inhibitors Containing Tricyclic Fused Ring Systems as Novel P2 Ligands: Structure-Activity Studies, Biological and X-ray Structural Analysis. J. Med. Chem..

[13] Ghosh A.K., Williams J.N., Ho R.Y., Simpson H.M., Hattori S.-I., Hayashi H., Agniswamy J., Wang Y.-F., Weber I.T., Mitsuya H. (2018). Design and Synthesis of Potent HIV-1 Protease Inhibitors Containing Bicyclic Oxazolidinone Scaffold as the P2 Ligands: Structure-Activity Studies and Biological and X-ray Structural Studies. J. Med. Chem..

[14] Brik A., Wong C.H. (2003). HIV-1 protease: Mechanism and drug discovery. Org. Biomol. Chem..

[15] Lefebvre E., Schiffer C.A. (2008). Resilience to resistance of HIV-1 protease inhibitors: Profile of darunavir. AIDS Rev..

[16] Berti F., Frecer V., Miertus S. (2014). Inhibitors of HIV-protease from computational design. A history of theory and synthesis still to be fully appreciated. Curr. Pharm. Des..

[17] Ghosh A.K., Chapsal B.D., Weber I.T., Mitsuya H. (2008). Design of HIV Protease Inhibitors Targeting Protein Backbone: An Effective Strategy for Combating Drug Resistance. Acc. Chem. Res..

[18] Ghosh A.K., Sridhar P.R., Leshchenko S., Hussain A.K., Li J., Kovalevsky A.Y., Walters D.E., Wedekind J.E., Grum-Tokars V., Das D. (2006). Structure-based design of novel HIV-1 protease inhibitors to combat drug resistance. J. Med. Chem..

[19] Ghosh A.K., Ramu Sridhar P., Kumaragurubaran N., Koh Y., Weber I.T., Mitsuya H. (2006). Bis-Tetrahydrofuran: A Privileged Ligand for Darunavir and a New Generation of HIV Protease Inhibitors That Combat Drug Resistance. ChemMedChem.

[20] Ghosh A.K., Anderson D.D., Weber I.T., Mitsuya H. (2012). Enhancing protein backbone binding--a fruitful concept for combating drug-resistant HIV. Angew. Chem..

[21] Ma Y., Frutos-Beltrán E., Kang D., Pannecouque C., De Clercq E., Menéndez-Arias L., Liu X., Zhan P. (2021). Medicinal chemistry strategies for discovering antivirals effective against drug-resistant viruses. Chem. Soc. Rev..

[22] Ghosh A.K., Yu X., Osswald H.L., Agniswamy J., Wang Y.-F., Amano M., Weber I.T., Mitsuya H. (2015). Structure-Based Design of Potent HIV-1 Protease Inhibitors with Modified P1-Biphenyl Ligands: Synthesis, Biological Evaluation, and Enzyme–Inhibitor X-ray Structural Studies. J. Med. Chem..

[23] Agniswamy J., Shen C.-H., Wang Y.-F., Ghosh A.K., Rao K.V., Xu C.-X., Sayer J.M., Louis J.M., Weber I.T. (2013). Extreme Multidrug Resistant HIV-1 Protease with 20 Mutations Is Resistant to Novel Protease Inhibitors with P1′-Pyrrolidinone or P2-Tris-tetrahydrofuran. J. Med. Chem..

[24] Meher B.R., Wang Y. (2012). Interaction of I50V mutant and I50L/A71V double mutant HIV-protease with inhibitor TMC114 (darunavir): Molecular dynamics simulation and binding free energy studies. J. Phys. Chem. B..

[25] Parai M.K., Huggins D.J., Cao H., Nalam M.N.L., Ali A., Schiffer C.A., Tidor B., Rana T.M. (2012). Design, synthesis, and biological and structural evaluations of novel HIV-1 protease inhibitors to combat drug resistance. J. Med. Chem..

[26] Mirani A., Kundaikar H., Velhal S., Patel V., Bandivdekar A., Degani M., Patravale V. (2019). Evaluation of Phytopolyphenols for their gp120-CD4 Binding Inhibitory Properties by In Silico Molecular Modelling & In Vitro Cell Line Studies. Curr. HIV Res..

[27] Calland N., Sahuc M.-E., Belouzard S., Pène V., Bonnafous P., Mesalam A.A., Deloison G., Descamps V., Sahpaz S., Wychowski C. (2015). Polyphenols Inhibit Hepatitis C Virus Entry by a New Mechanism of Action. J. Virol..

[28] Fassina G., Buffa A., Benelli R., Varnier O.E., Noonan D.M., Albini A. (2002). Polyphenolic antioxidant (-)-epigallocatechin-3-gallate from green tea as a candidate anti-HIV agent. AIDS.

[29] Hwang B.S., Lee I.-K., Choi H.J., Yun B.-S. (2015). Anti-influenza activities of polyphenols from the medicinal mushroom Phellinus baumii. Bioorg. Med. Chem. Lett..

[30] Kratz J.M., Andrighetti-Fröhner C.R., Kolling D.J., Leal P.C., Cirne-Santos C.C., Yunes R.A., Nunes R.J., Trybala E., Bergström T., Frugulhetti I.C.P.P. (2008). Anti-HSV-1 and anti-HIV-1 activity of gallic acid and pentyl gallate. Mem. Inst. Oswaldo Cruz..

[31] Ohba M., Oka T., Ando T., Arahata S., Ikegaya A., Takagi H., Ogo N., Zhu C., Owada K., Kawamori F. (2016). Antiviral effect of theaflavins against caliciviruses. J. Antibiot..

[32] de Oliveira A., Prince D., Lo C.Y., Lee L.H., Chu T.C. (2015). Antiviral activity of theaflavin digallate against herpes simplex virus type 1. Antivir. Res..

[33] Zu M., Yang F., Zhou W., Liu A., Du G., Zheng L. (2012). In vitro anti-influenza virus and anti-inflammatory activities of theaflavin derivatives. Antivir. Res..

[34] Chowdhury P., Sahuc M.-E., Rouillé Y., Rivière C., Bonneau N., Vandeputte A., Brodin P., Goswami M., Bandyopadhyay T., Dubuisson J. (2018). Theaflavins, polyphenols of black tea, inhibit entry of hepatitis C virus in cell culture. PLoS ONE.

[35] Yang J., Li L., Tan S., Jin H., Qiu J., Mao Q., Li R., Xia C., Jiang Z.-H., Jiang S. (2012). A natural theaflavins preparation inhibits HIV-1 infection by targeting the entry step: Potential applications for preventing HIV-1 infection. Fitoterapia.

[36] Wu Y.-H., Hao B.-J., Cao H.-C., Xu W., Li Y.-J., Li L.-J. (2012). Anti-hepatitis B virus effect and possible mechanism of action of 3,4-o-dicaffeoylquinic Acid in vitro and in vivo. Evid. Based Complement. Alternat. Med..

[37] Shin M., Kang E., Lee Y. (2005). A flavonoid from medicinal plants blocks hepatitis B virus-e antigen secretion in HBV-infected hepatocytes. Antivir. Res..

[38] Ghosh A.K., Leshchenko S., Noetzel M. (2004). Stereoselective Photochemical 1,3-Dioxolane Addition to 5-Alkoxymethyl-2(5H)-furanone:  Synthesis of Bis-tetrahydrofuranyl Ligand for HIV Protease Inhibitor UIC-94017 (TMC-114). J. Org. Chem..

[39] Zhu M., Ma L., Zhou H., Dong B., Wang Y., Wang Z., Zhou J., Zhang G., Wang J., Liang C. (2020). Preliminary SAR and biological evaluation of potent HIV-1 protease inhibitors with pyrimidine bases as novel P2 ligands to enhance activity against DRV-resistant HIV-1 variants. Eur. J. Med. Chem..

[40] Zhu M., Ma L., Wen J., Dong B., Wang Y., Wang Z., Zhou J., Zhang G., Wang J., Guo Y. (2020). Rational design and Structure-Activity relationship of coumarin derivatives effective on HIV-1 protease and partially on HIV-1 reverse transcriptase. Eur. J. Med. Chem..

[41] Nakano M., Sato Y. (1987). Rearrangement of (substituted benzyl)trimethylammonium ylides in a nonbasic medium: The improved Sommelet-Hauser rearrangement. J. Org. Chem..

[42] Matayoshi E., Wang G., Krafft G., Erickson J. (1990). Novel fluorogenic substrates for assaying retroviral proteases by resonance energy transfer. Science.

[43] Cer R.Z., Mudunuri U., Stephens R., Lebeda F.J. (2009). IC50-to-Ki: A web-based tool for converting IC50 to Ki values for inhibitors of enzyme activity and ligand binding. Nucleic Acids Res..

[44] Tominaga H., Ishiyama M., Ohseto F., Sasamoto K., Hamamoto T., Suzuki K., Watanabe M. (1999). A water-soluble tetrazolium salt useful for colorimetric cell viability assay. Anal. Commun..

[45] Ghosh A.K., Swanson L.M., Cho H., Leshchenko S., Hussain K.A., Kay S., Walters D.E., Koh Y., Mitsuya H. (2005). Structure-Based Design:  Synthesis and Biological Evaluation of a Series of Novel Cycloamide-Derived HIV-1 Protease Inhibitors. J. Med. Chem..

[46] Garcia J.-M., Gao A., He P.-L., Choi J., Tang W., Bruzzone R., Schwartz O., Naya H., Nan F.-J., Li J. (2009). High-throughput screening using pseudotyped lentiviral particles: A strategy for the identification of HIV-1 inhibitors in a cell-based assay. Antivir. Res..

[47] Mohammed A.F., Abdel-Moty S.G., Hussein M.A., Abdel-Alim A.A. (2013). Design, synthesis and molecular docking of some new 1,2,4-triazolobenzimidazol-3-yl acetohydrazide derivatives with anti-inflammatory-analgesic activities. Arch. Pharm. Res..

[48] Ganguly A.K., Alluri S.S., Wang C.-H., Antropow A., White A., Caroccia D., Biswas D., Kang E., Zhang L.-K., Carroll S.S. (2014). Structural optimization of cyclic sulfonamide based novel HIV-1 protease inhibitors to picomolar affinities guided by X-ray crystallographic analysis. Tetrahedron.

[49] Ghosh A.K., Rao K.V., Nyalapatla P.R., Kovela S., Brindisi M., Osswald H.L., Reddy B.S., Agniswamy J., Wang Y.-F., Aoki M. (2018). Design of Highly Potent, Dual-Acting and Central-Nervous-System-Penetrating HIV-1 Protease Inhibitors with Excellent Potency against Multidrug-Resistant HIV-1 Variants. ChemMedChem.

[50] Rusere L.N., Lockbaum G.J., Lee S.-K., Henes M., Kosovrasti K., Spielvogel E., Nalivaika E.A., Swanstrom R., Yilmaz N.K., Schiffer C.A. (2019). HIV-1 Protease Inhibitors Incorporating Stereochemically Defined P2’ Ligands To Optimize Hydrogen Bonding in the Substrate Envelope. J. Med. Chem..

[51] Miller J.F., Andrews C.W., Brieger M., Furfine E.S., Hale M.R., Hanlon M.H., Hazen R.J., Kaldor I., McLean E.W., Reynolds D. (2006). Ultra-potent P1 modified arylsulfonamide HIV protease inhibitors: The discovery of GW0385. Bioorg. Med. Chem. Lett..

[52] Akkina R.K., Walton R.M., Chen M.L., Li Q.X., Planelles V., Chen I.S. (1996). High-efficiency gene transfer into CD34+ cells with a human immunodeficiency virus type 1-based retroviral vector pseudotyped with vesicular stomatitis virus envelope glycoprotein G. J. Virol..

[53] Dergousova N.I., Amerik A., Volynskaya A.M., Rumsh L.D. (1996). HIV-I protease. Cloning, expression, and purification. Appl. Biochem. Biotechnol..

