# Supplementary material for: Design and Evaluation of Novel HIV-1 Protease Inhibitors Containing Phenols or Polyphenols as P2 Ligands with High Activity against DRV-Resistant HIV-1 Variants
Source: Int J Mol Sci. 2022 Nov 16;23(22):14178. doi: 10.3390/ijms232214178 (PMC9697080; doi:10.3390/ijms232214178)
Supplement: Supplementary file 1 [file ijms-23-14178-s001.zip › ijms-1983362-supplementary.pdf]

Design and Evaluation of Novel HIV-1 Protease Inhibitors Containing Phenols or Polyphenols as P2 Ligands with High Activity against DRV-Resistant HIV-1 Variants

Ling Ma [1,†], Jiajia Wen [1,†], Biao Dong [1], Jinming Zhou [2], Shangjiu Hu [1], Juxian Wang [1], Yucheng Wang [1], Mei Zhu [1,*] and Shan Cen [1,*]

1 Institute of Medicinal Biotechnology, Chinese Academy of Medical Science and Peking Union Medical College, Beijing 100050, China; maling26@imb.pumc.edu.cn (L.M.); 18801344648@163.com (J.W.); blizzarddon@vip.sina.com (B.D.); hu_shangjiu@163.com (S.H.); imbjxwang@163.com (J.W.); wangyucheng@imb.pumc.edu.cn (Y.W.)
2 Key Laboratory of the Ministry of Education for Advanced Catalysis Materials, Department of Chemistry, Zhejiang Normal University, Jinhua 321004, China; zhoujinming@zjnu.edu.cn
* Correspondence: zhumei@imb.pumc.edu.cn (M.Z.); shancen@imb.pumc.edu.cn (S.C.)
† These authors contributed equally to this work.

Table of Contents

I. ^{1}H NMR, ^{13}C NMR and HR MS Spectrums of Compounds S2–S48

II. Chemistry S49–S67

I. ^{1}H NMR, ^{13}C NMR and HR MS Spectrums of Compounds

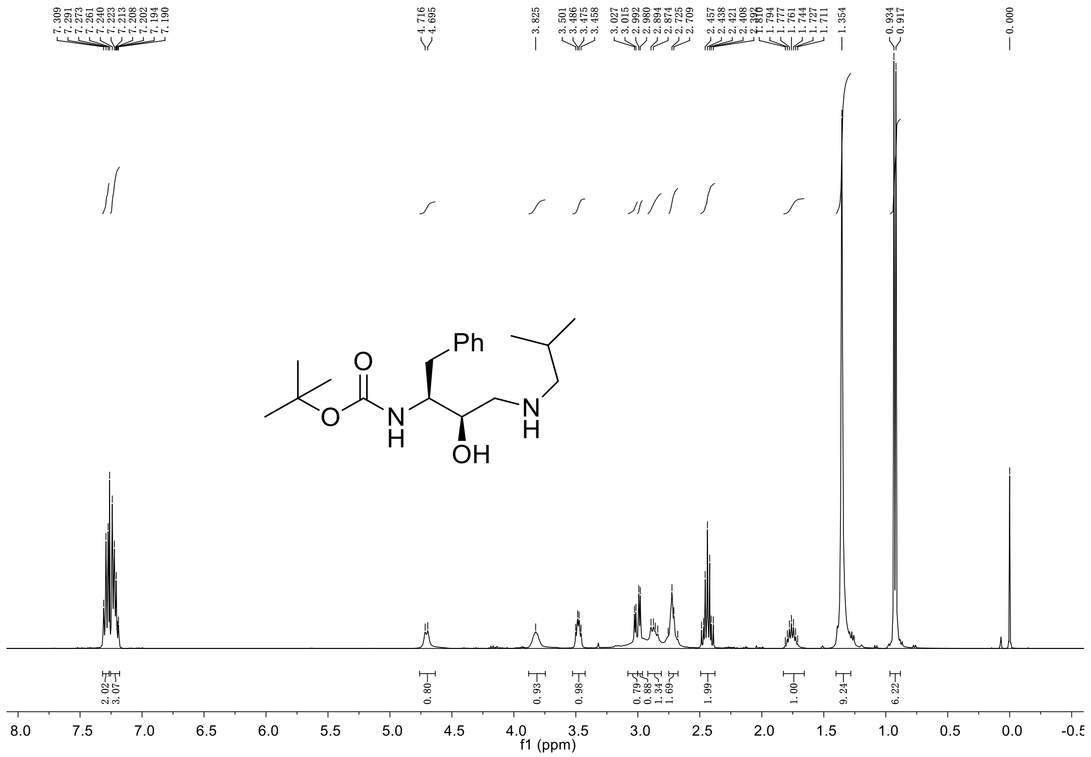

^{1}H NMR Spectrum of compound **3**

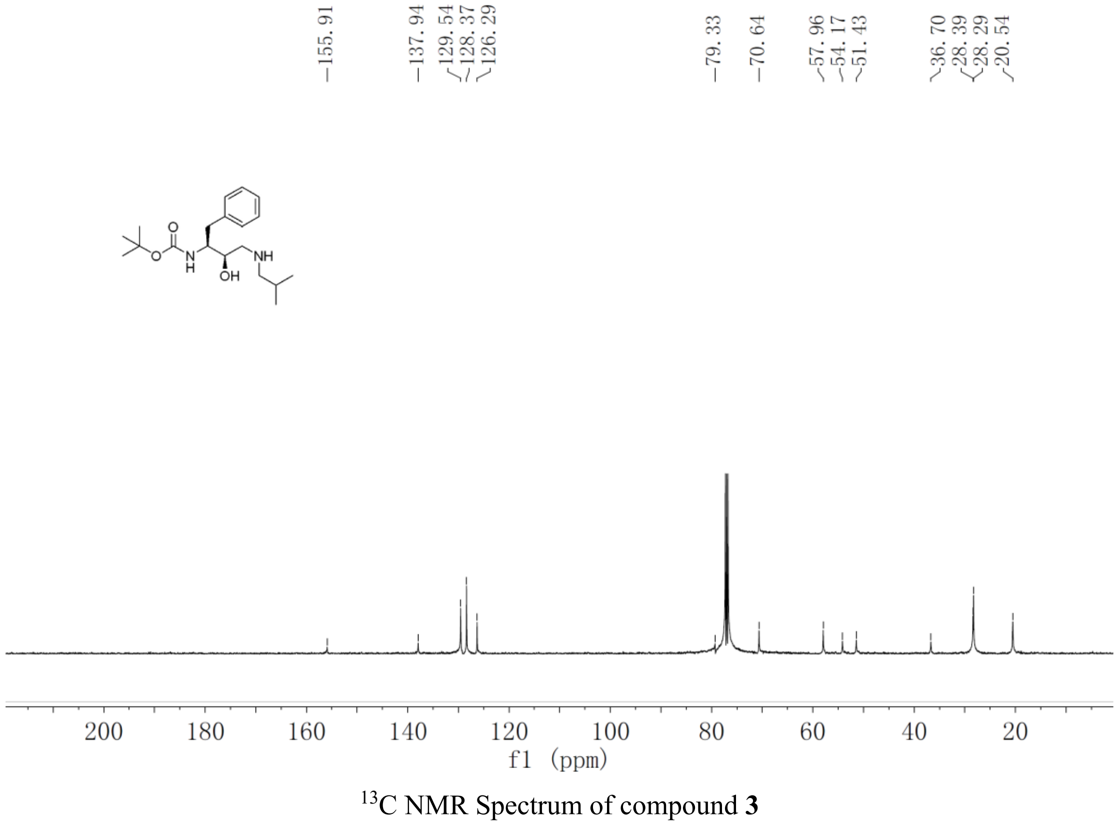

^{13}C NMR Spectrum of compound **3**

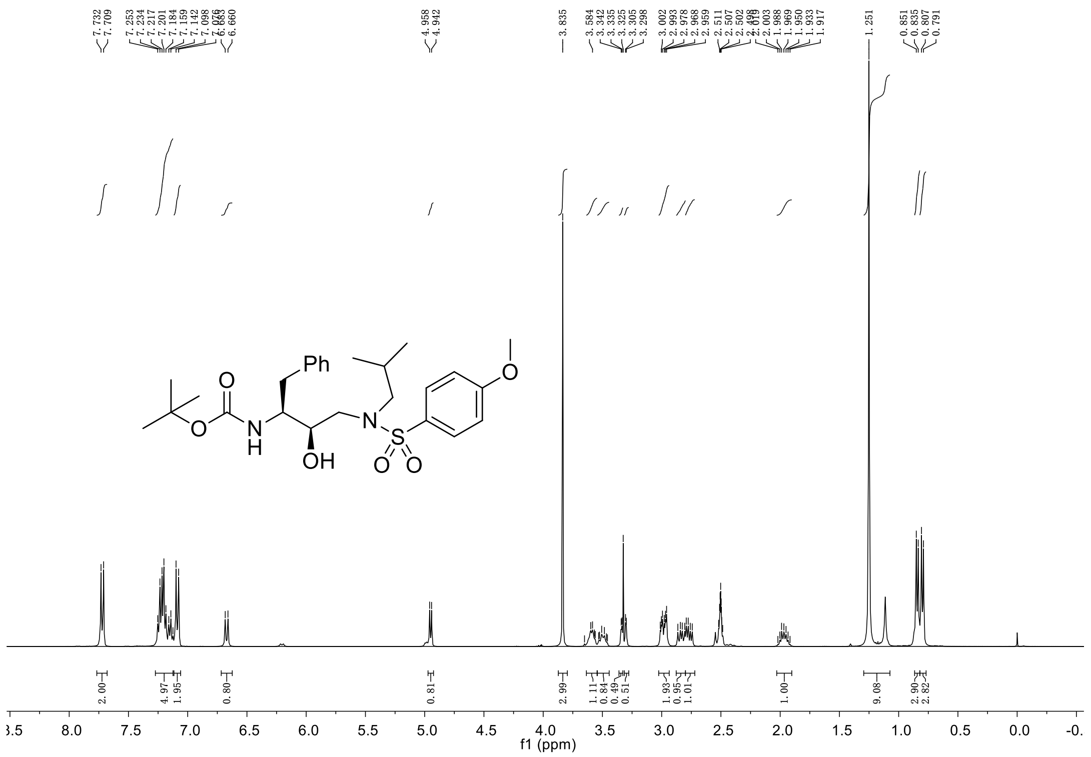

^{1}H NMR Spectrum of compound **7**

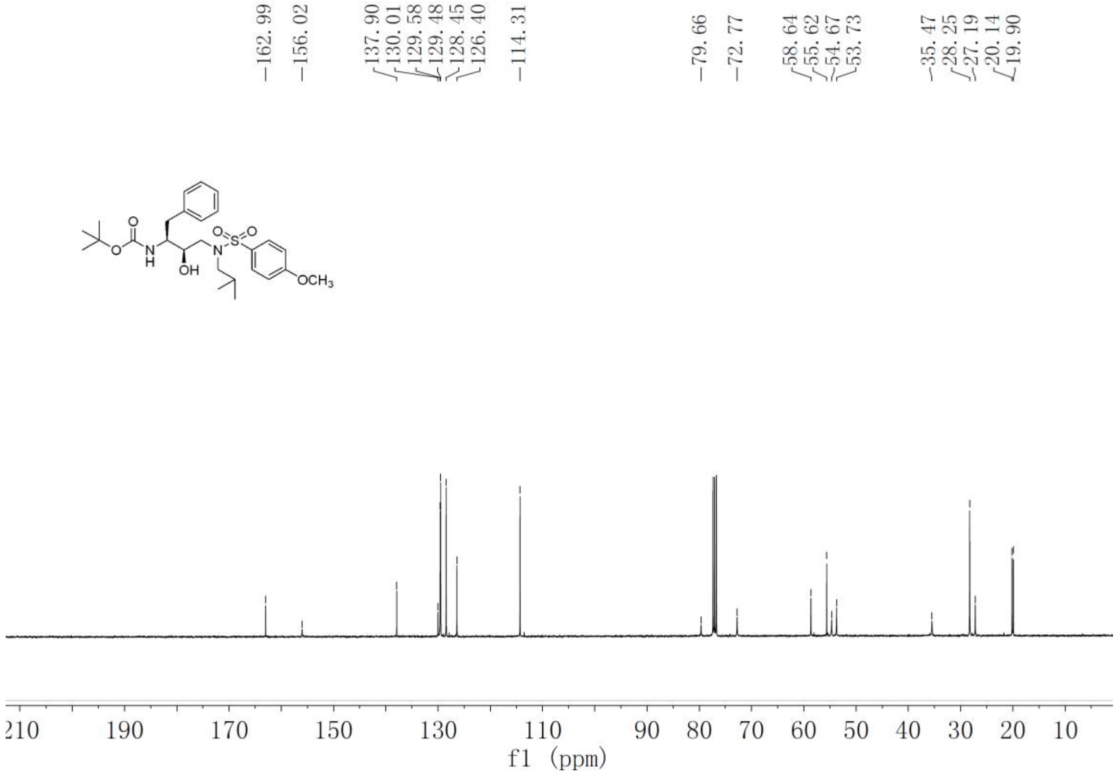

^{13}C NMR Spectrum of compound **7**

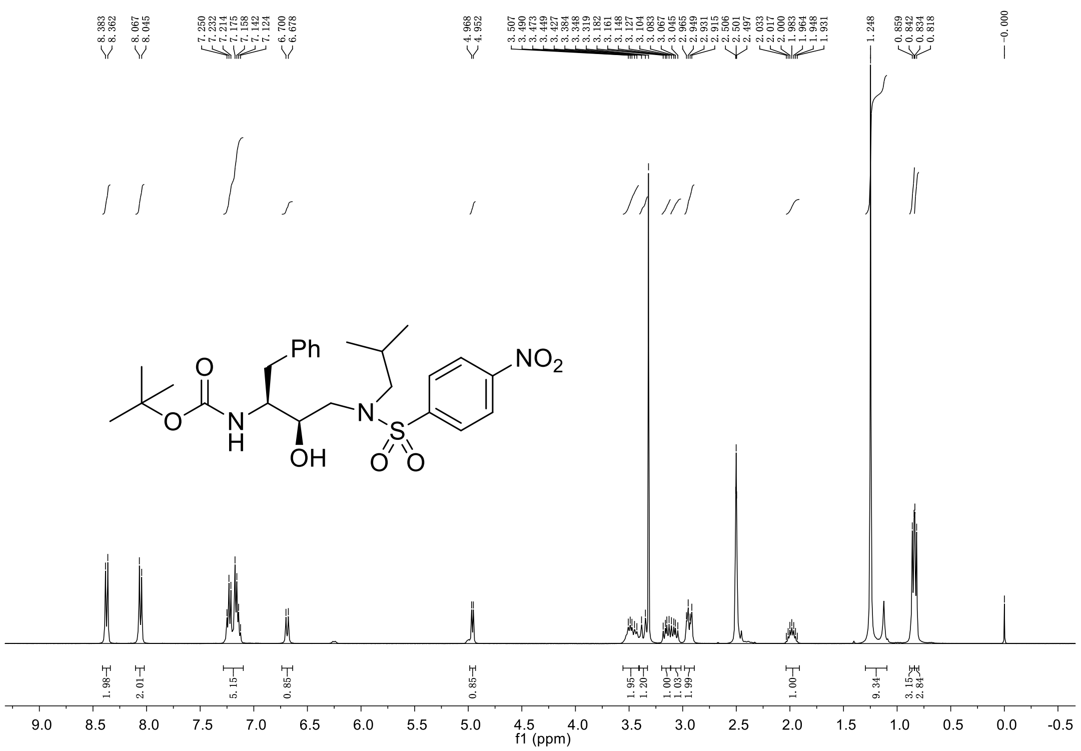

^{1}H NMR Spectrum of compound **8**

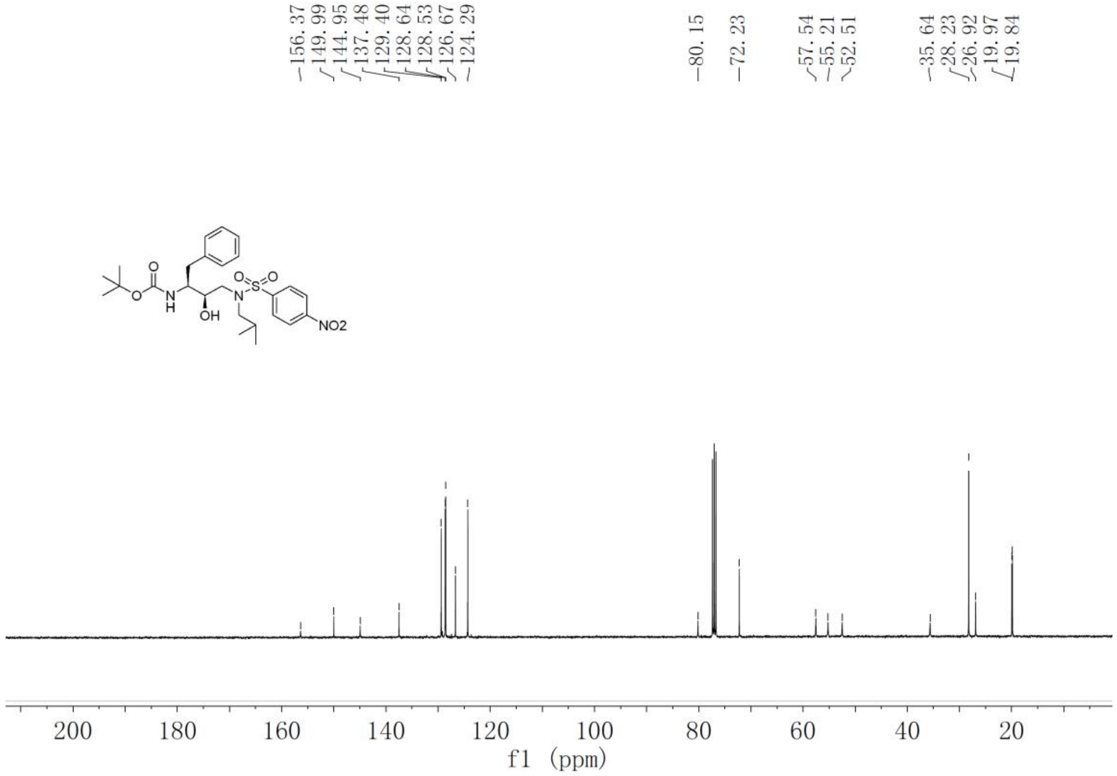

^{13}C NMR Spectrum of compound **8**

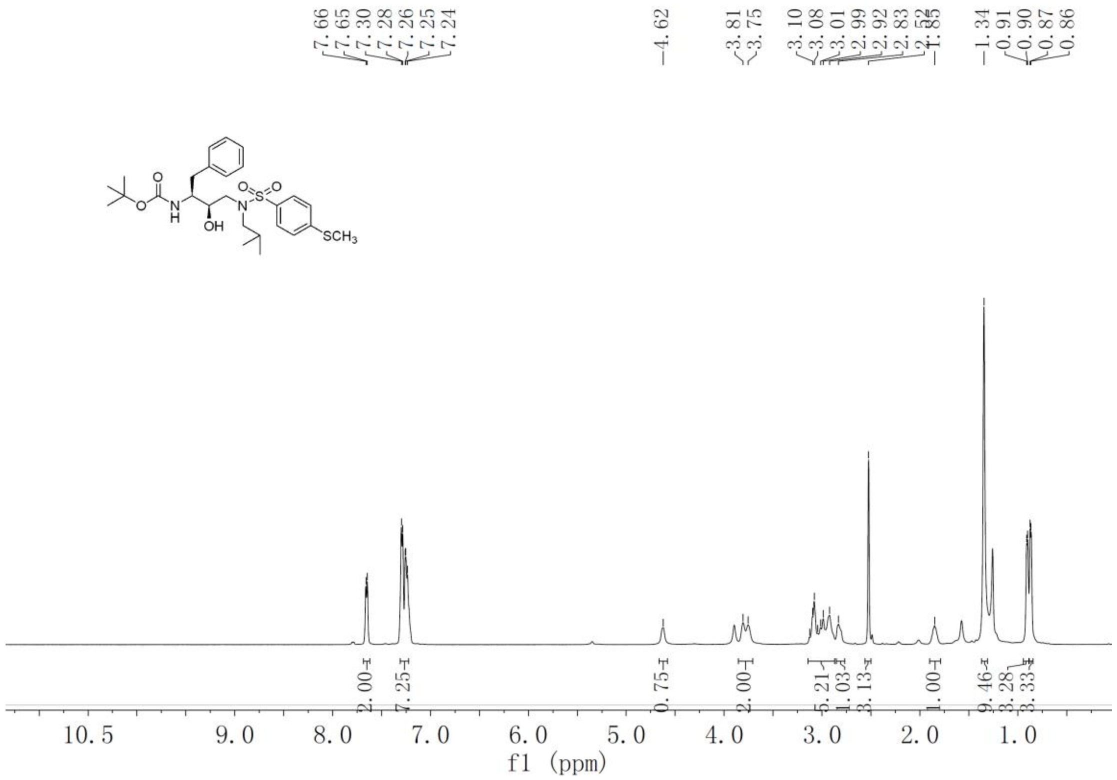

^{1}H NMR Spectrum of compound **9**

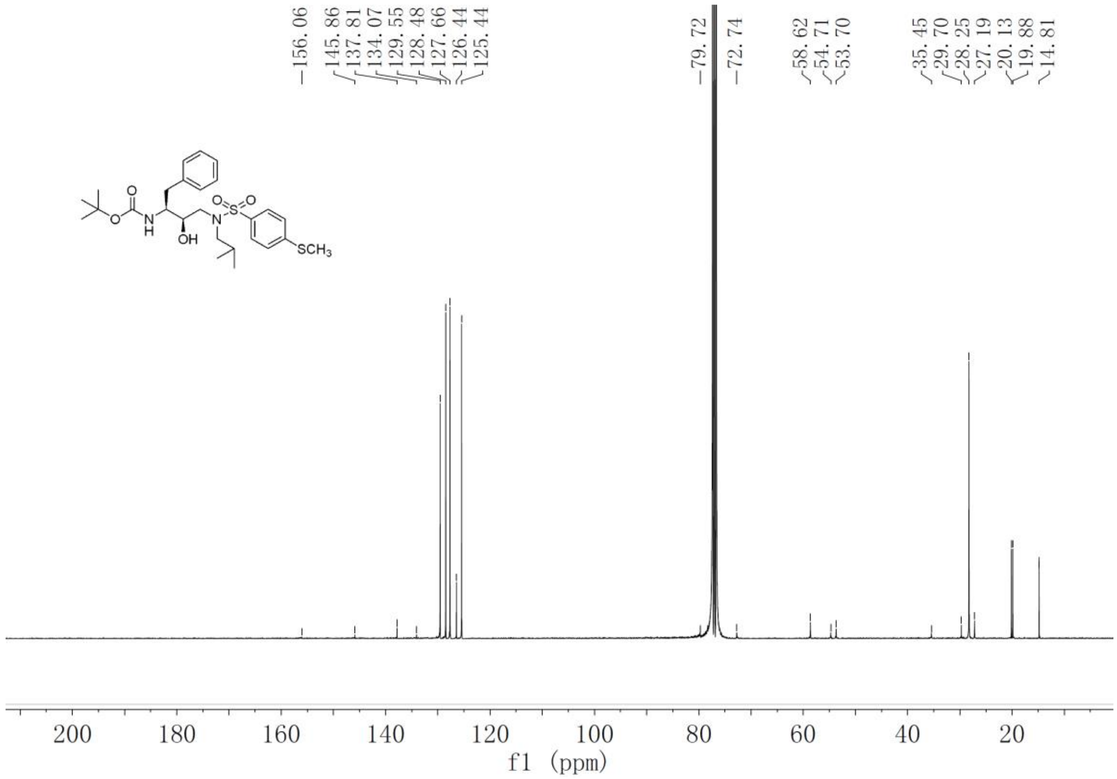

^{13}C NMR Spectrum of compound **9**

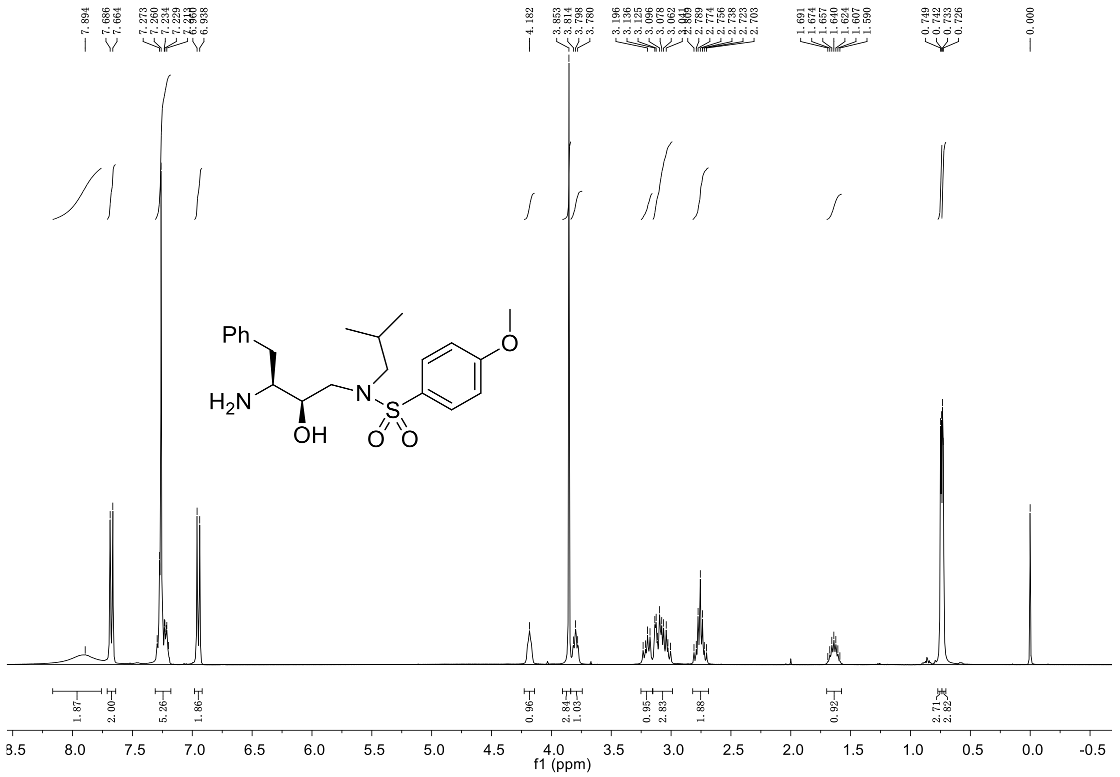

^{1}H NMR Spectrum of compound **10**

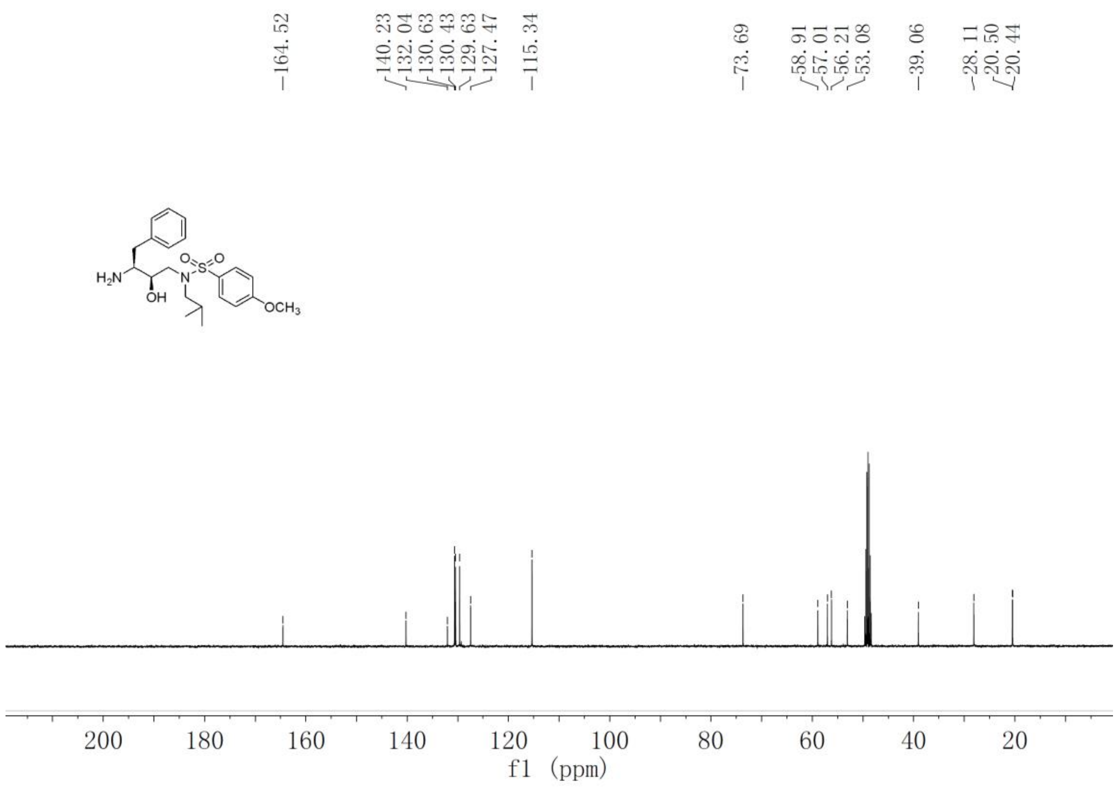

^{13}C NMR Spectrum of compound **10**

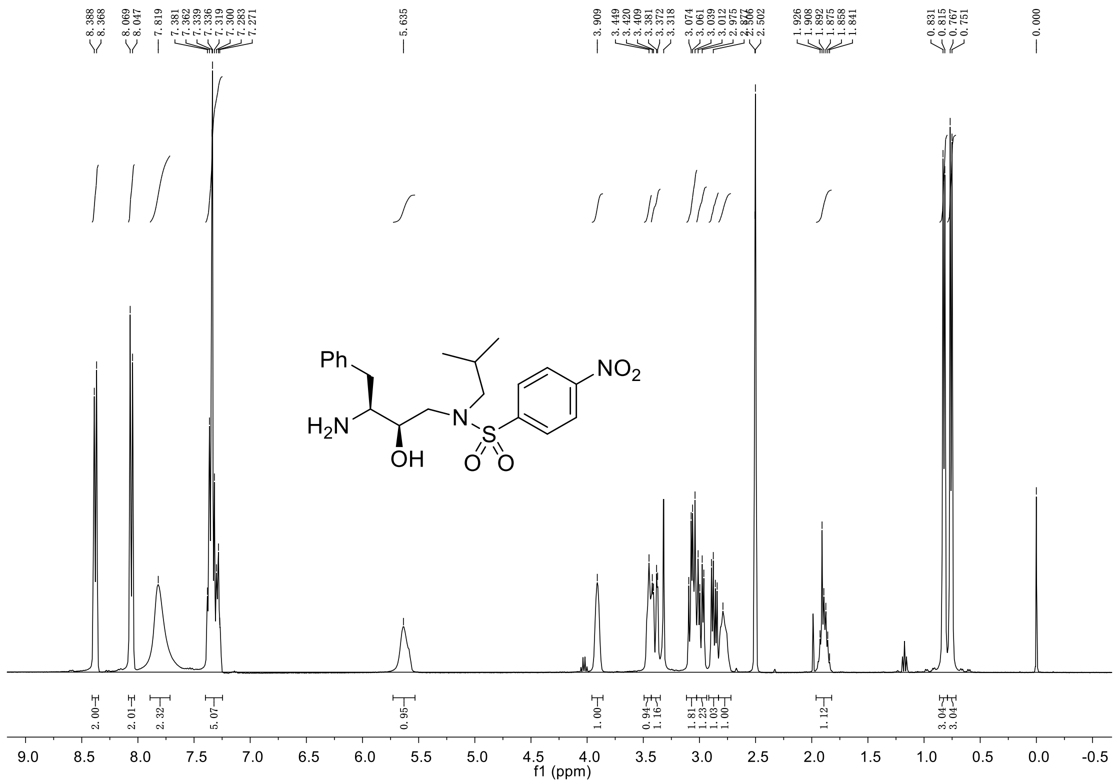

^{1}H NMR Spectrum of compound **11**

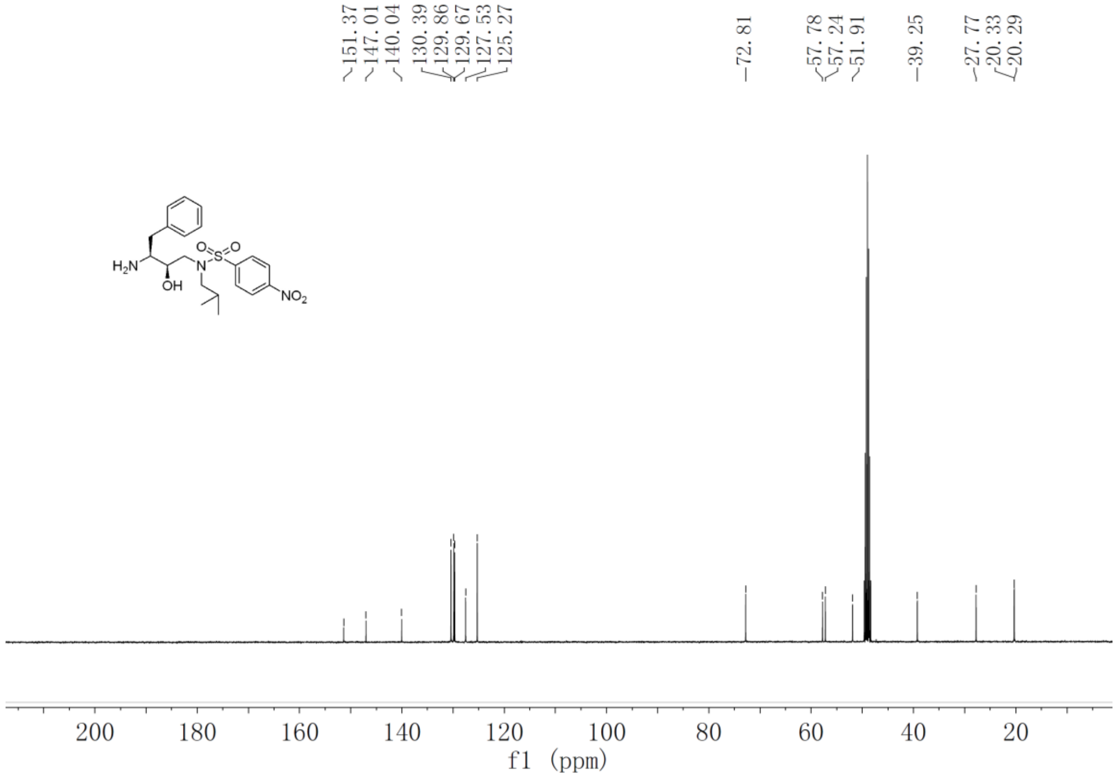

^{13}C NMR Spectrum of compound **11**

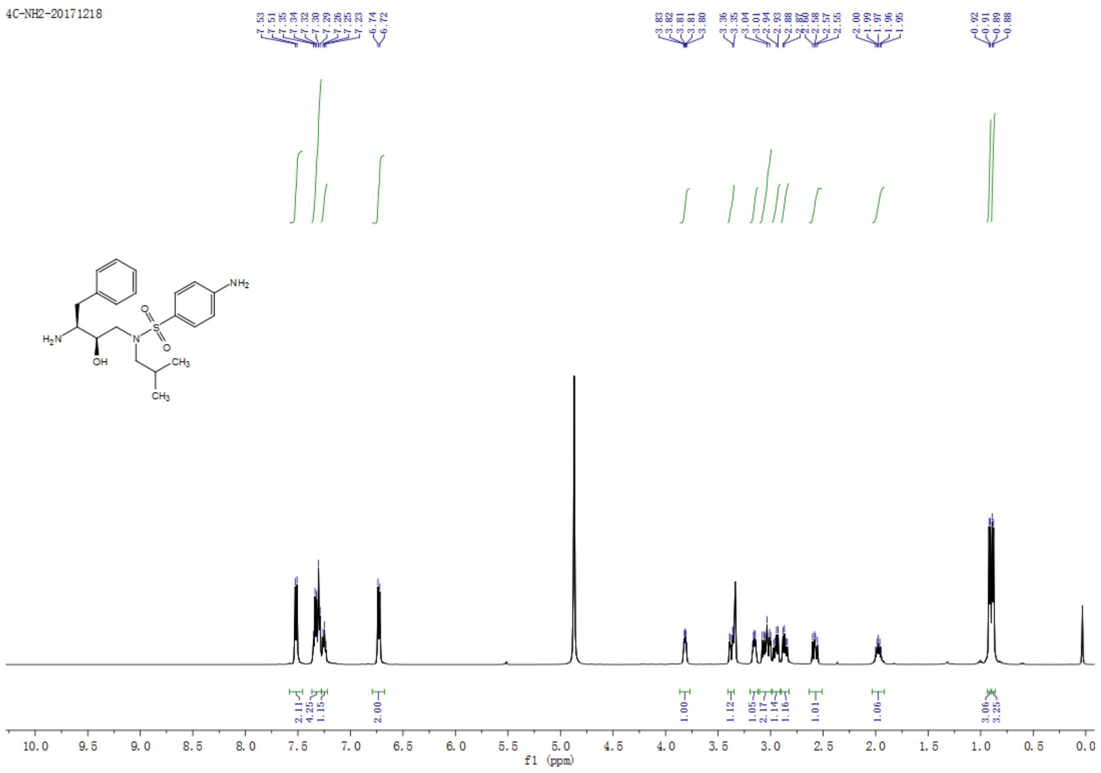

^{1}H NMR Spectrum of compound **12**

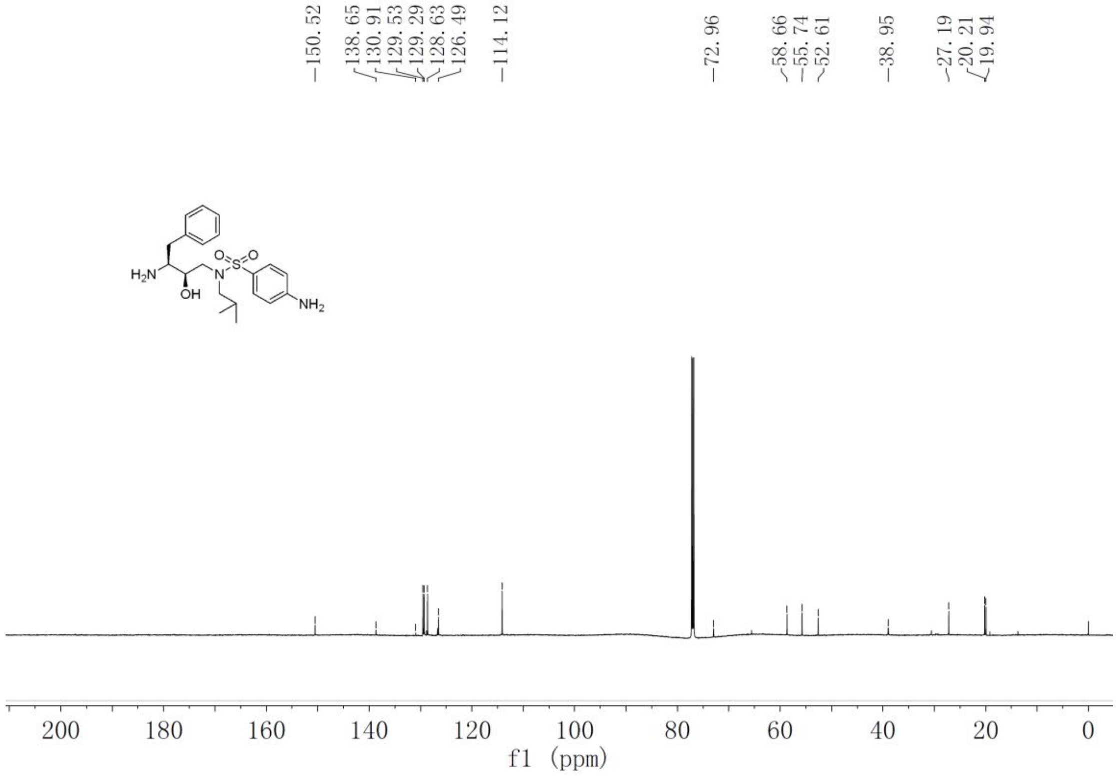

^{13}C NMR Spectrum of compound **12**

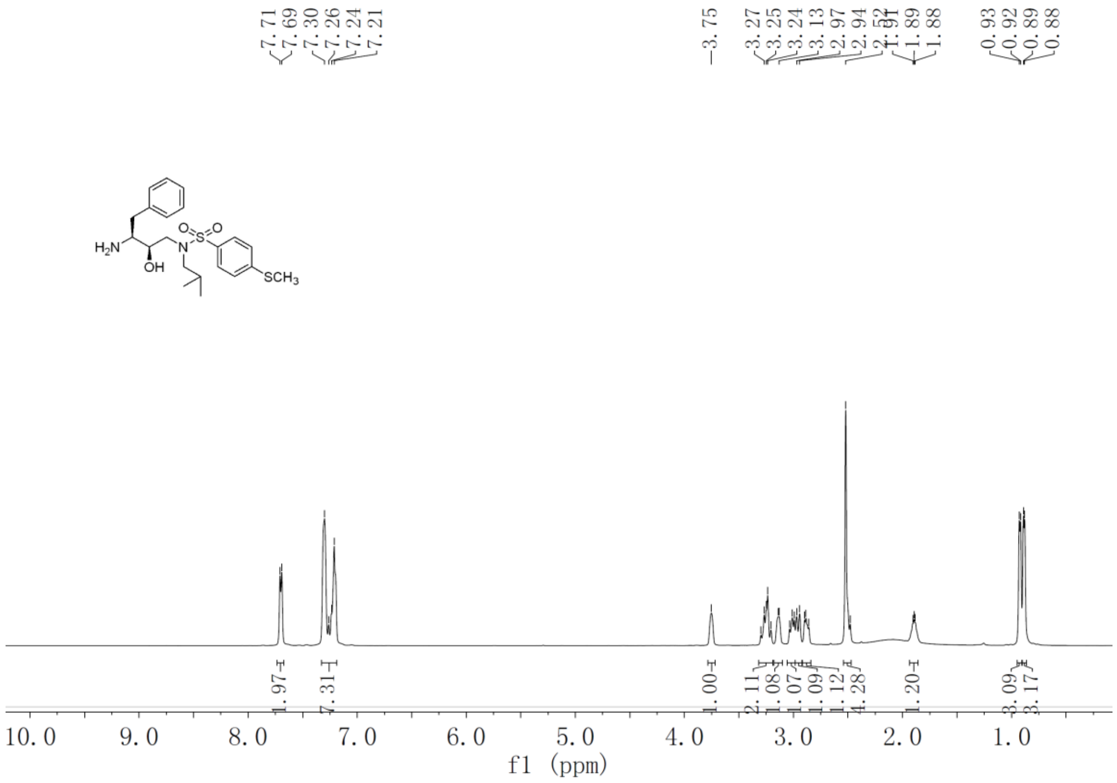

^{1}H NMR Spectrum of compound **13**

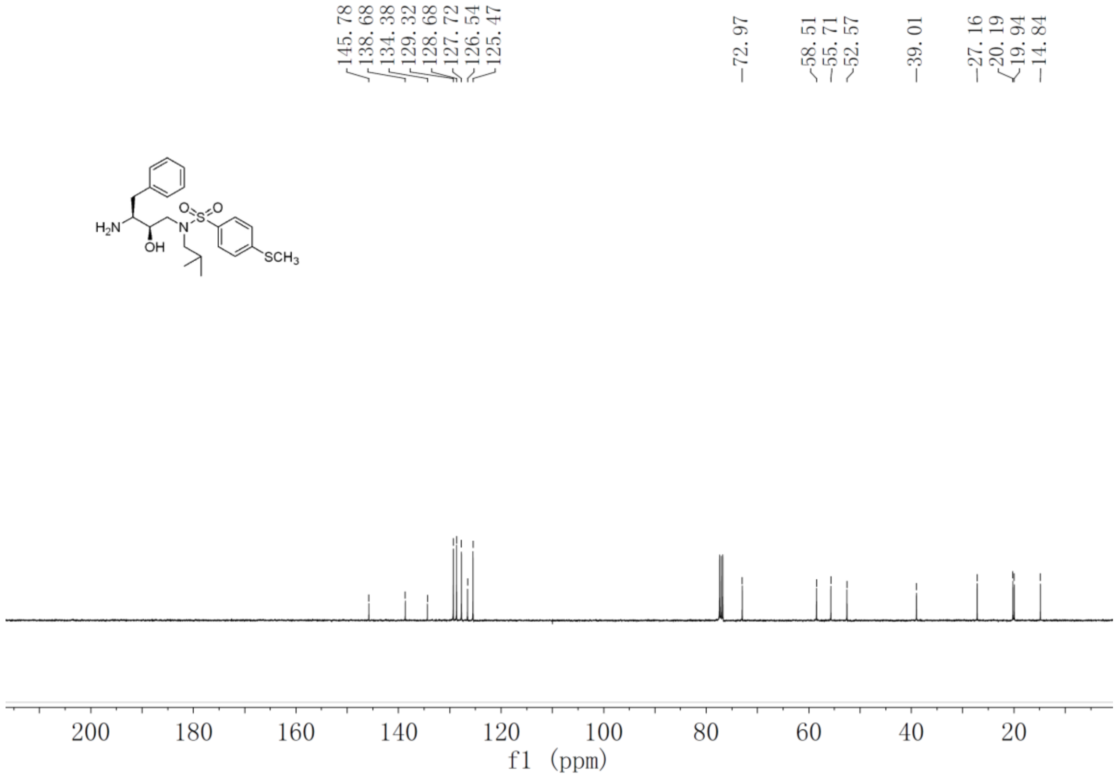

^{13}C NMR Spectrum of compound **13**

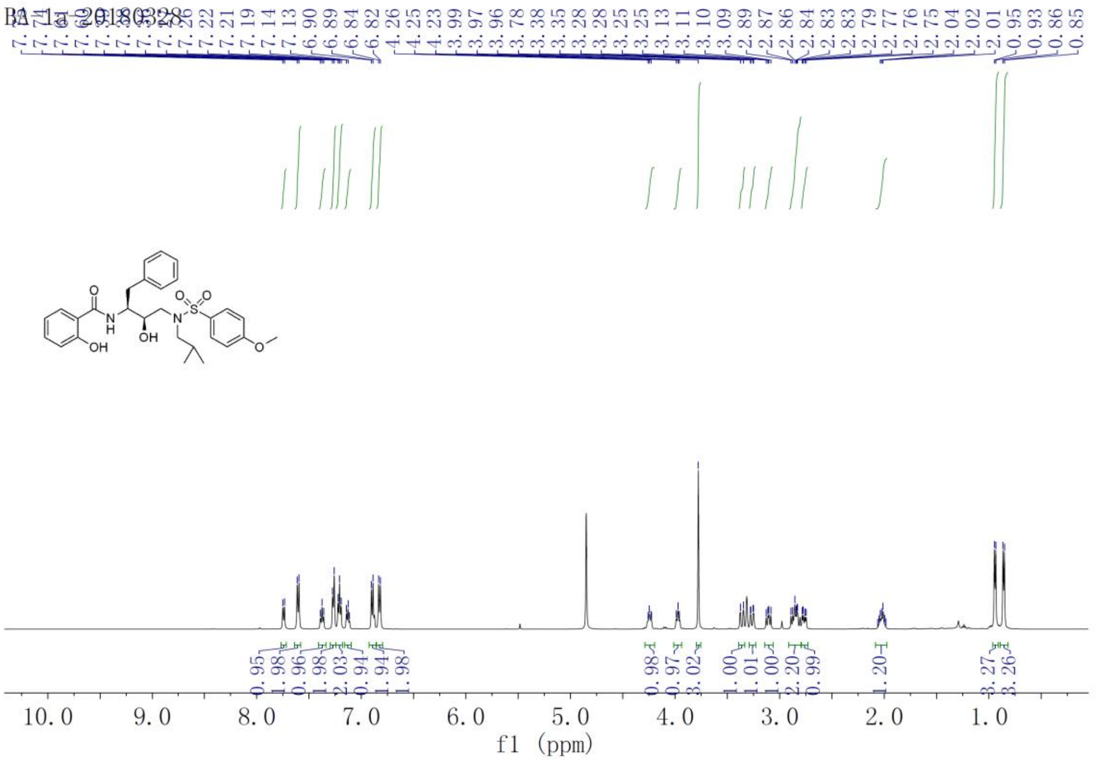

^{1}H NMR Spectrum of compound **15a**

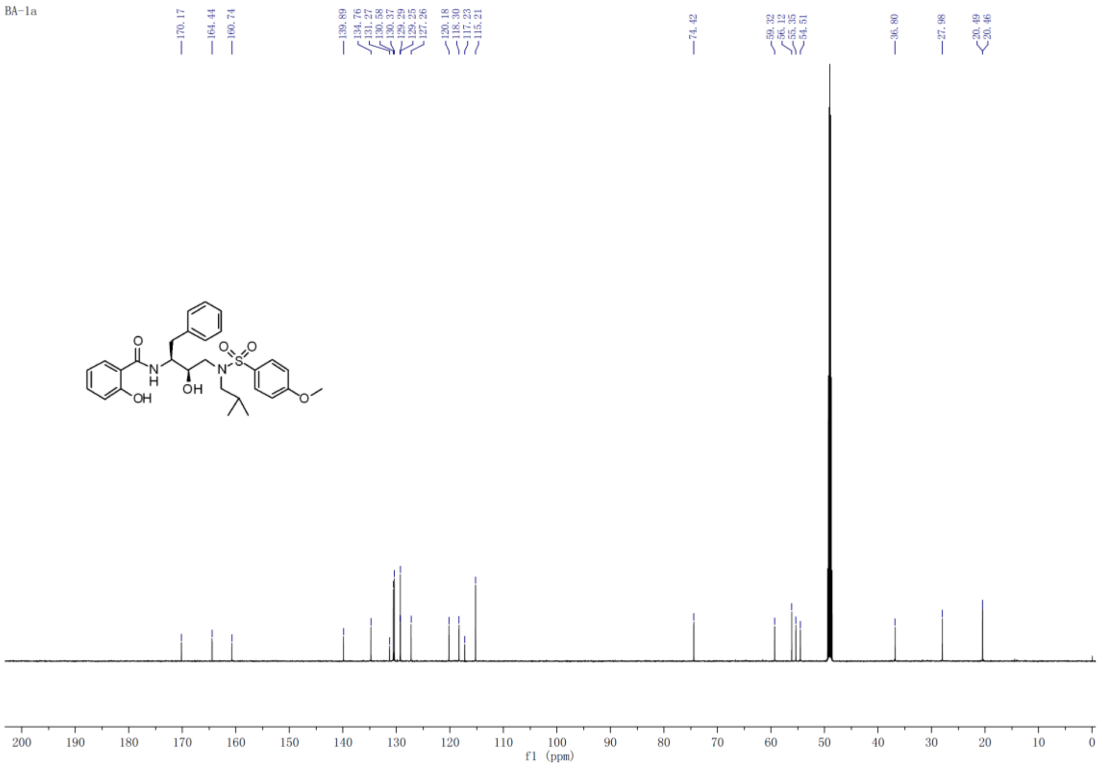

^{13}C NMR Spectrum of compound **15a**

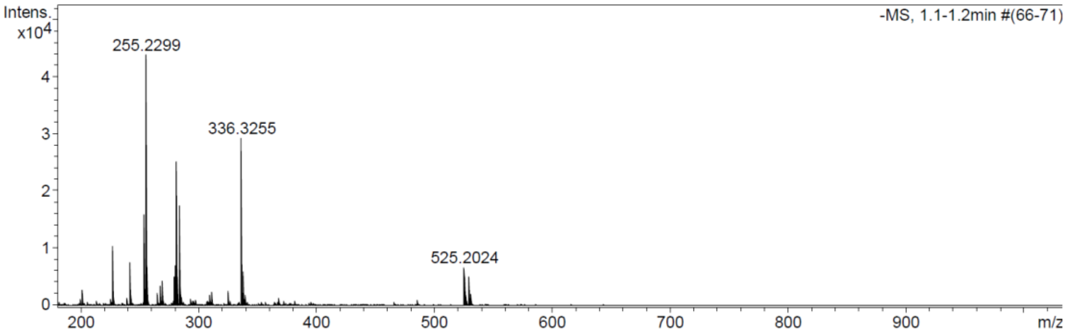

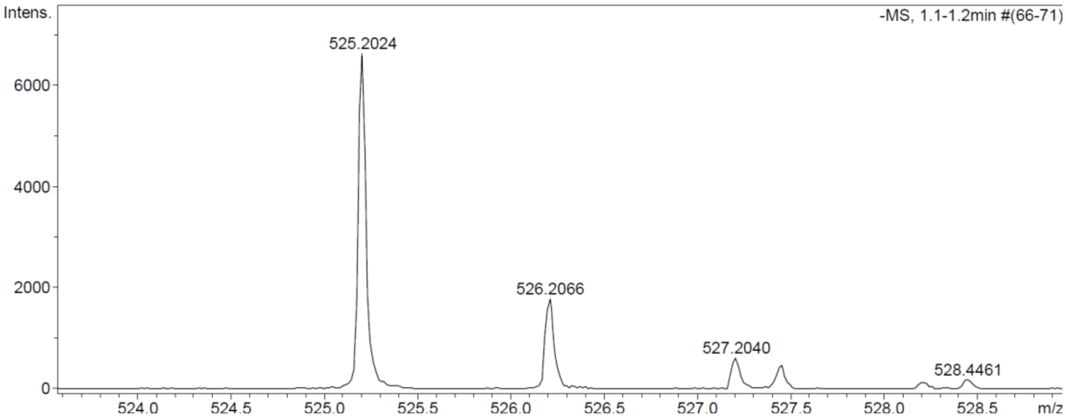

HR MS Spectrum of compound **15a**

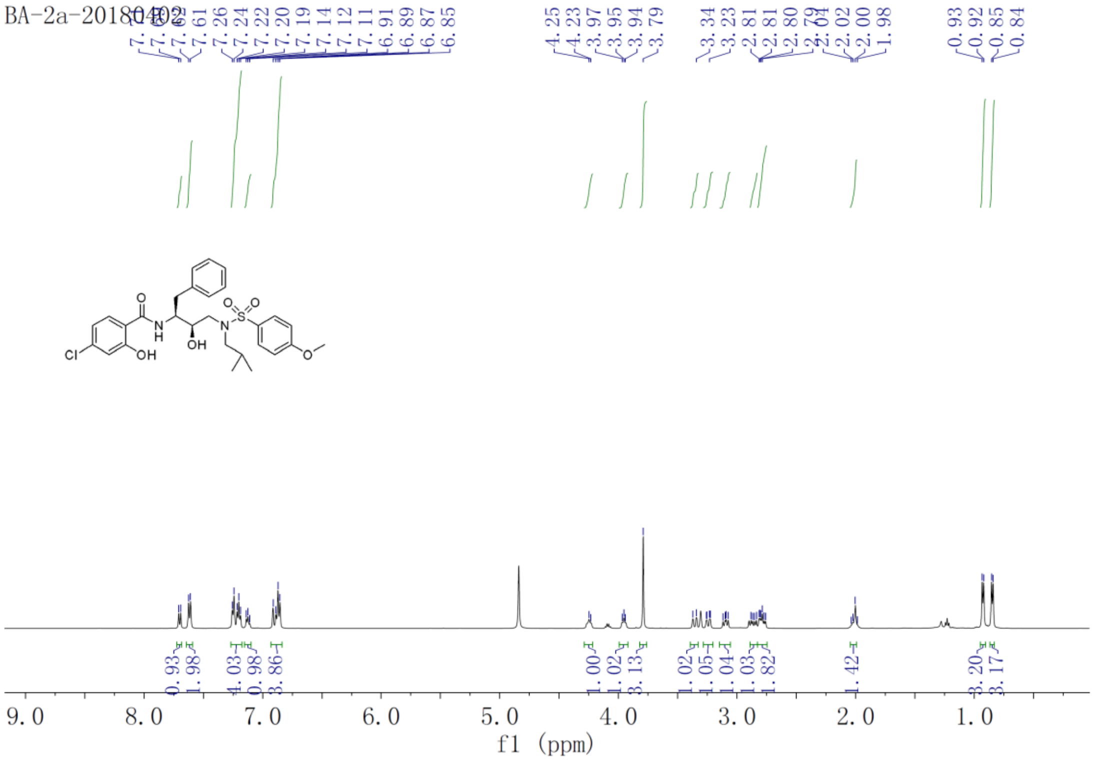

^{1}H NMR Spectrum of compound **15b**

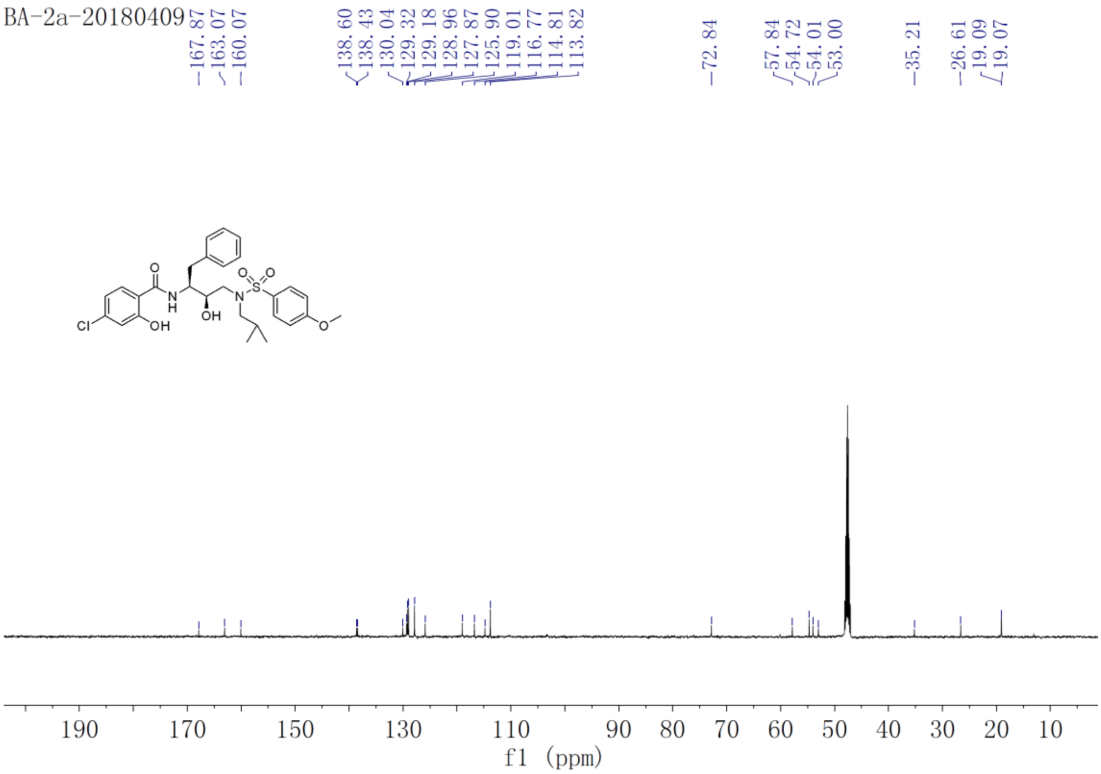

^{13}C NMR Spectrum of compound **15b**

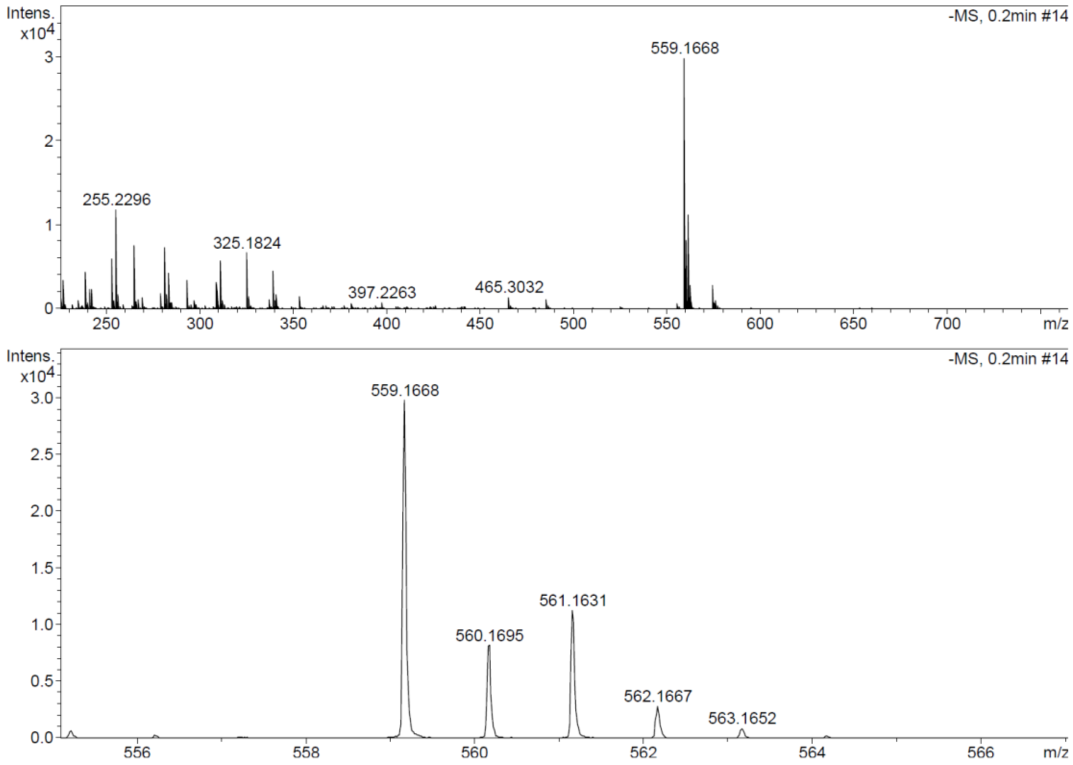

HR MS Spectrum of compound **15b**

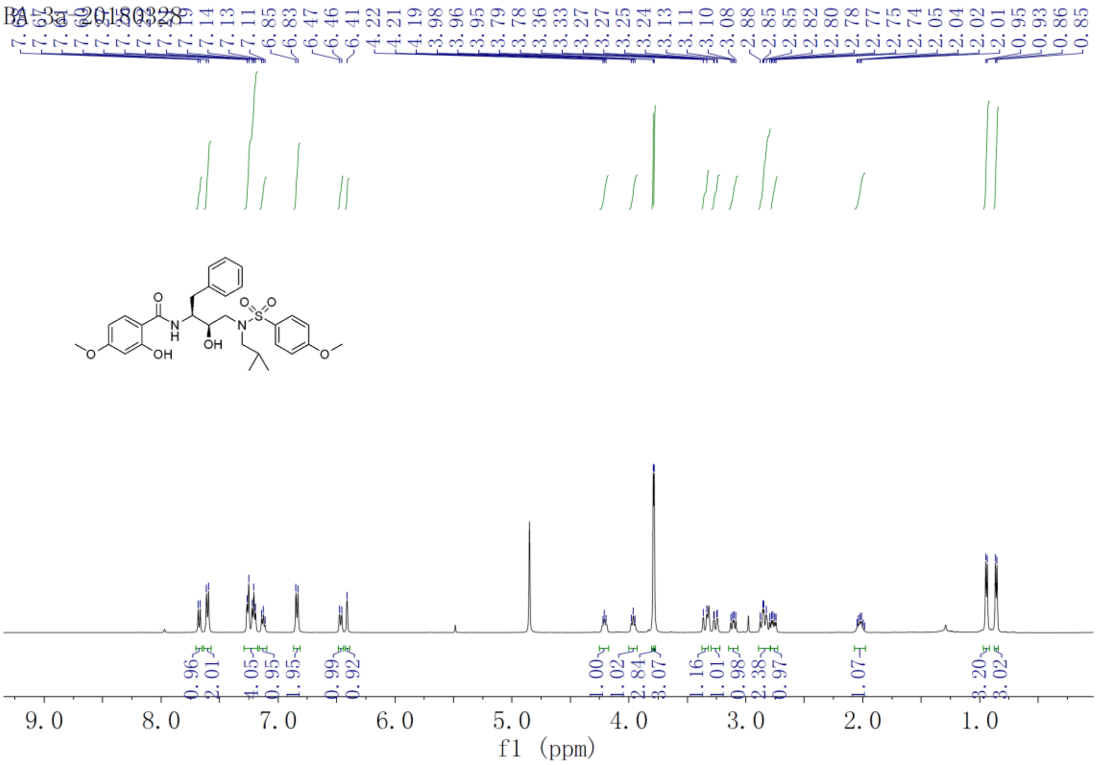

^{1}H NMR Spectrum of compound **15c**

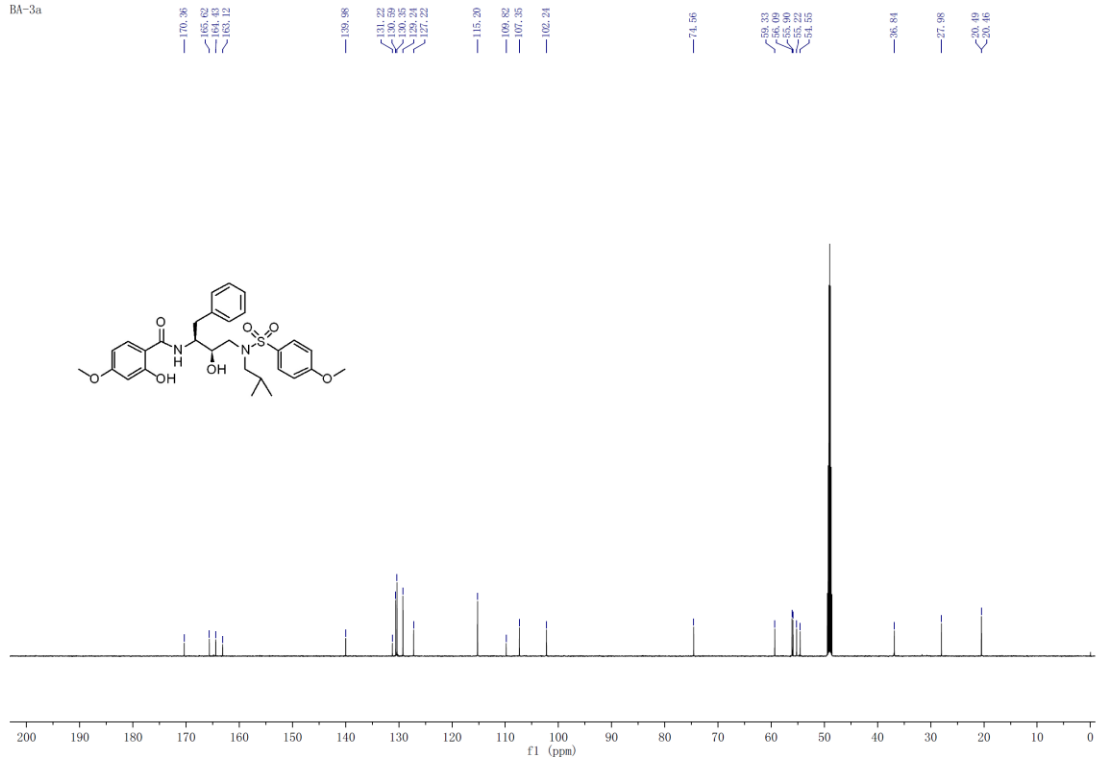

^{13}C NMR Spectrum of compound **15c**

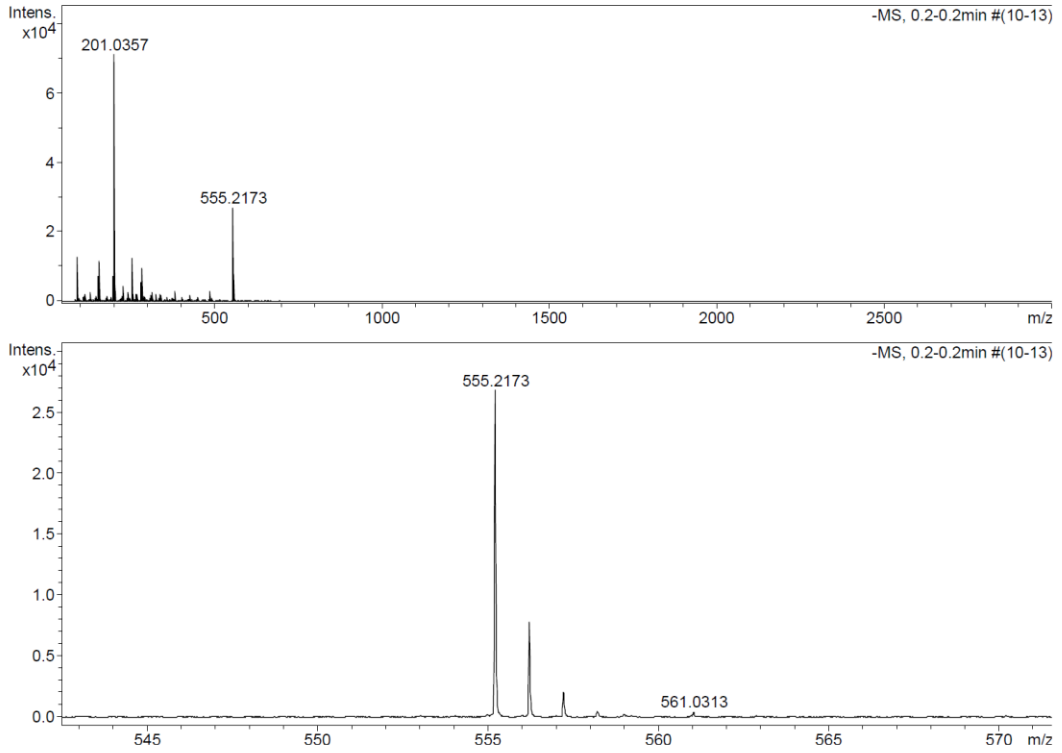

HR MS Spectrum of compound **15c**

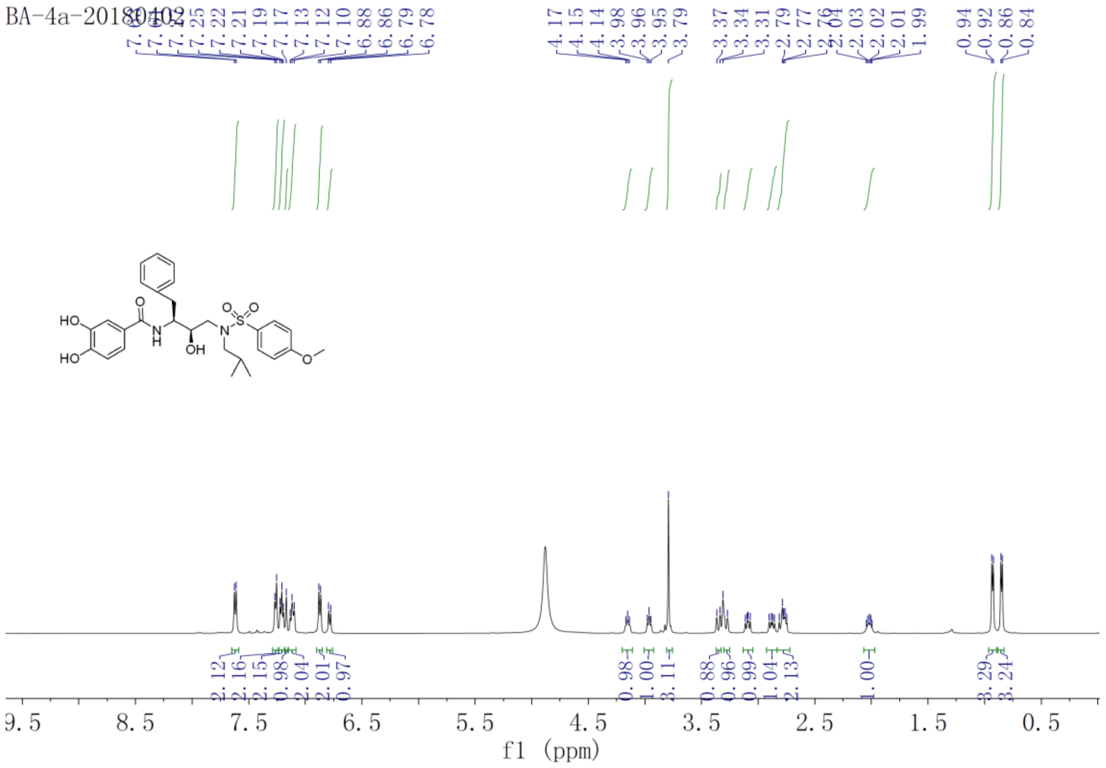

^{1}H NMR Spectrum of compound **15d**

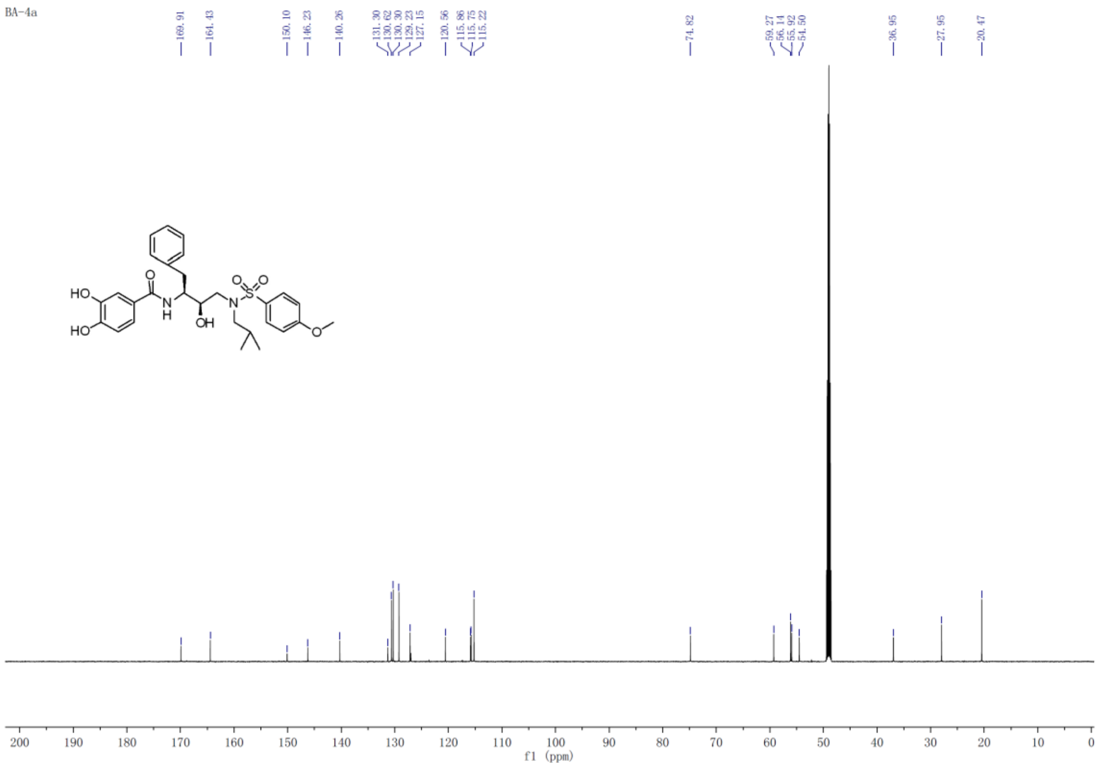

^{13}C NMR Spectrum of compound **15d**

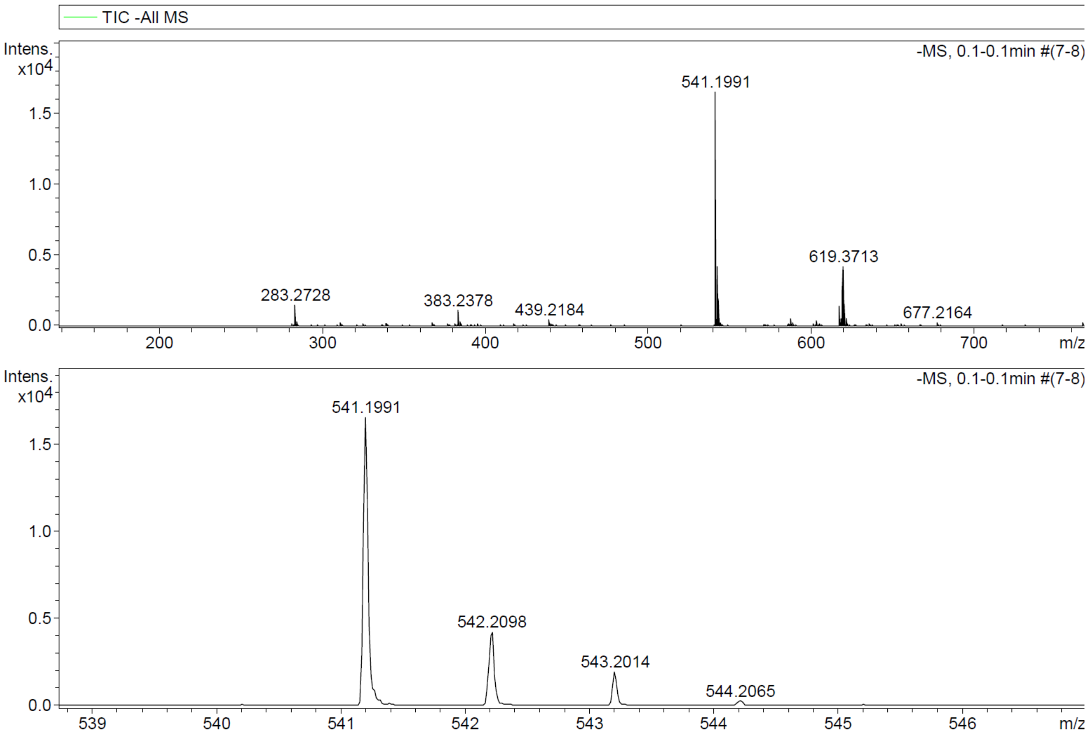

HR MS Spectrum of compound **15d**

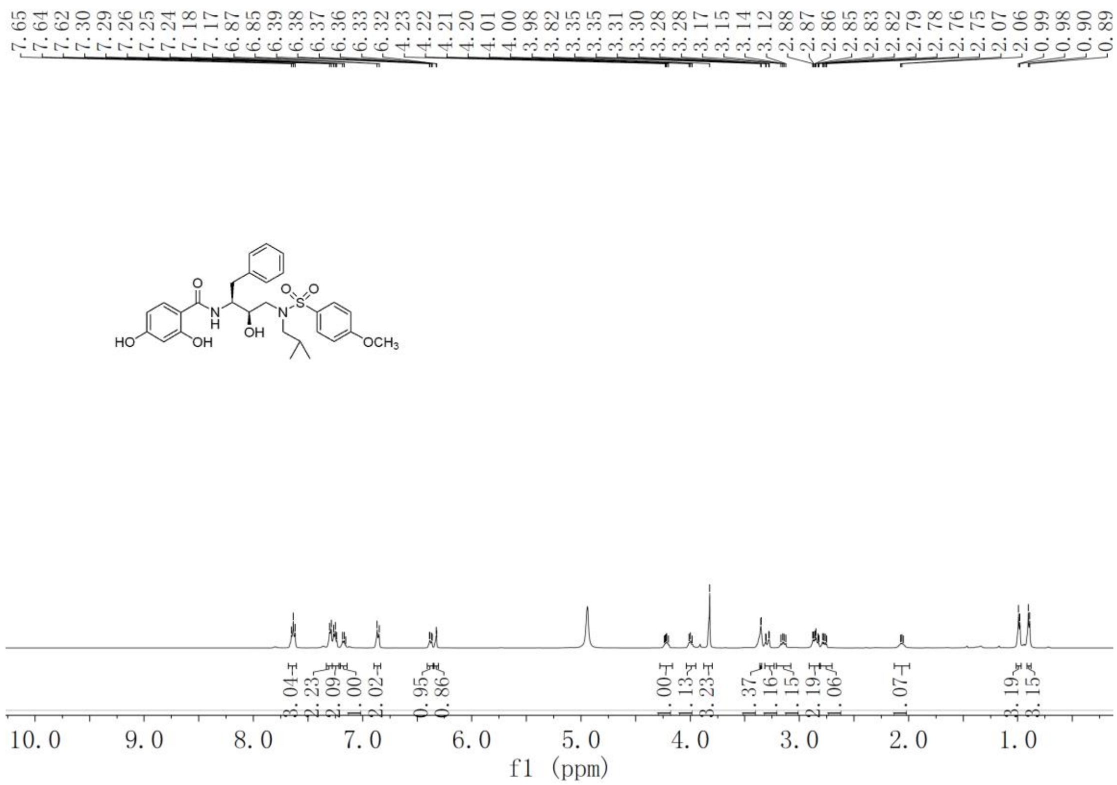

^{1}H NMR Spectrum of compound **15e**

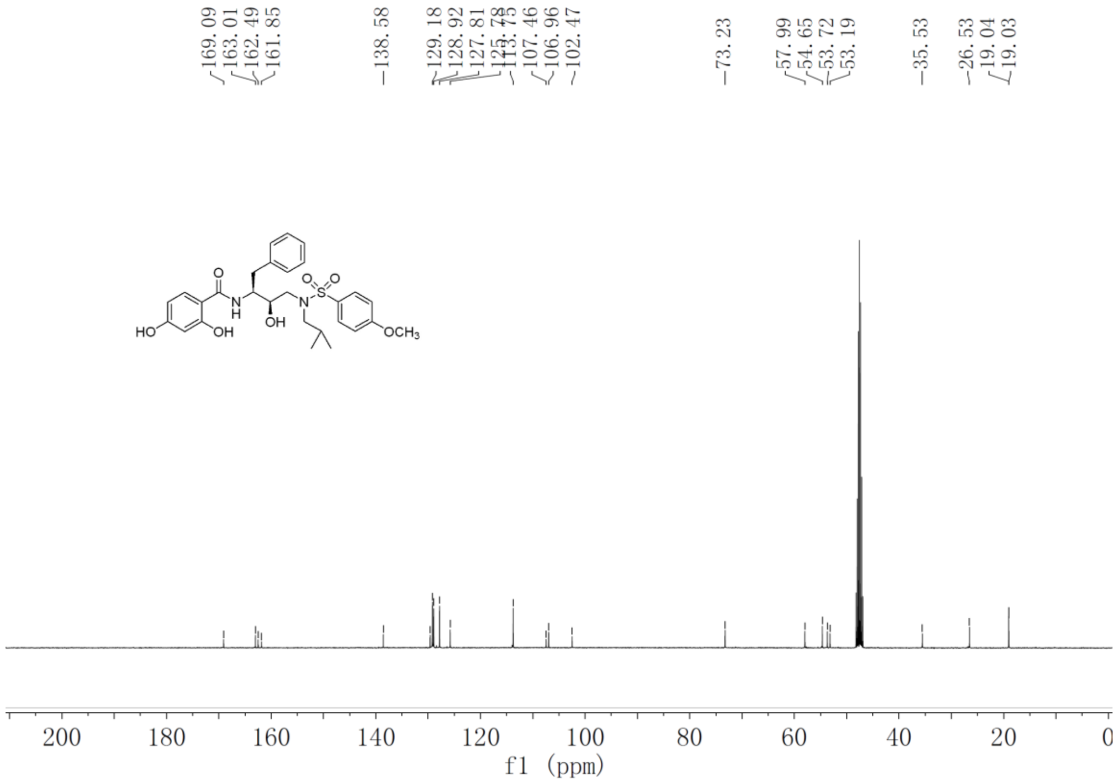

^{13}C NMR Spectrum of compound **15e**

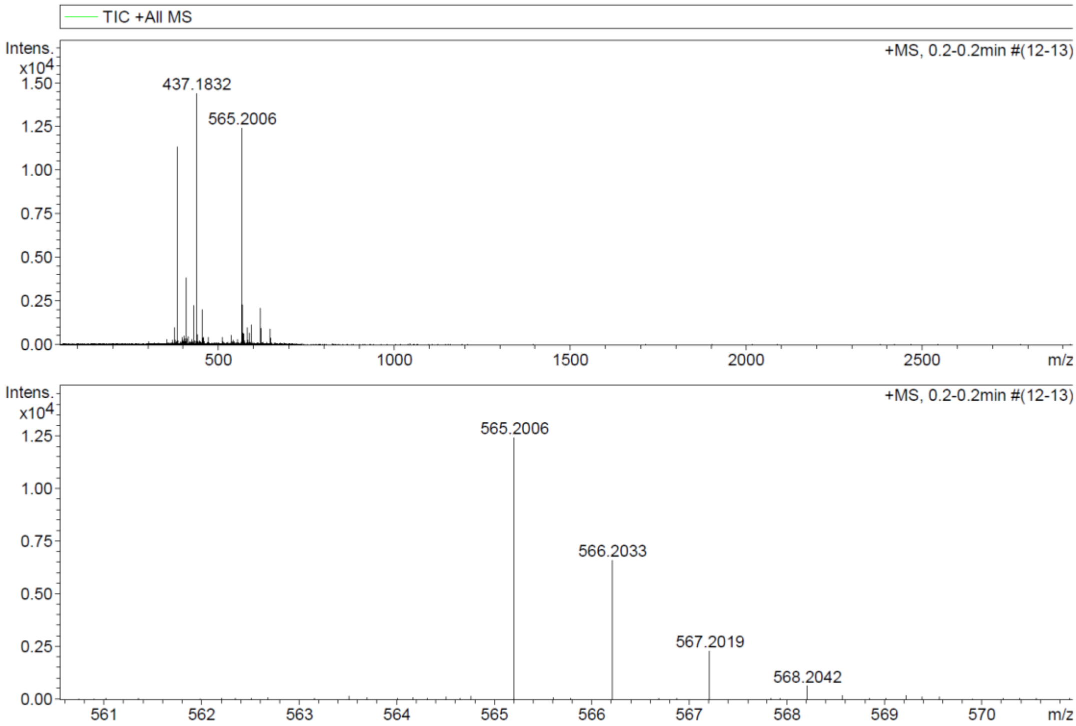

HR MS Spectrum of compound **15e**

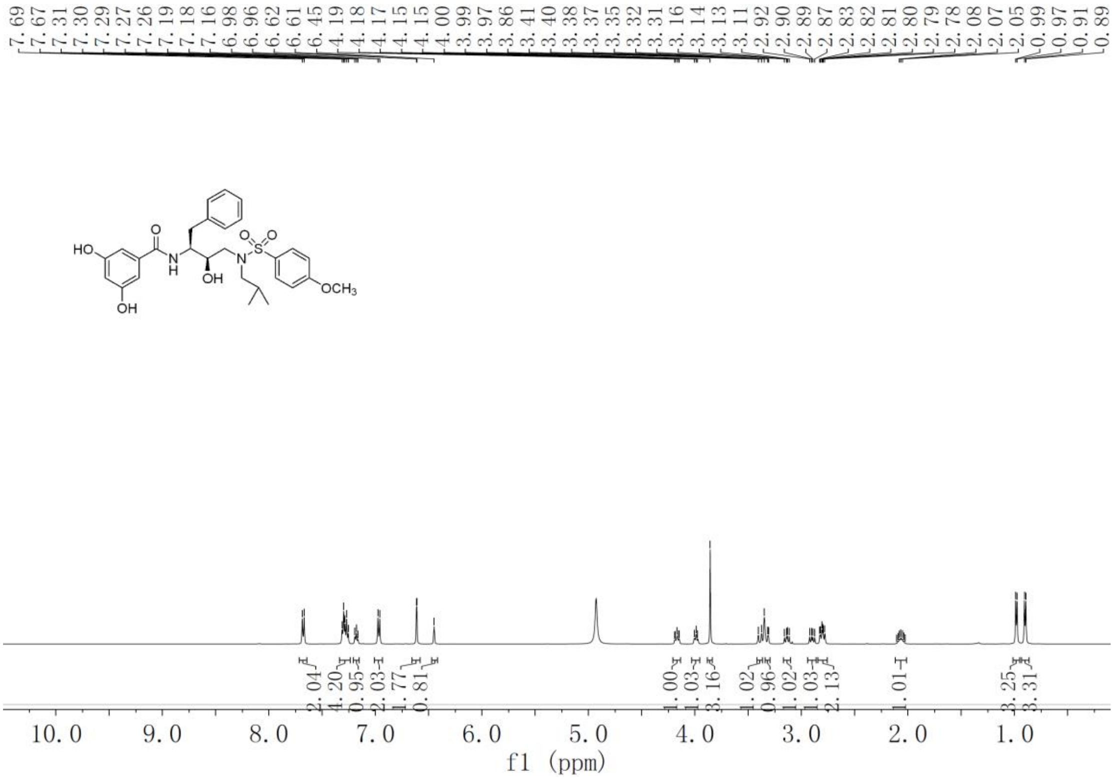

^{1}H NMR Spectrum of compound **15f**

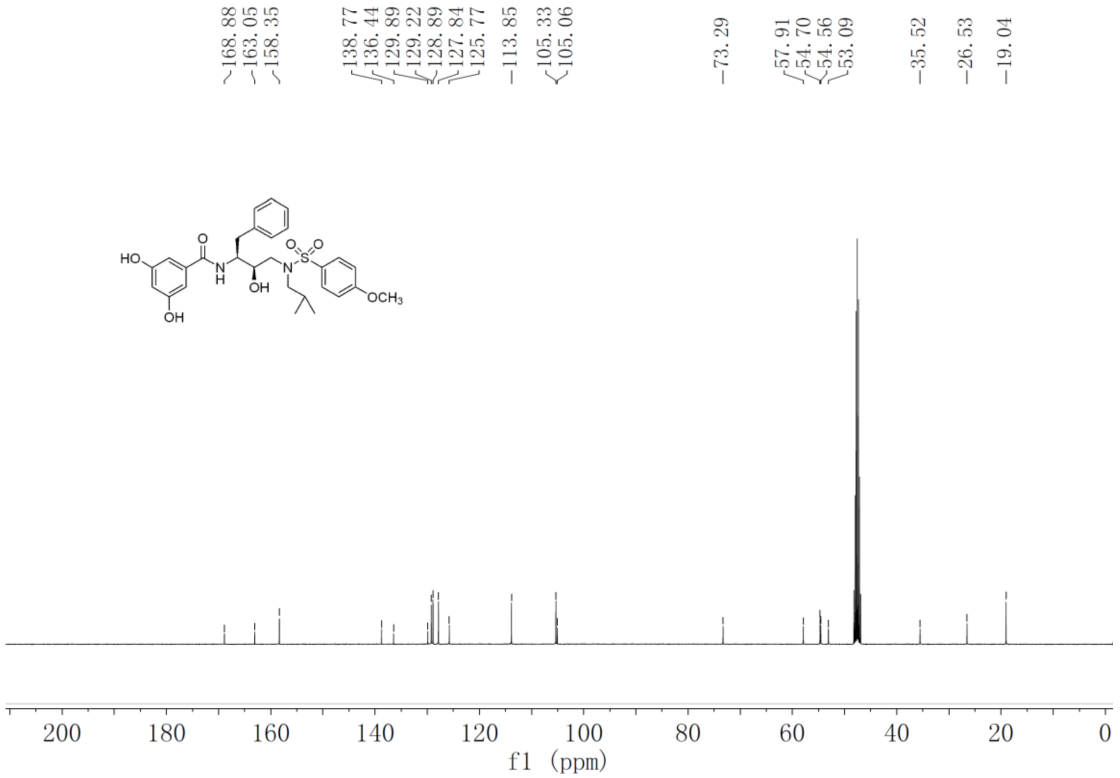

^{13}C NMR Spectrum of compound **15f**

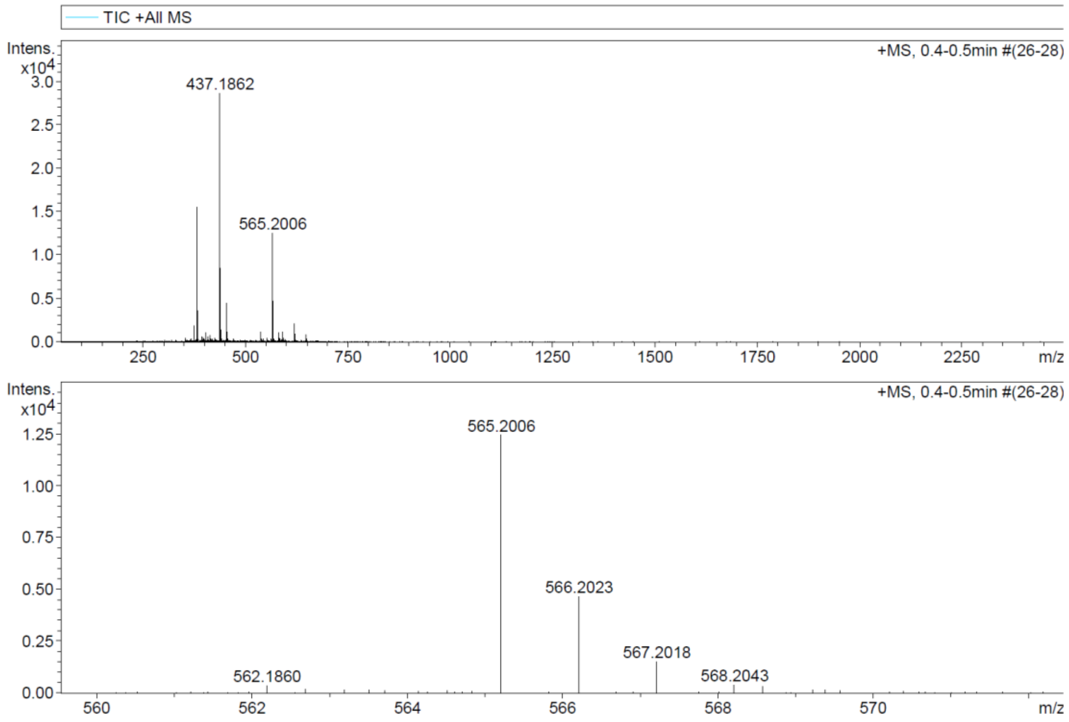

HR MS Spectrum of compound **15f**

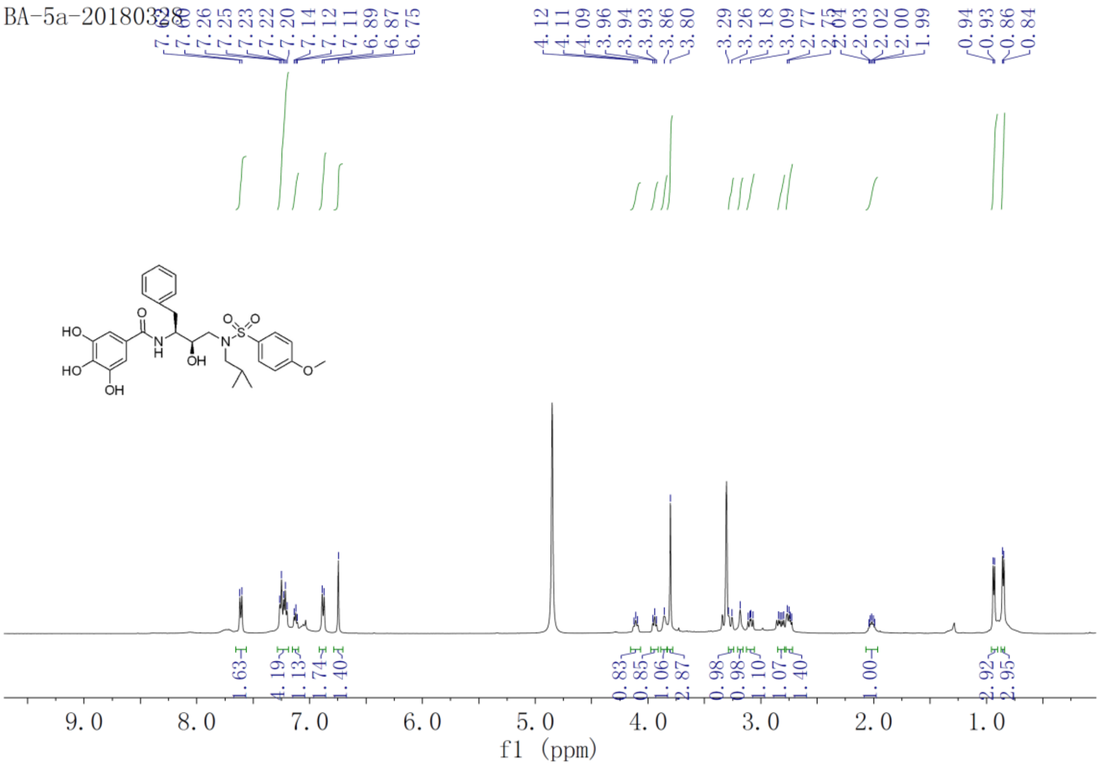

^{1}H NMR Spectrum of compound **15g**

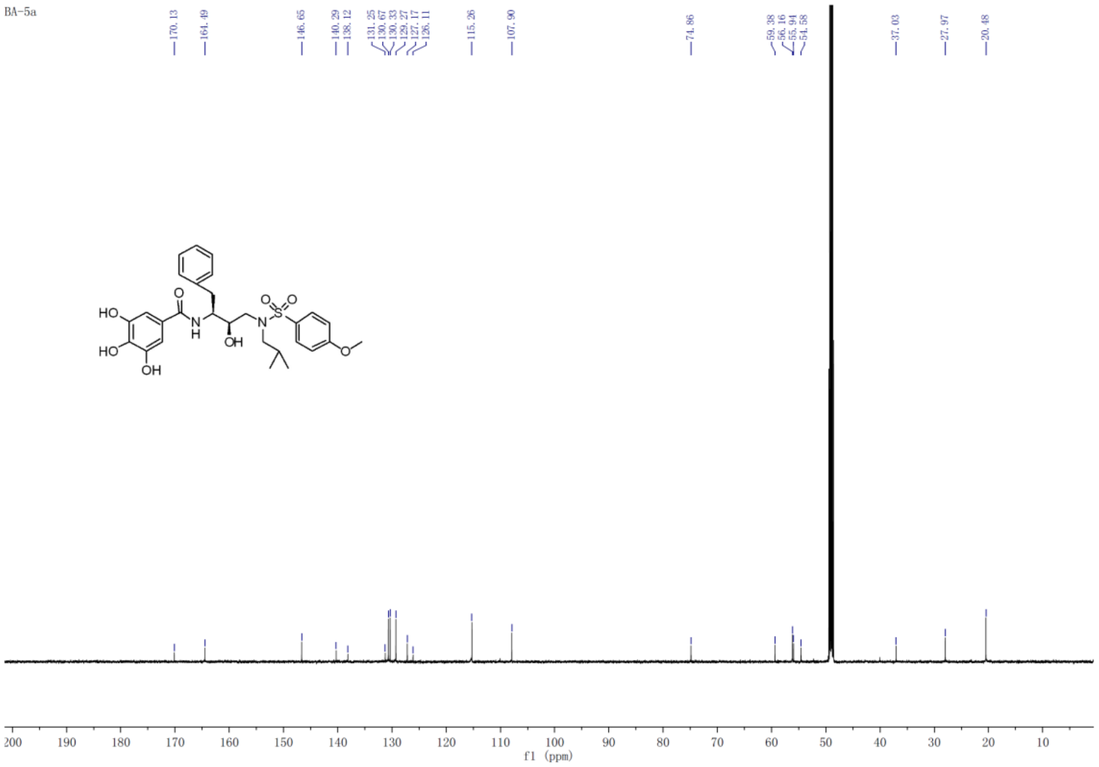

^{13}C NMR Spectrum of compound **15g**

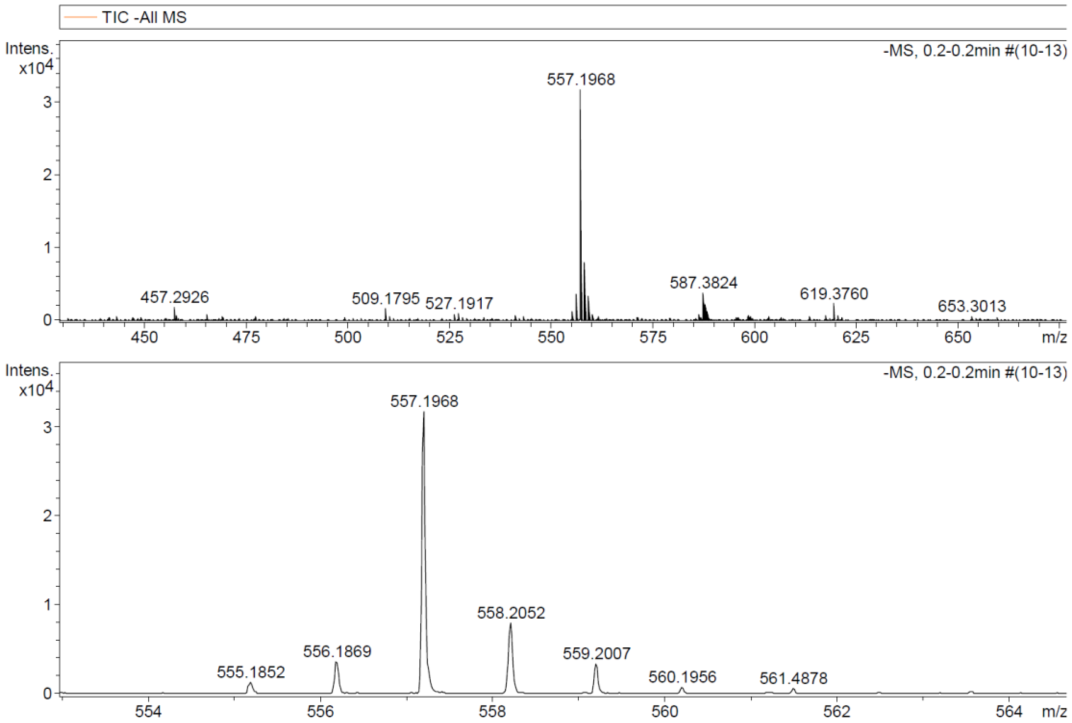

HR MS Spectrum of compound **15g**

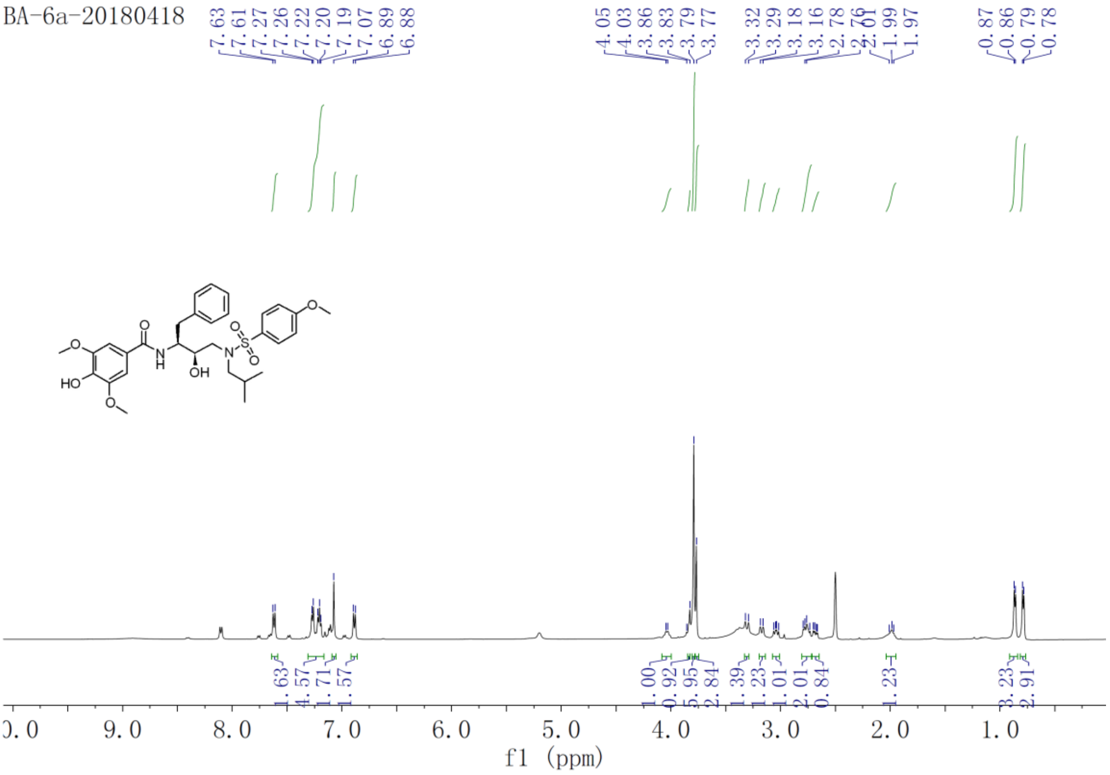

^{1}H NMR Spectrum of compound **15h**

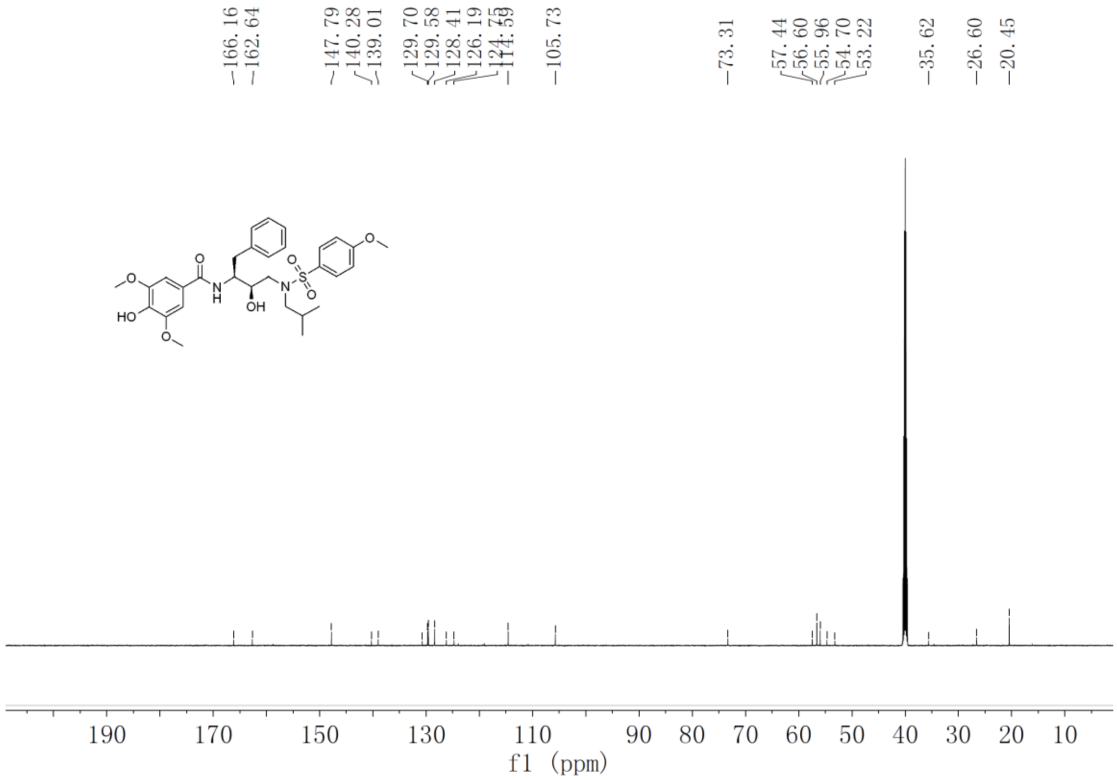

^{13}C NMR Spectrum of compound **15h**

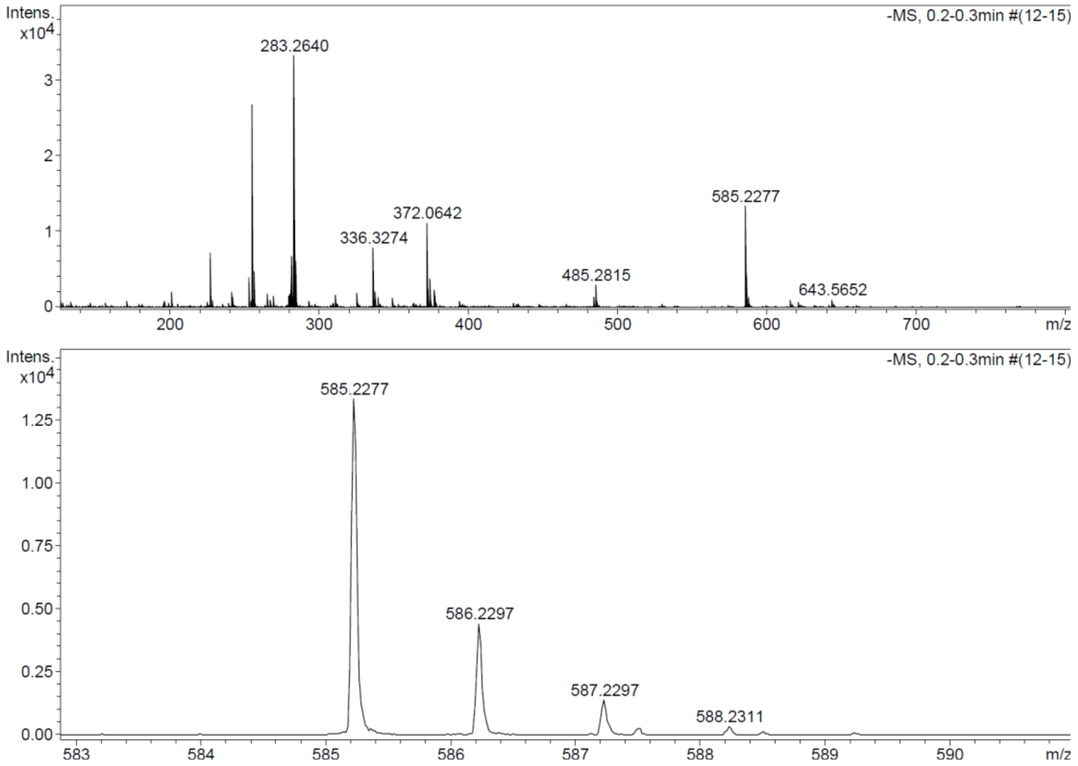

HR MS Spectrum of compound **15h**

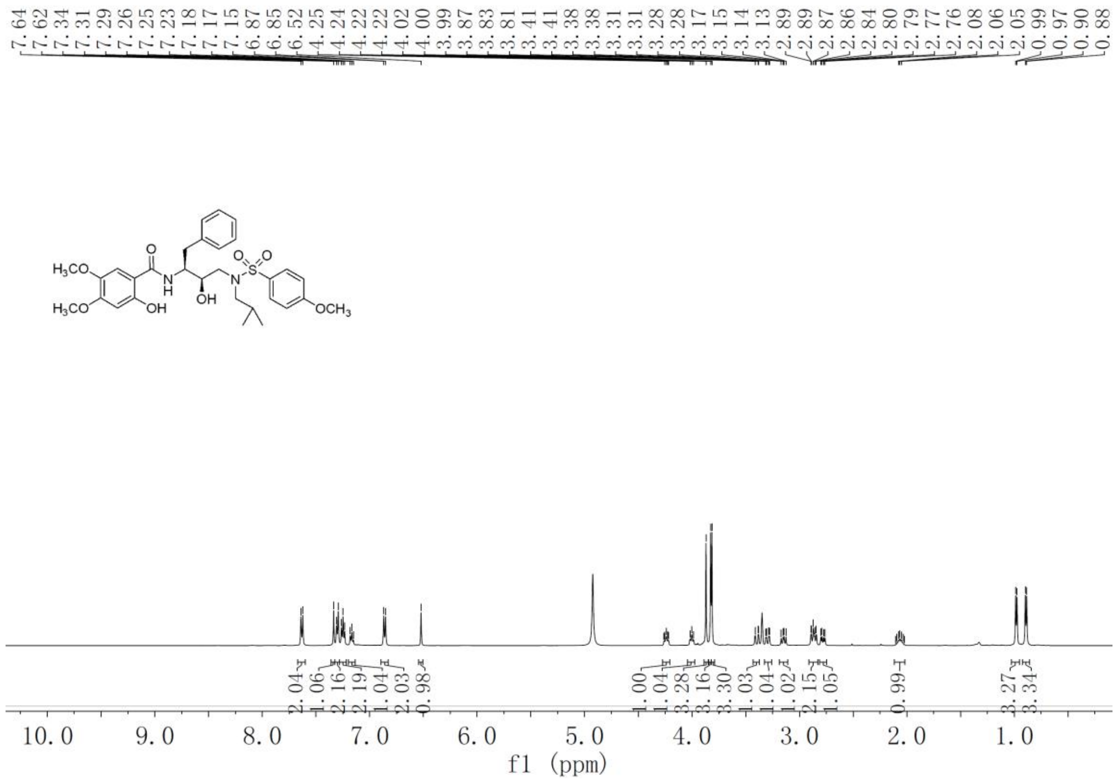

^{1}H NMR Spectrum of compound **15i**

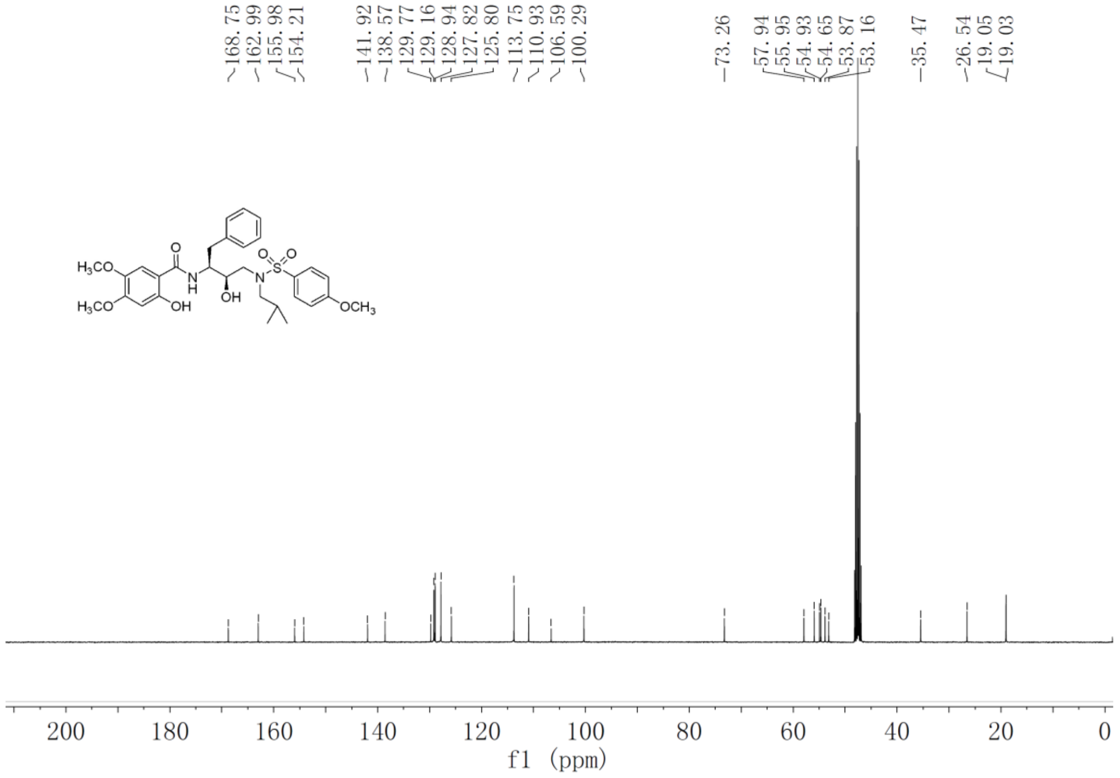

^{13}C NMR Spectrum of compound **15i**

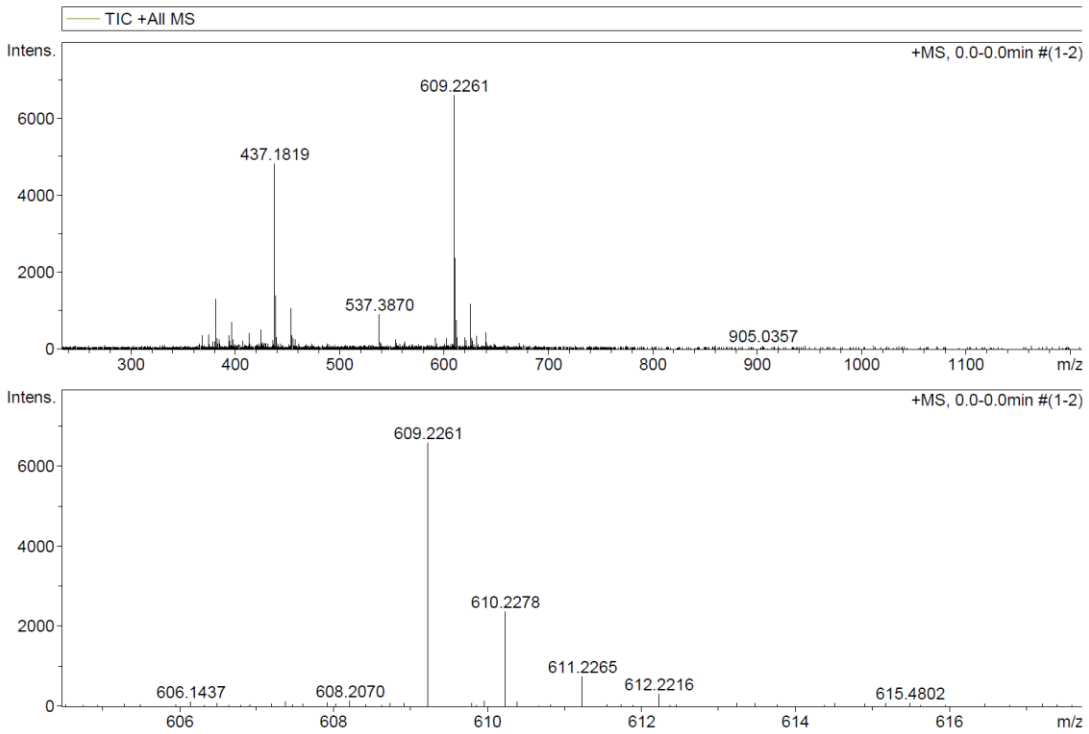

HR MS Spectrum of compound **15i**

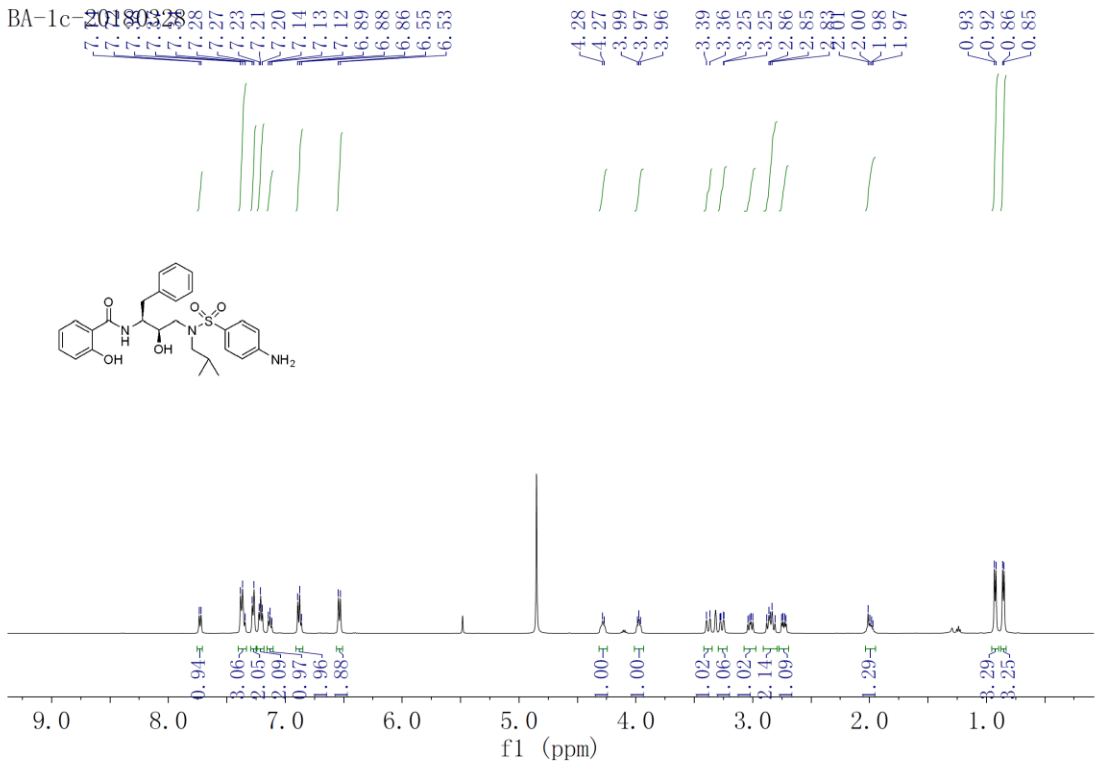

^{1}H NMR Spectrum of compound **16a**

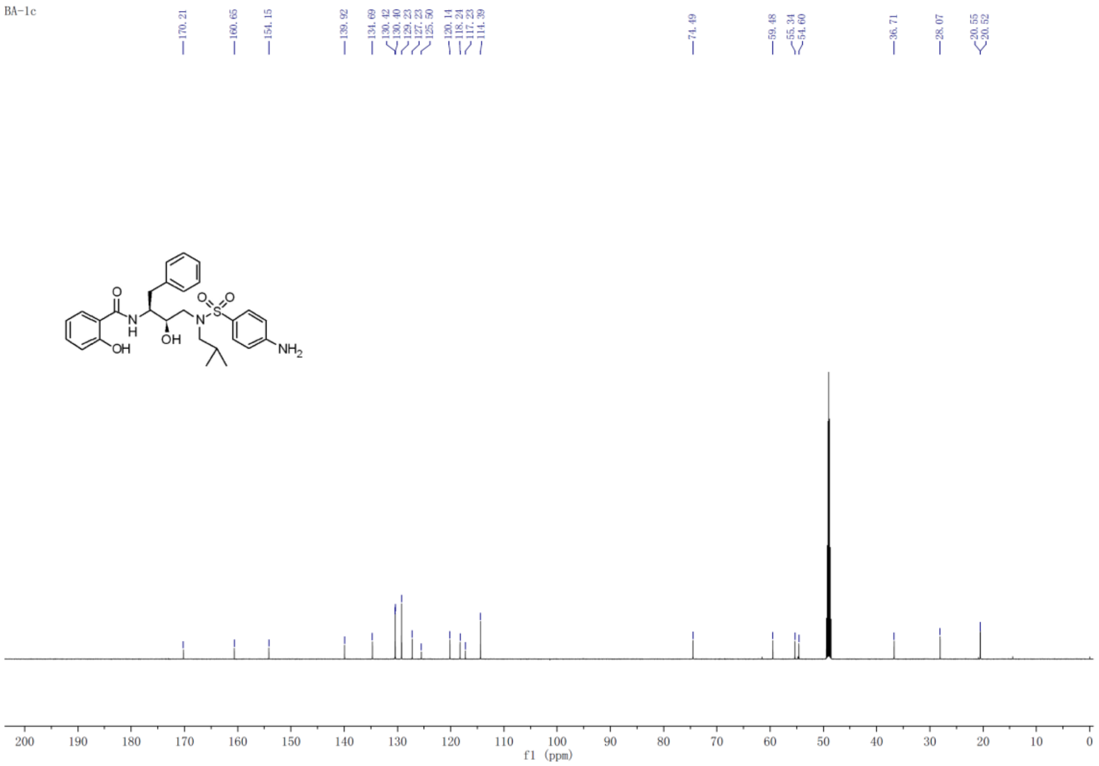

^{13}C NMR Spectrum of compound **16a**

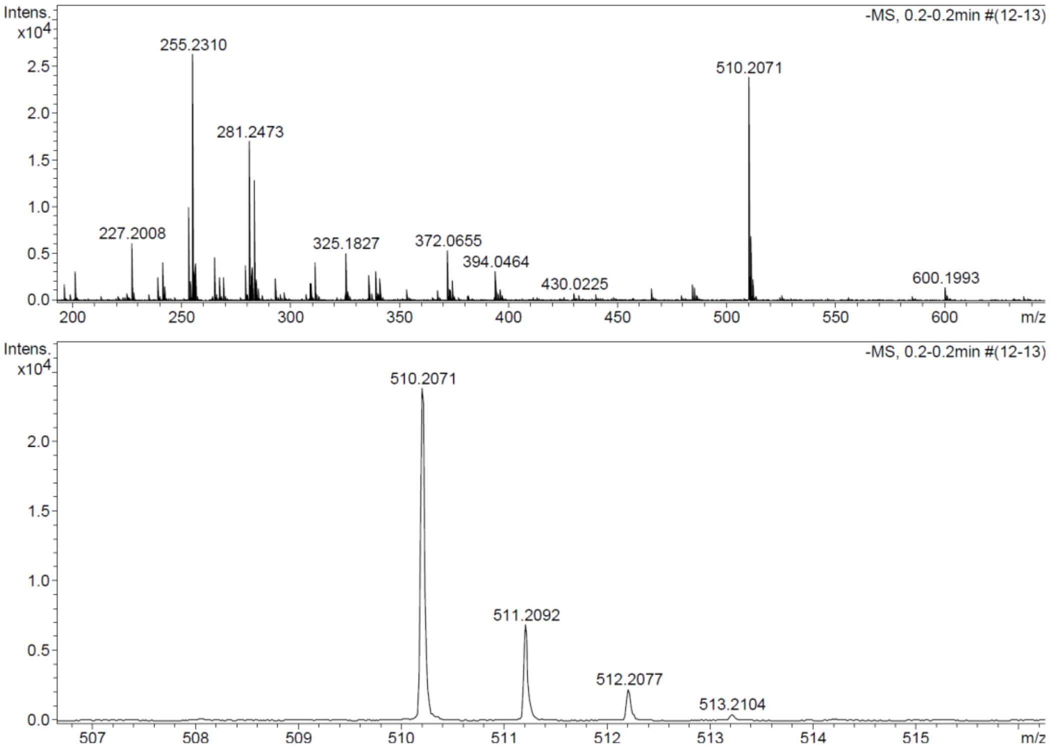

HR MS Spectrum of compound **16a**

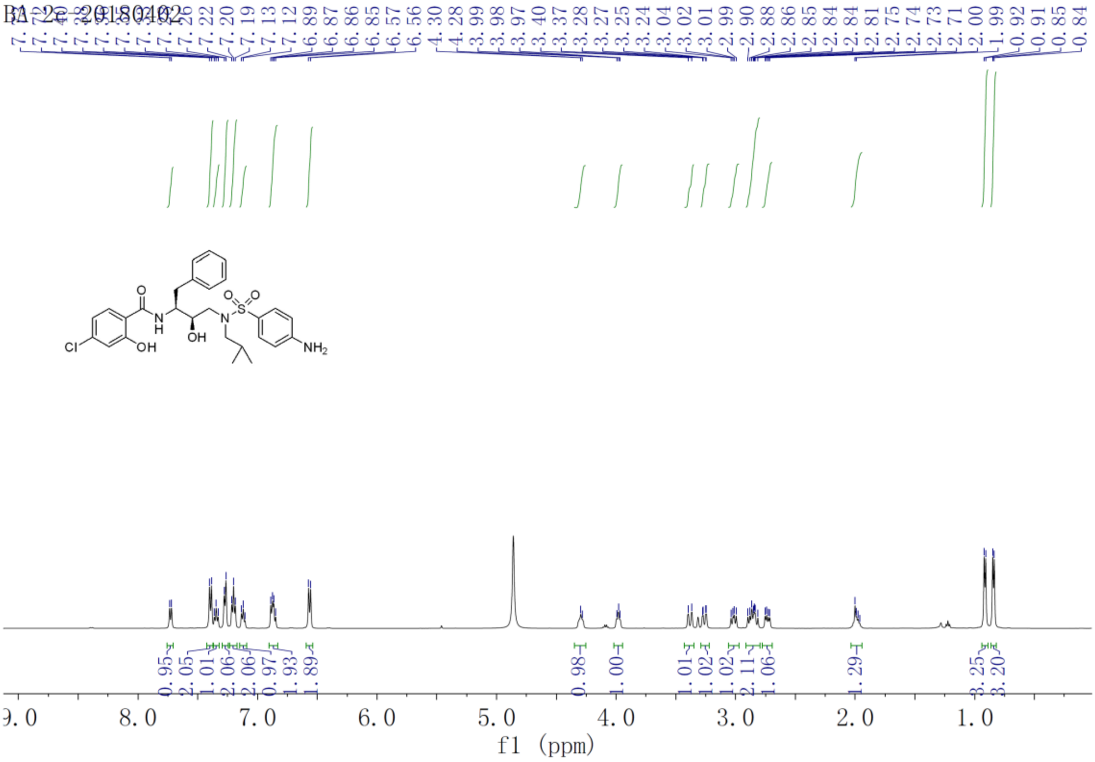

^{1}H NMR Spectrum of compound **16b**

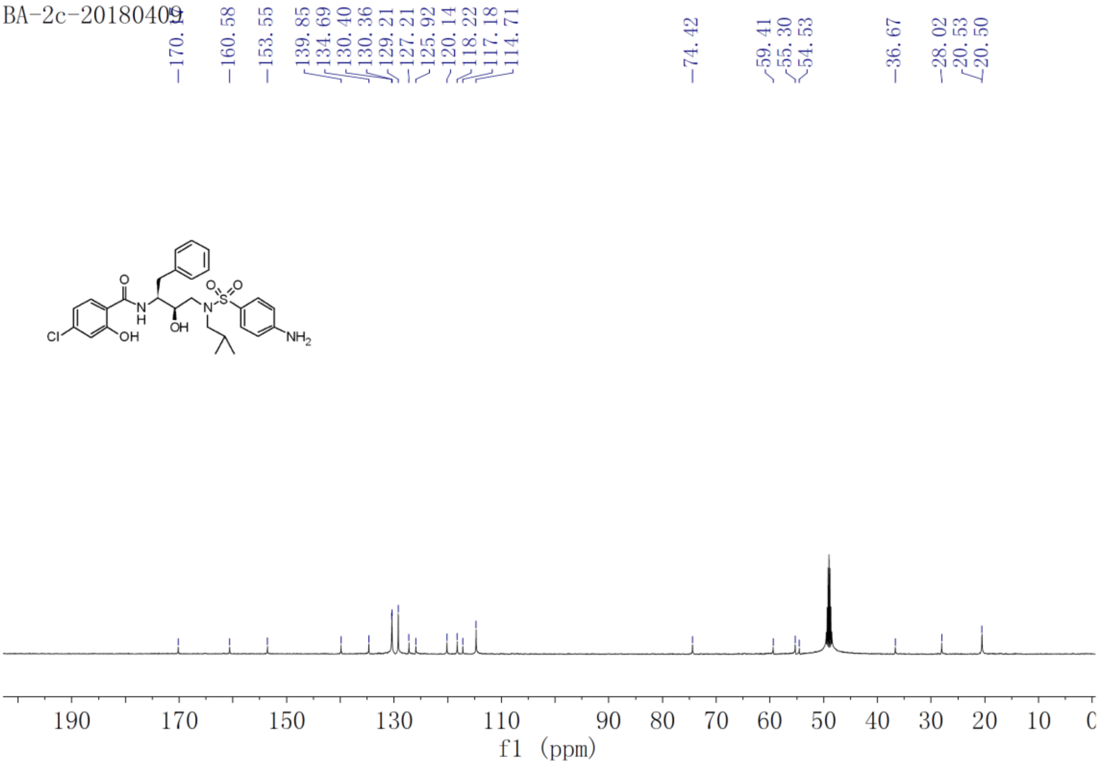

^{13}C NMR Spectrum of compound **16b**

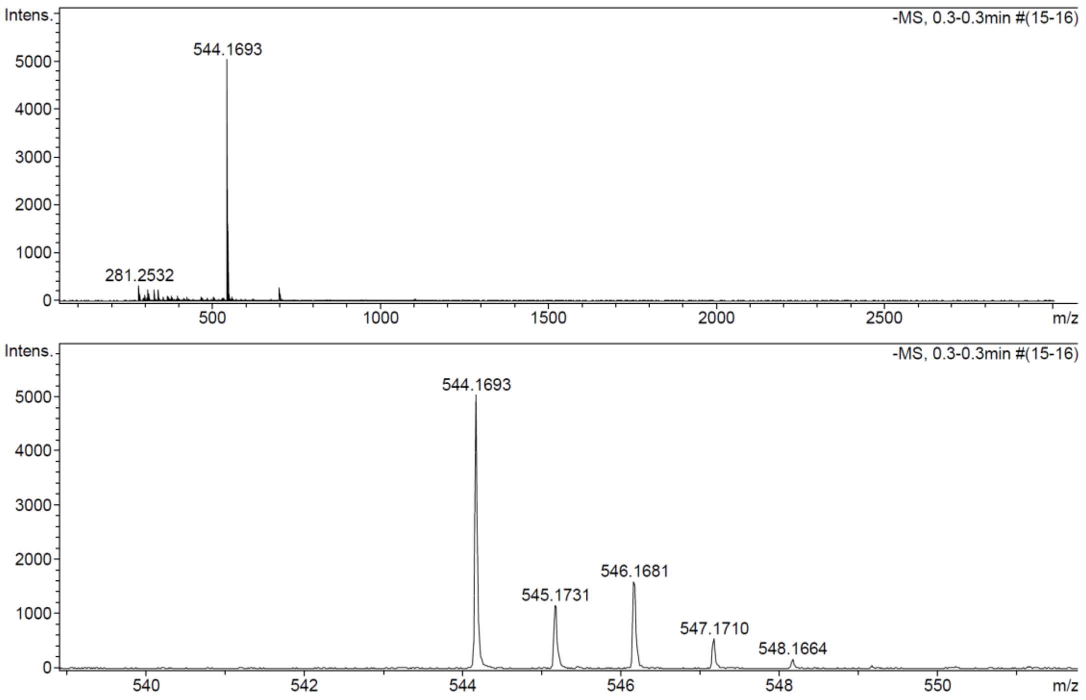

HR MS Spectrum of compound **16b**

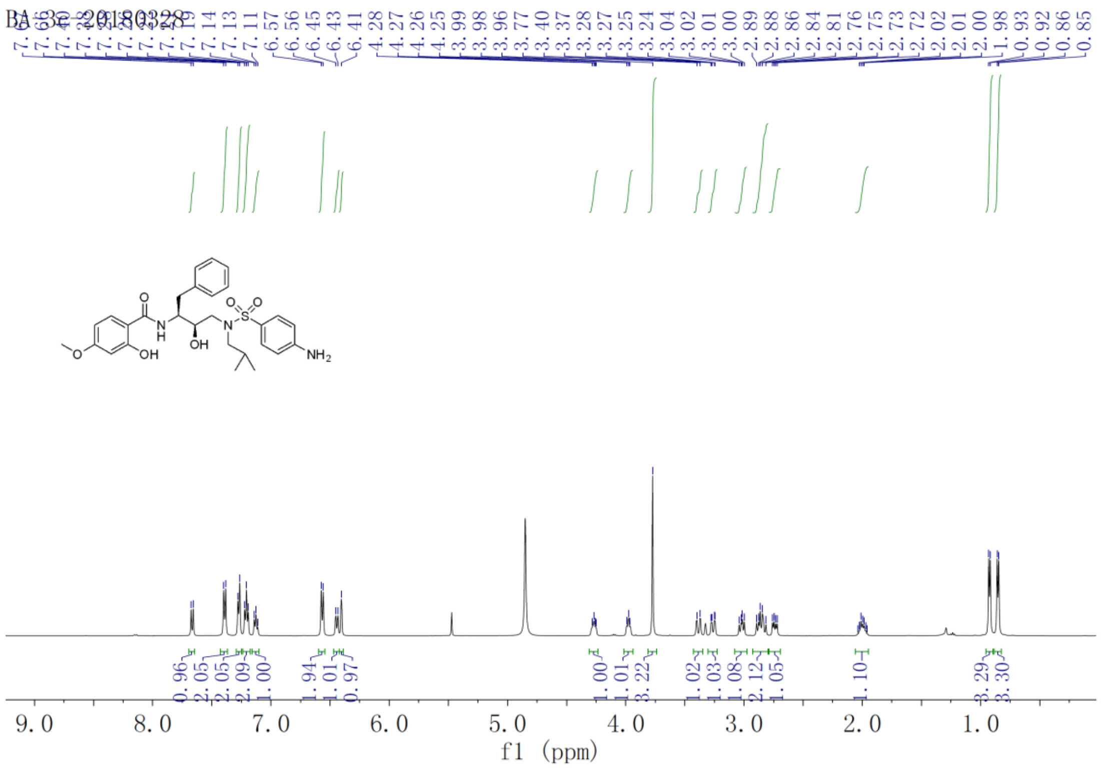

^{1}H NMR Spectrum of compound **16c**

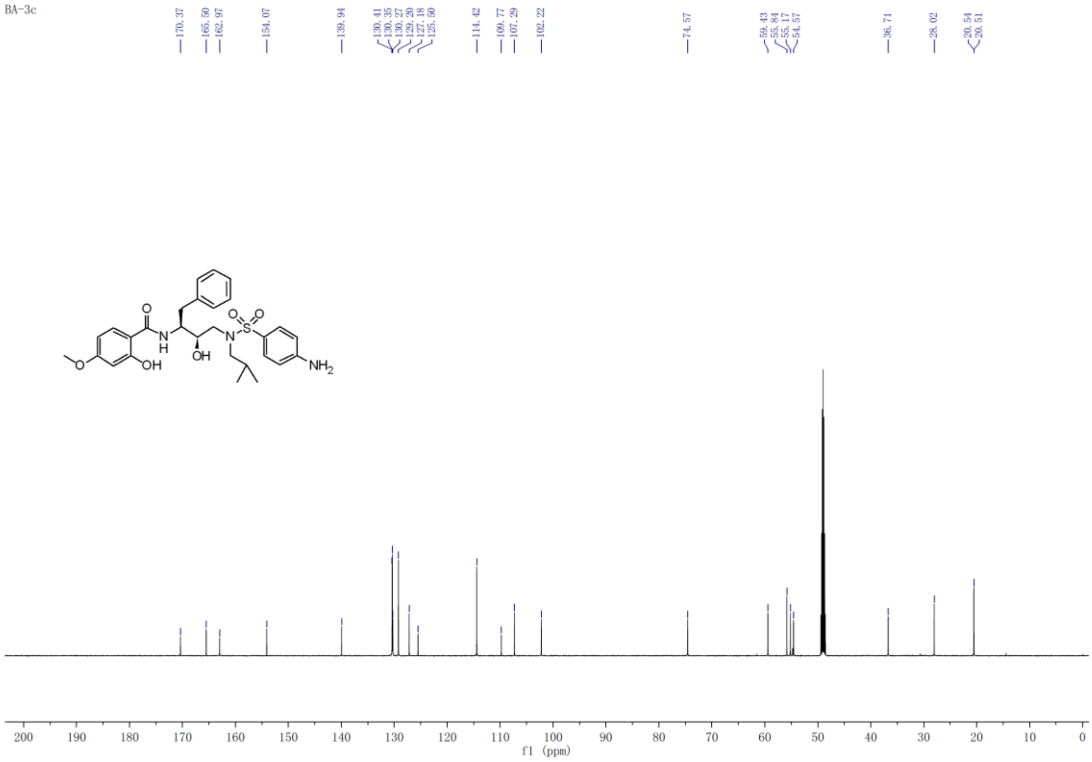

^{13}C NMR Spectrum of compound **16c**

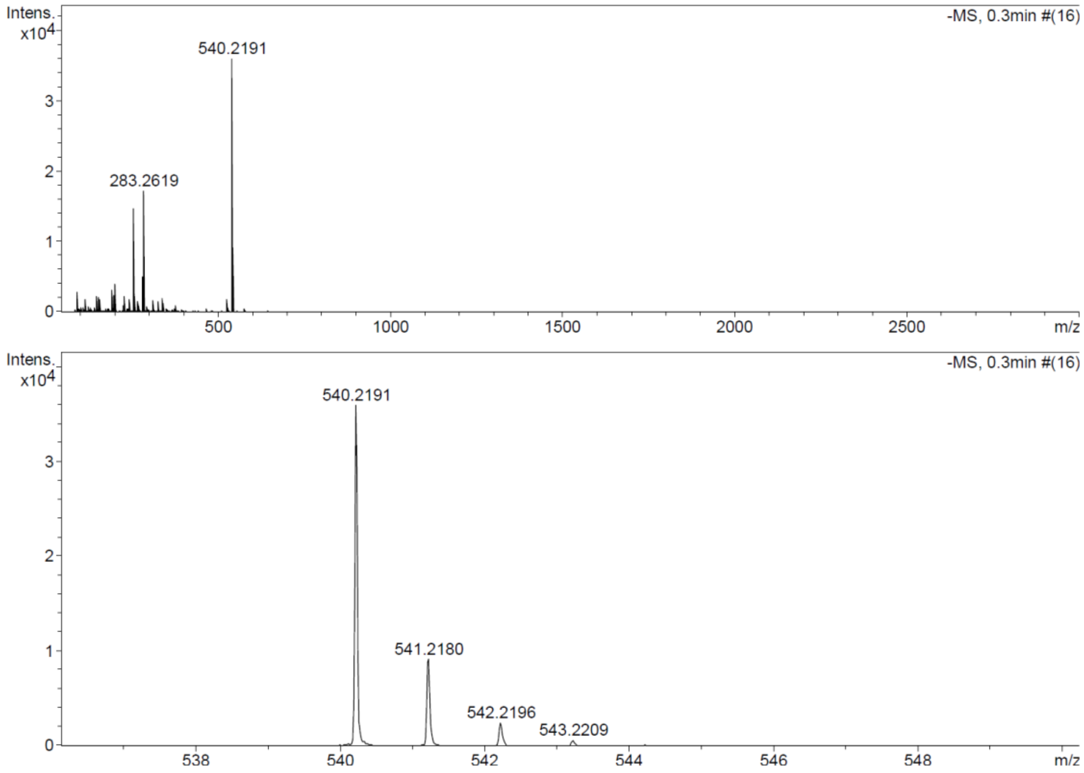

HR MS Spectrum of compound **16c**

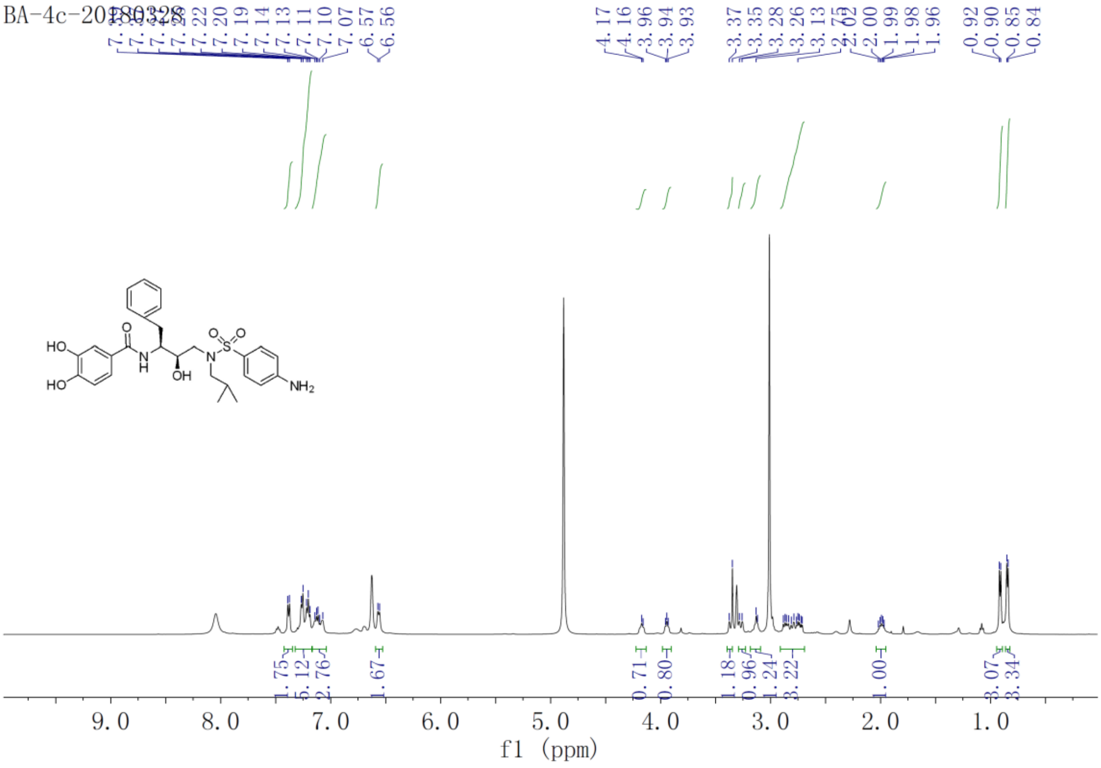

^{1}H NMR Spectrum of compound **16d**

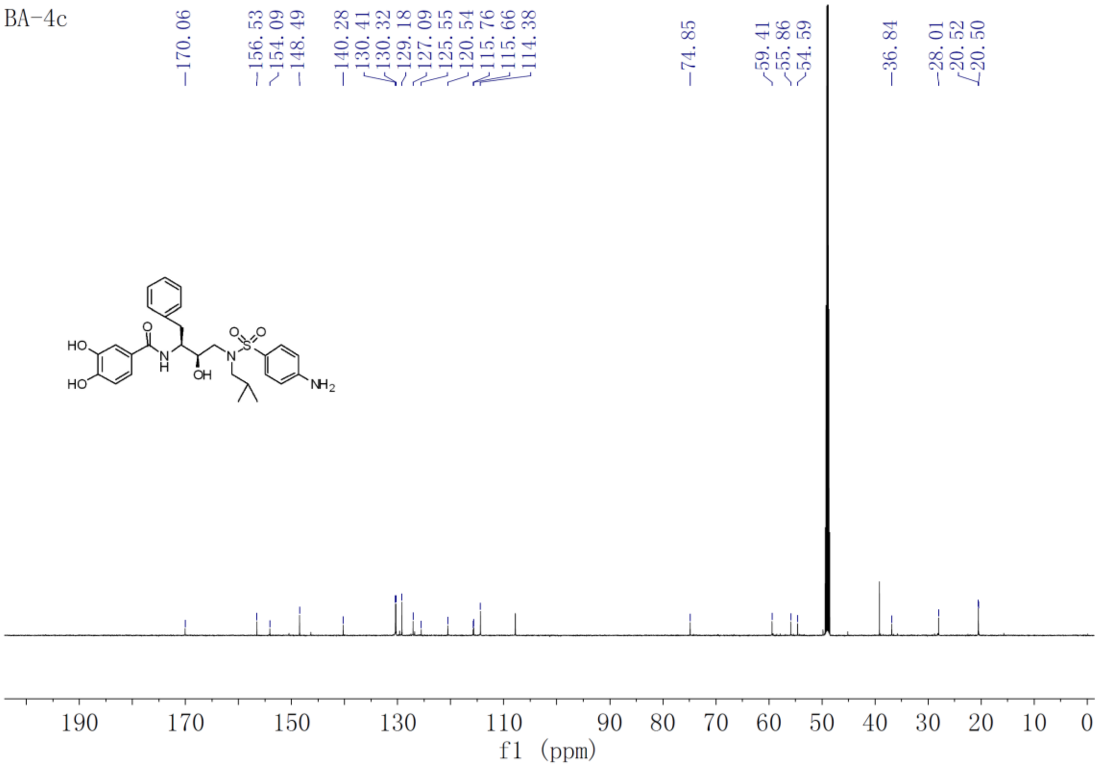

^{13}C NMR Spectrum of compound **16d**

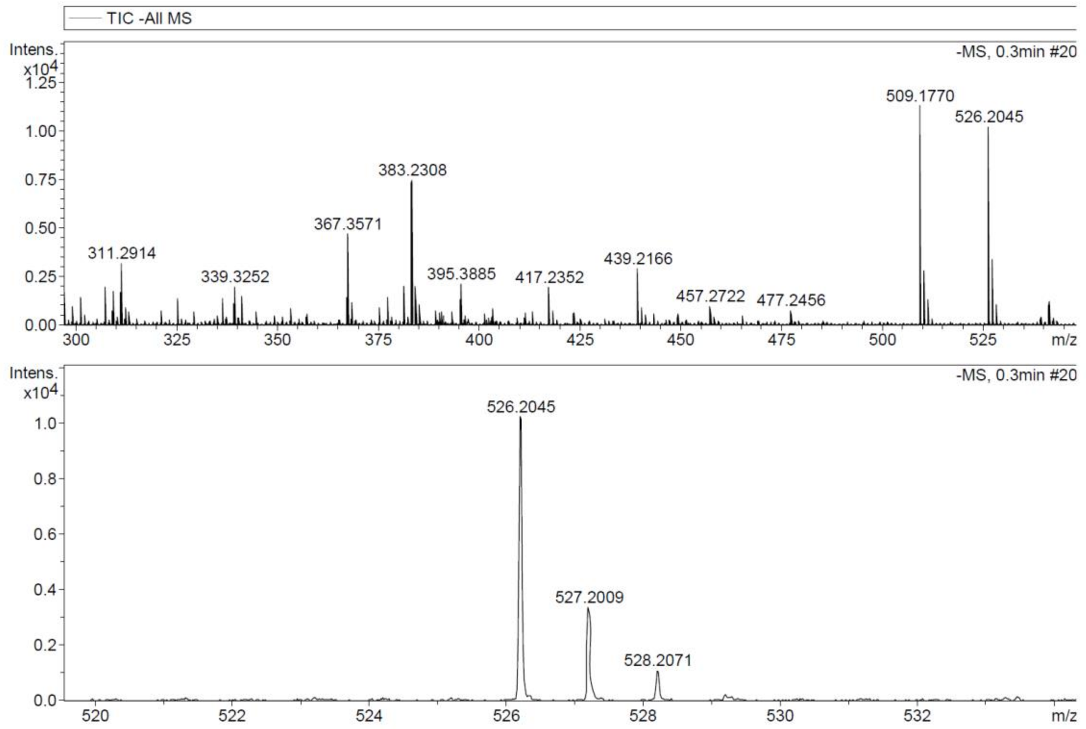

HR MS Spectrum of compound **16d**

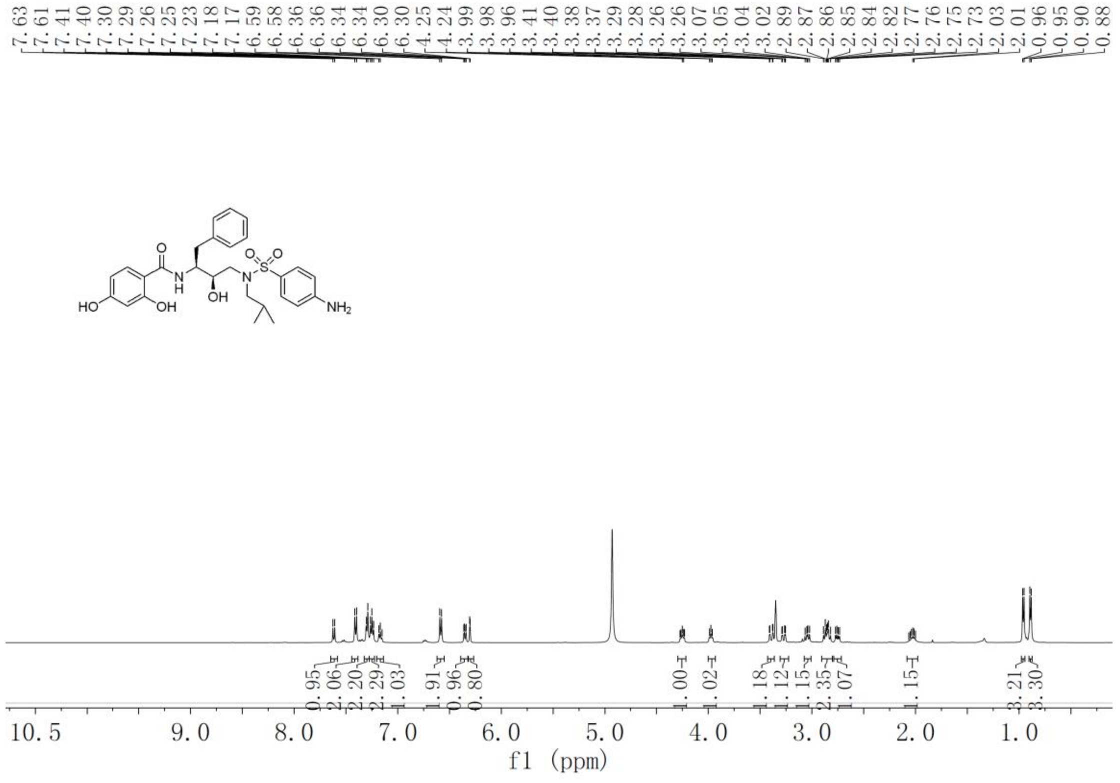

^{1}H NMR Spectrum of compound **16e**

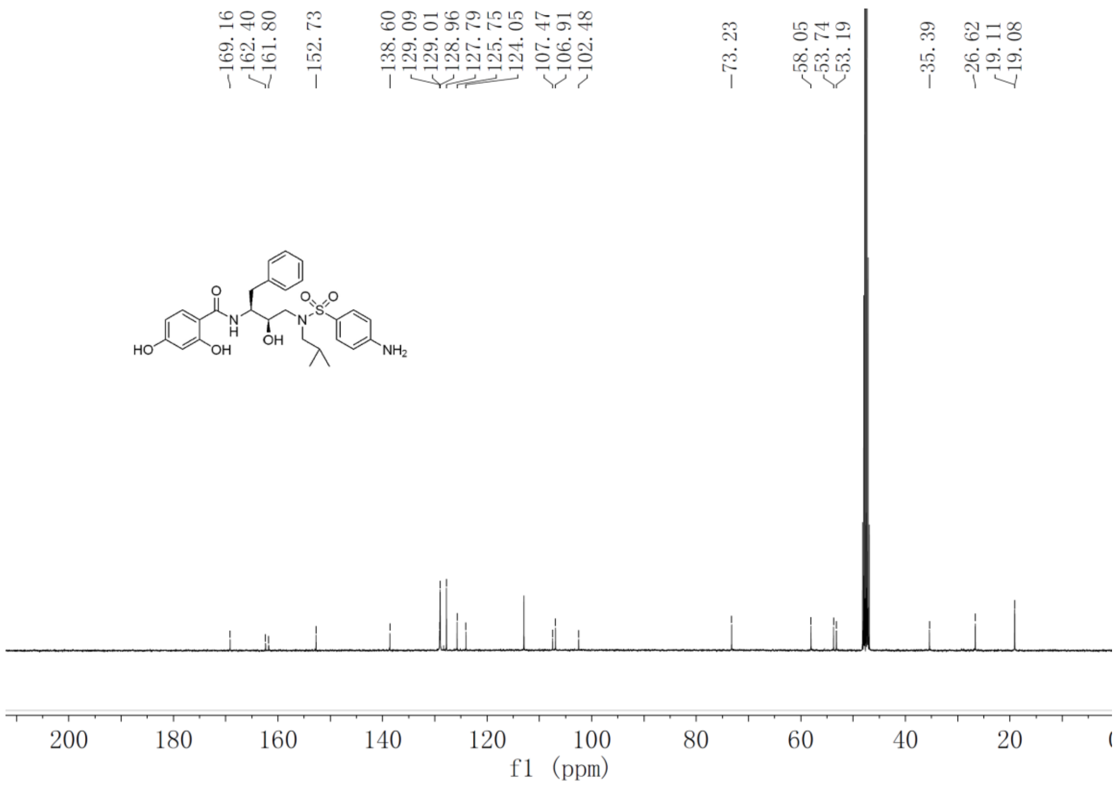

^{13}C NMR Spectrum of compound **16e**

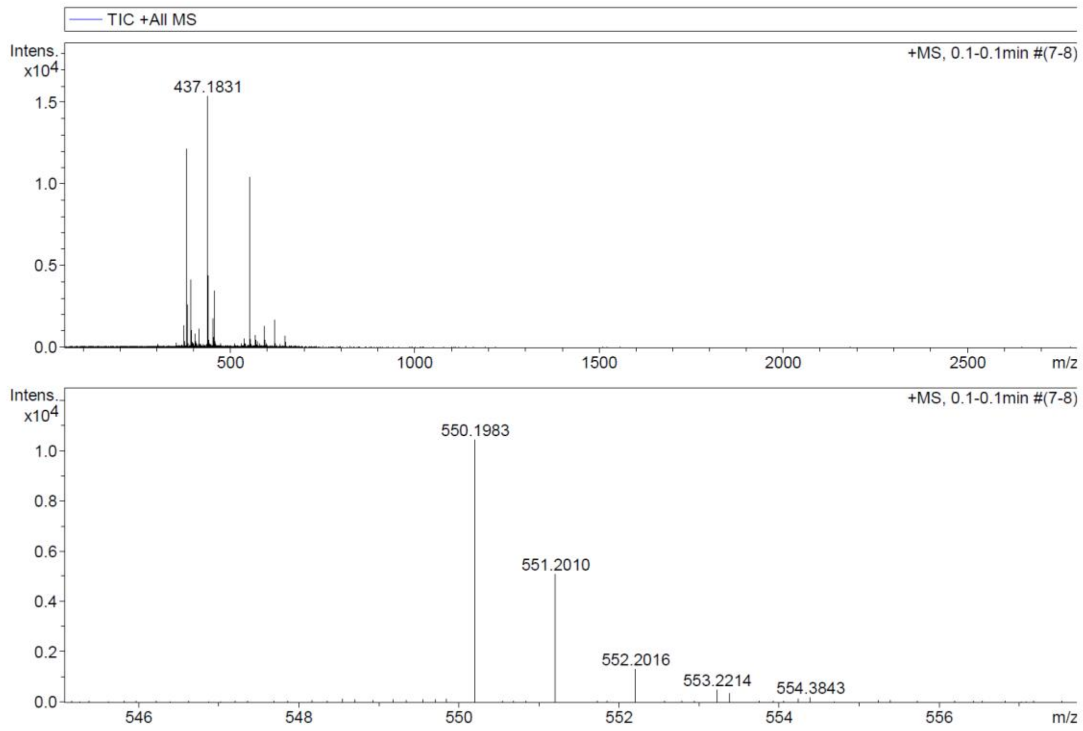

HR MS Spectrum of compound **16e**

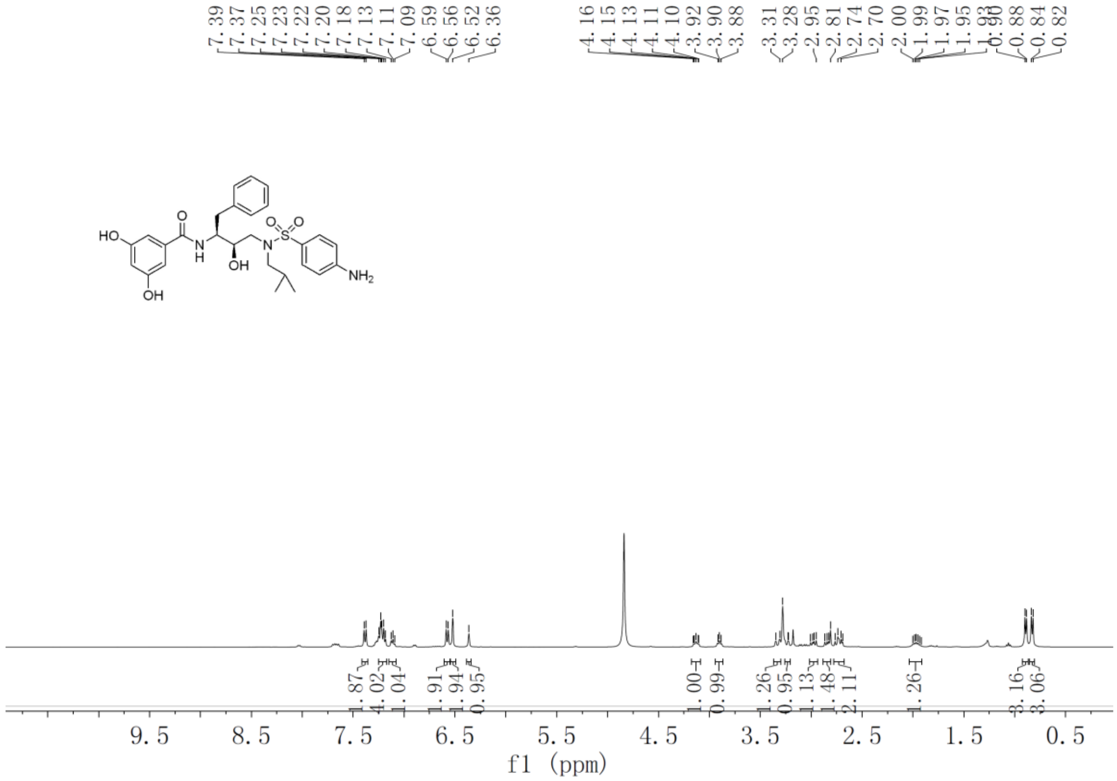

^{1}H NMR Spectrum of compound **16f**

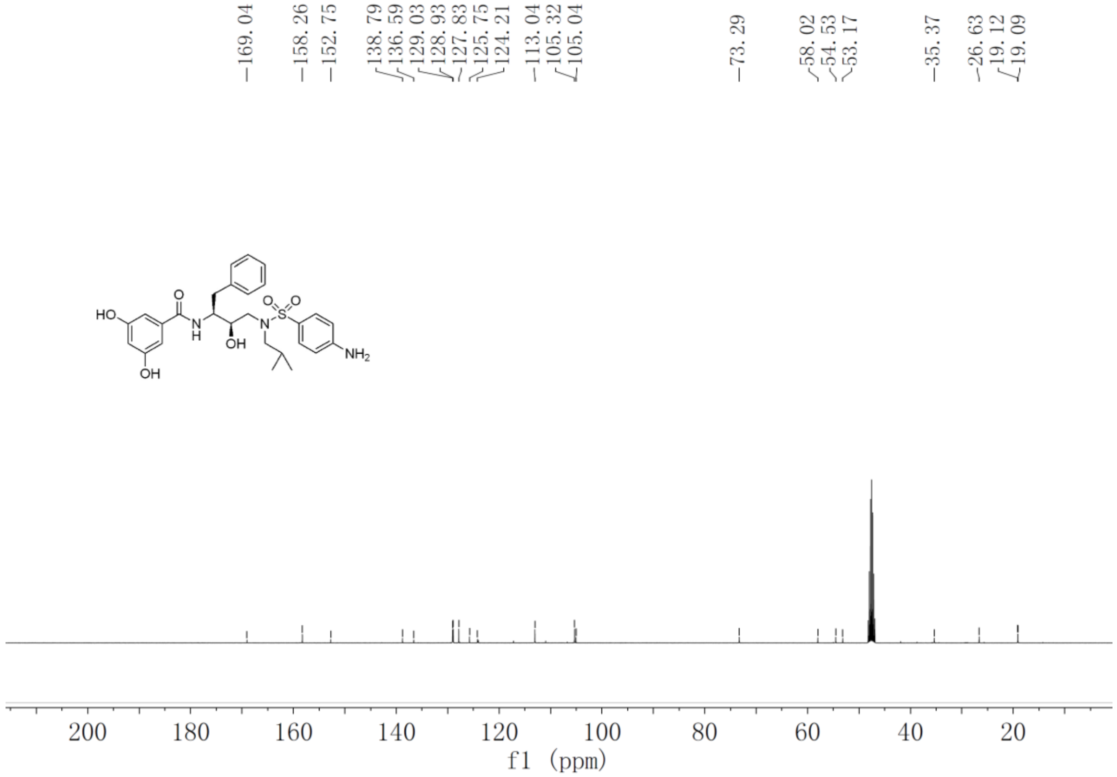

^{13}C NMR Spectrum of compound **16f**

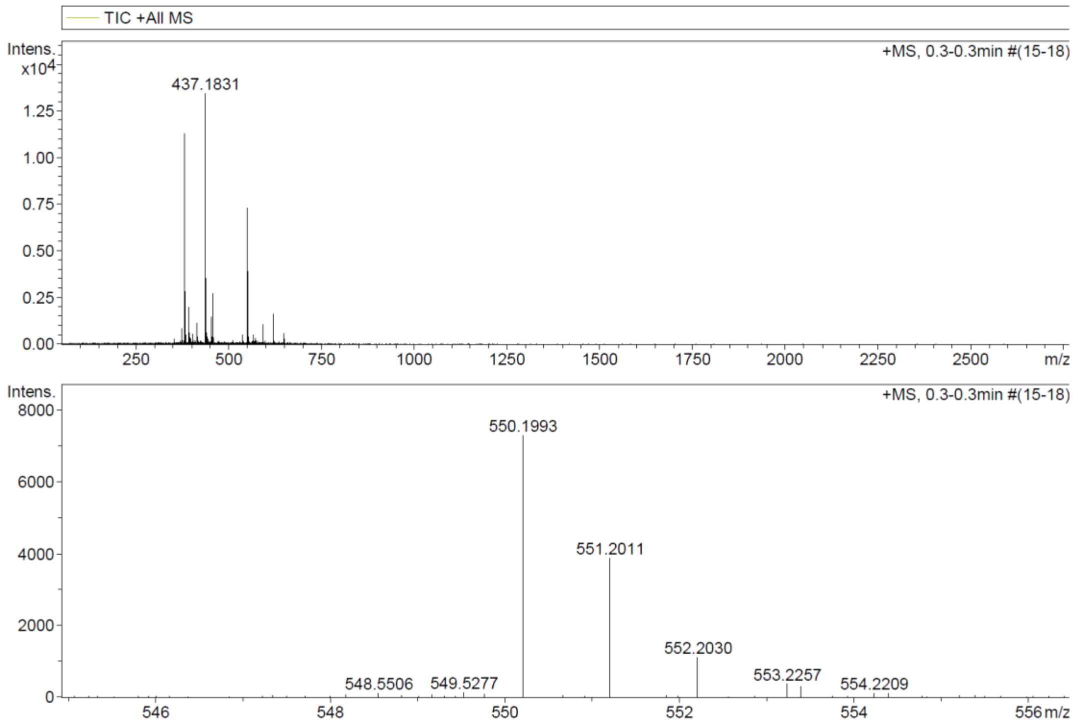

HR MS Spectrum of compound **16f**

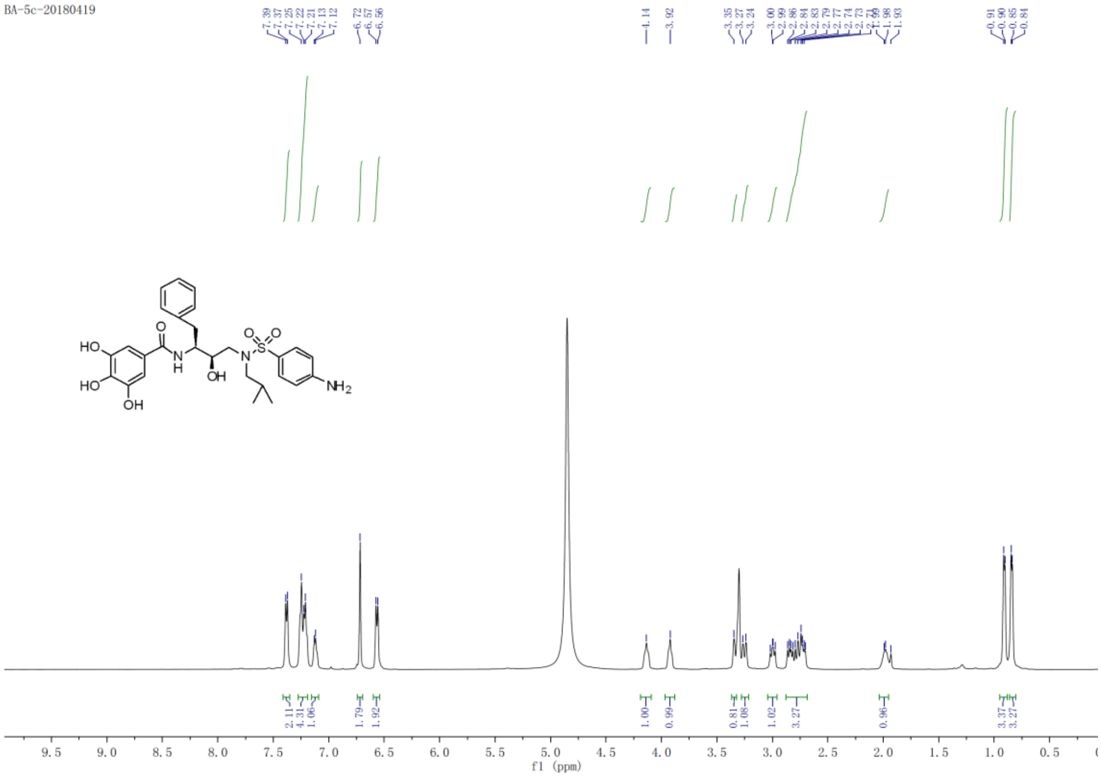

^{1}H NMR Spectrum of compound **16g**

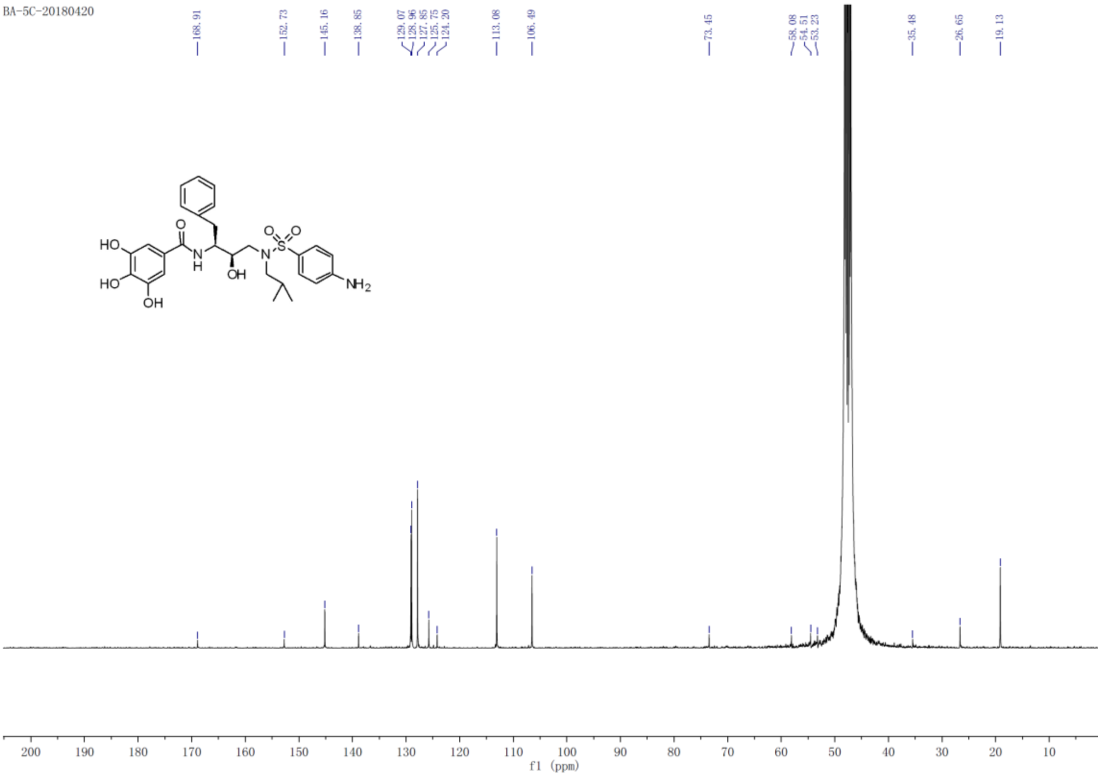

^{13}C NMR Spectrum of compound **16g**

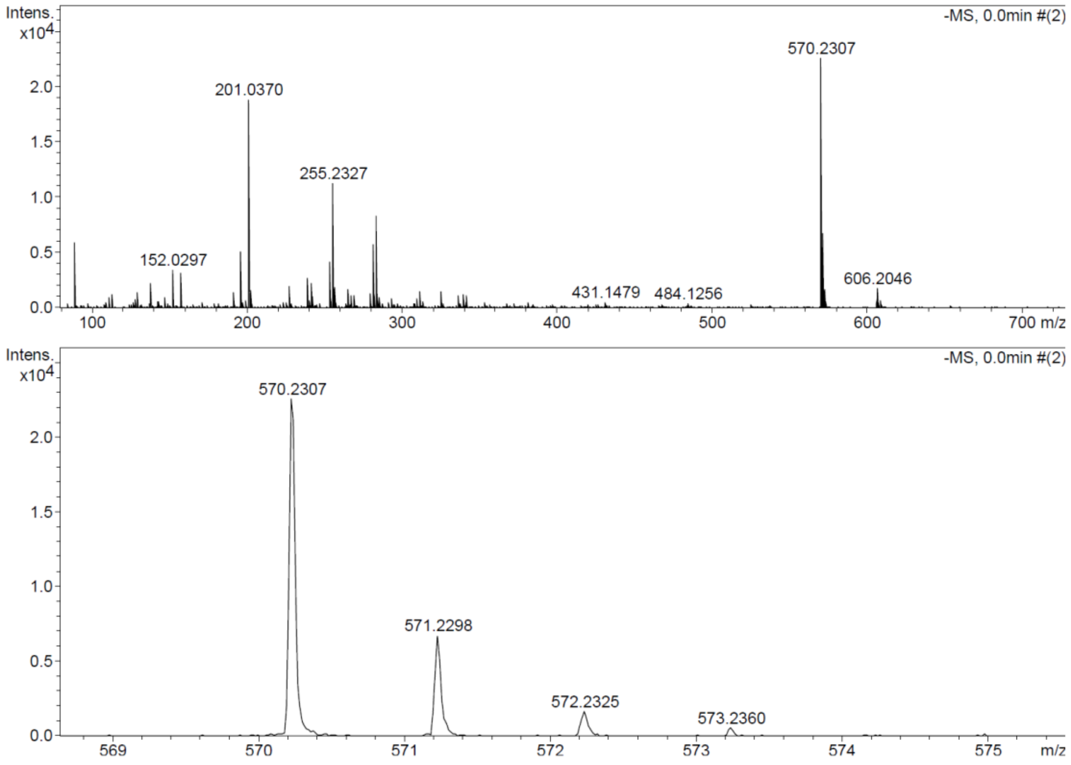

HR MS Spectrum of compound **16g**

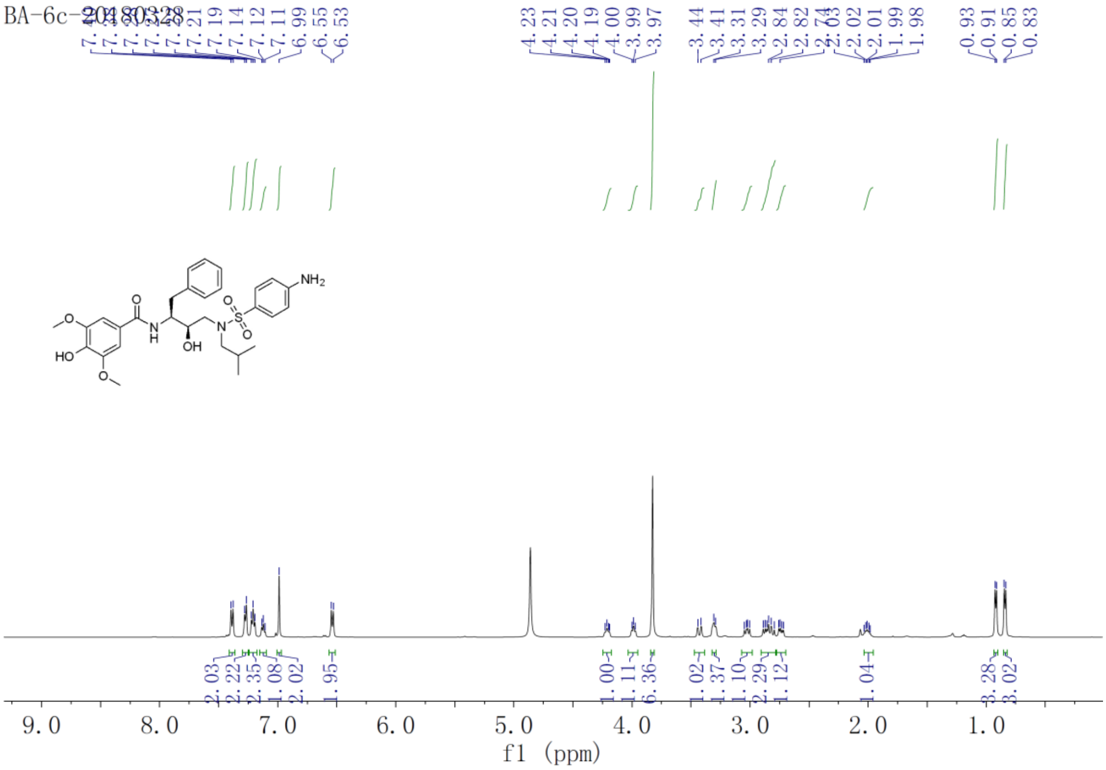

^{1}H NMR Spectrum of compound **16h**

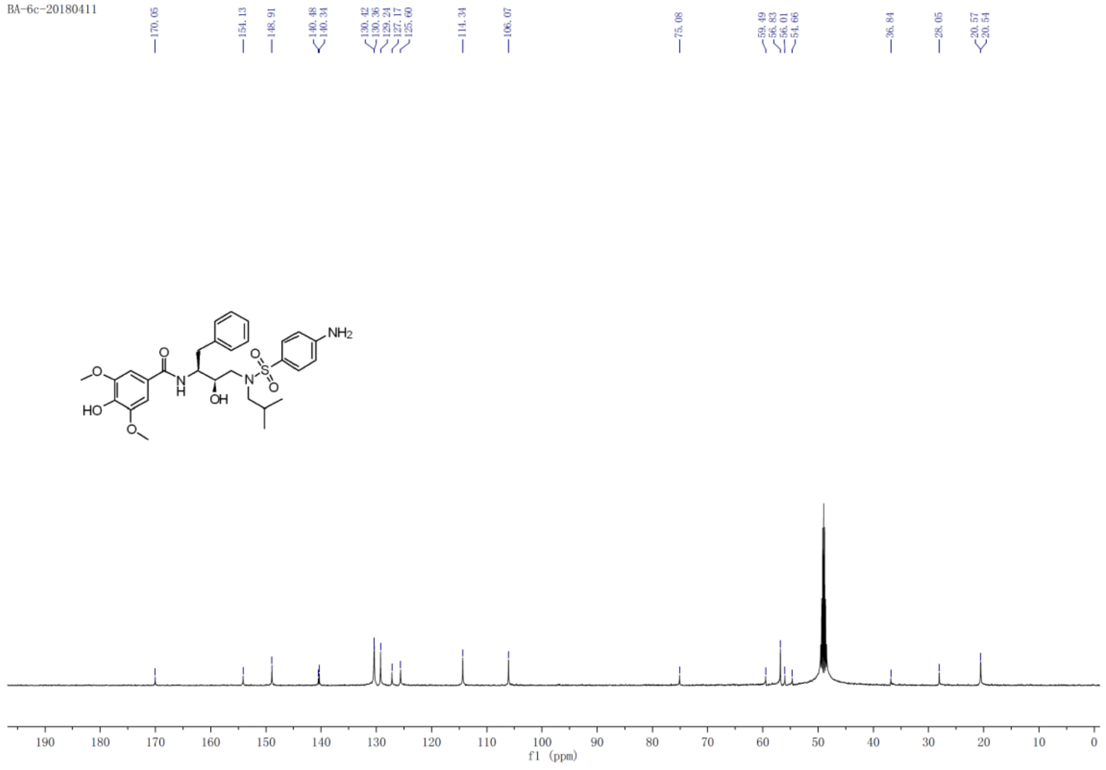

^{13}C NMR Spectrum of compound **16h**

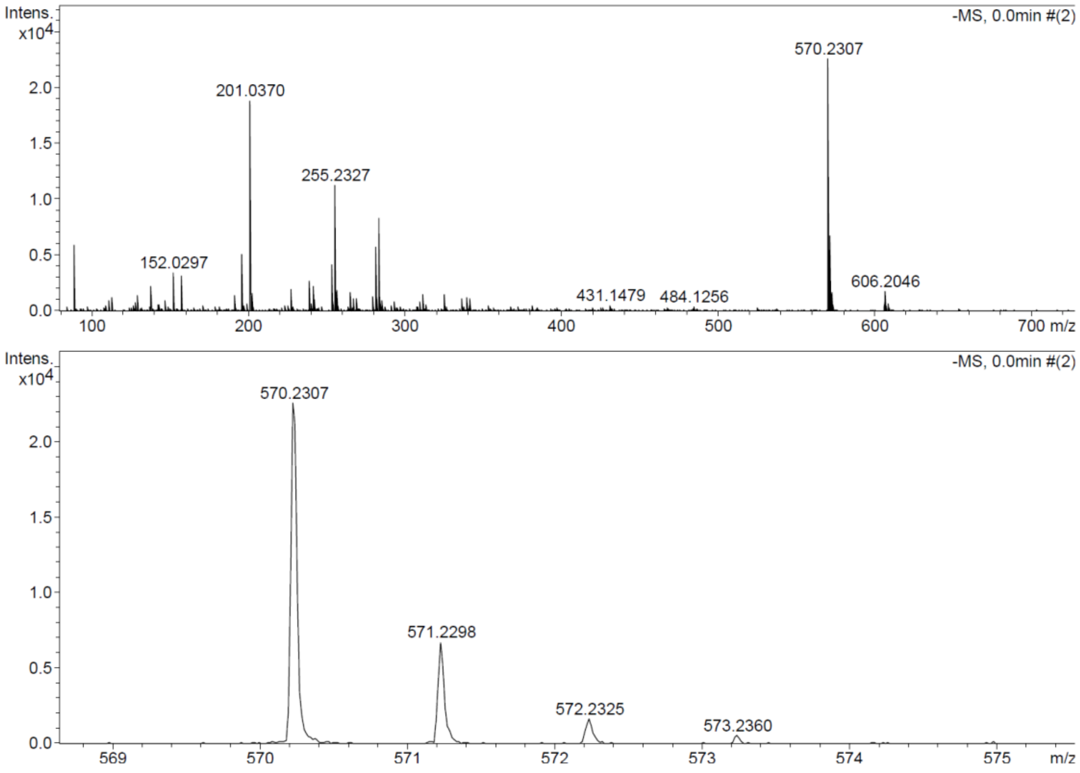

HR MS Spectrum of compound **16h**

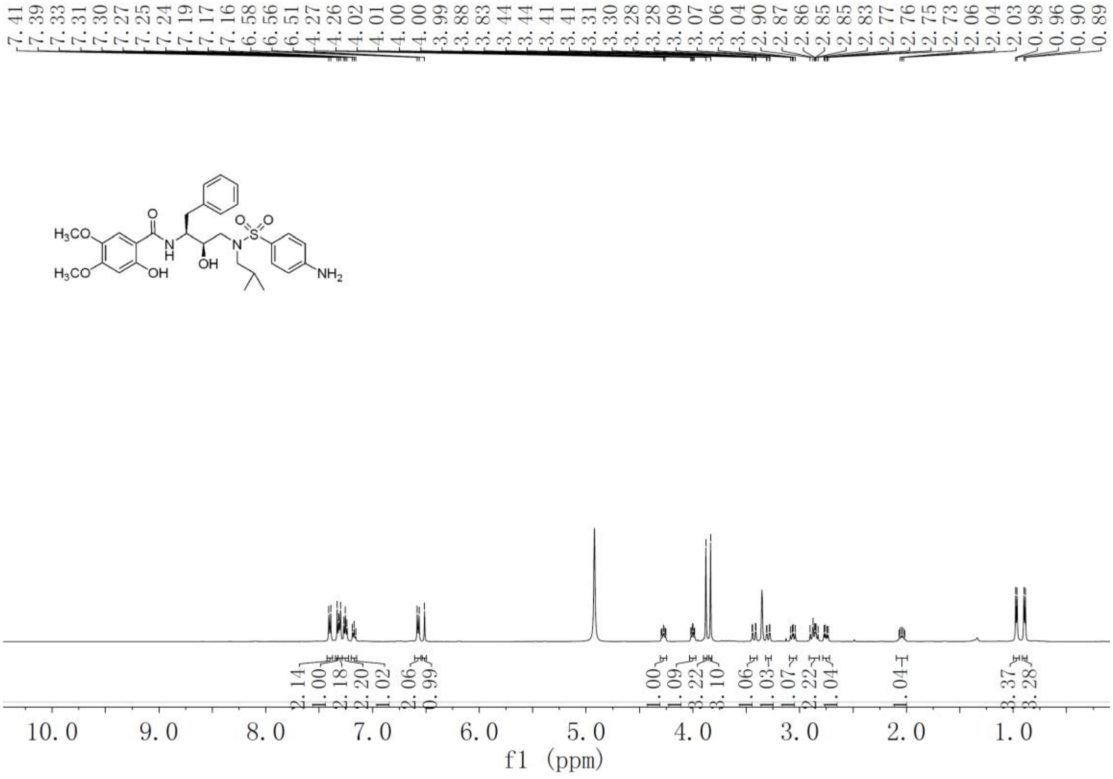

^{1}H NMR Spectrum of compound **16i**

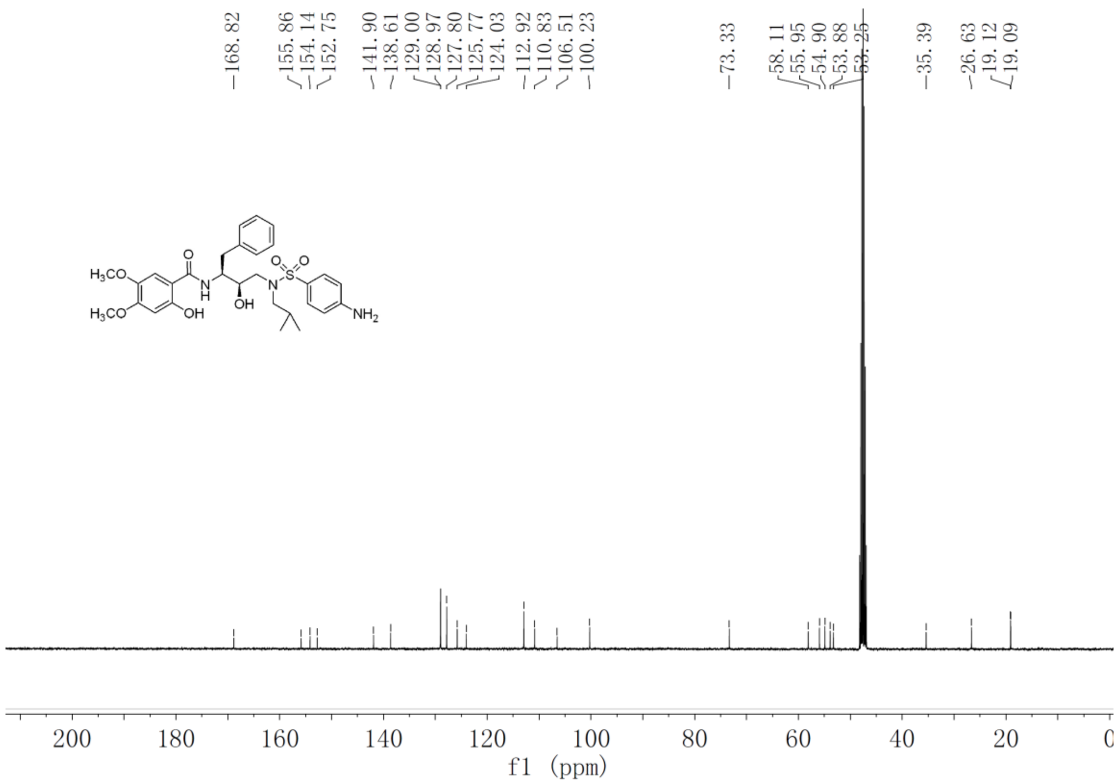

^{13}C NMR Spectrum of compound **16i**

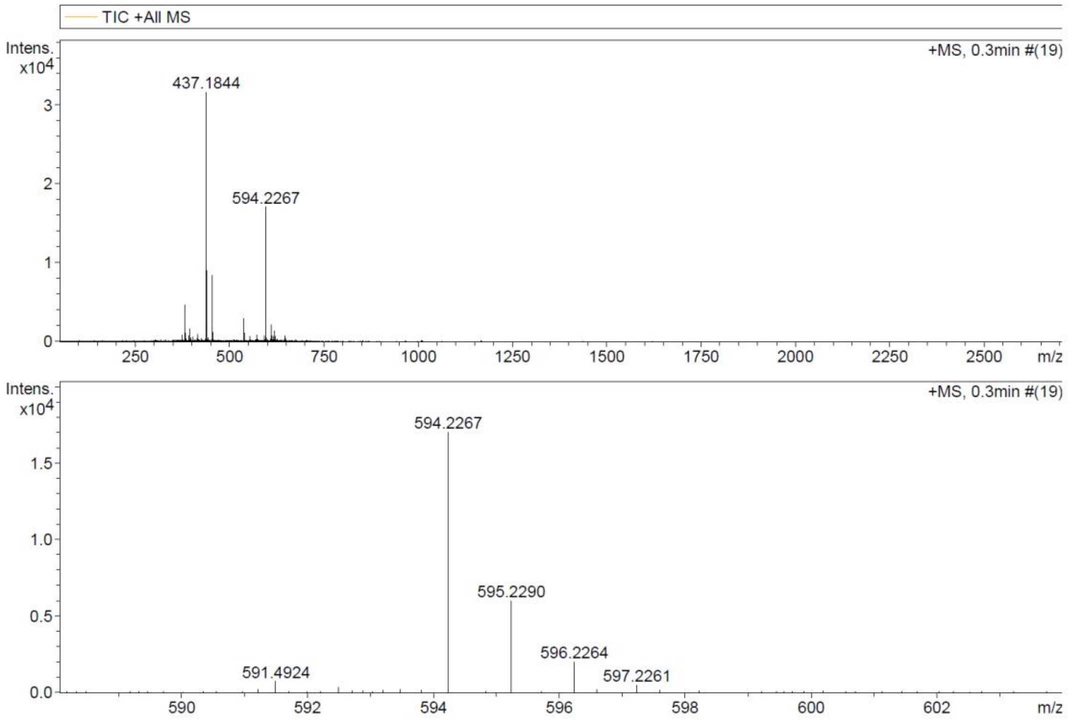

HR MS Spectrum of compound **16i**

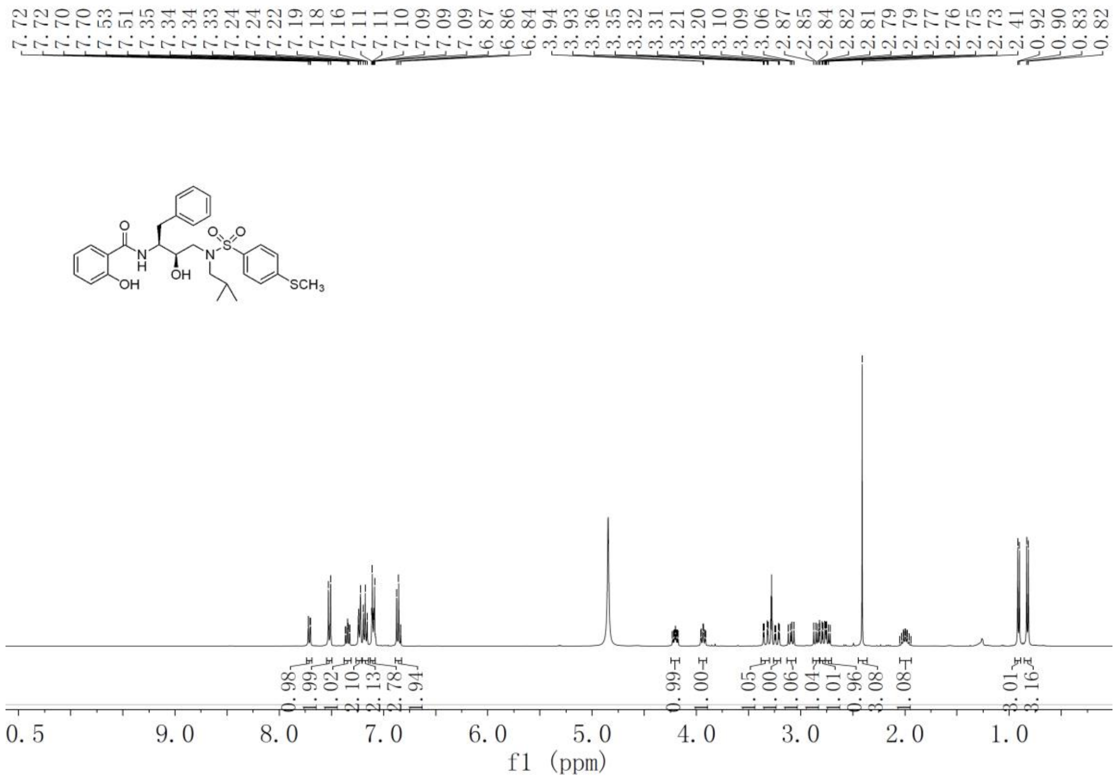

^{1}H NMR Spectrum of compound **17a**

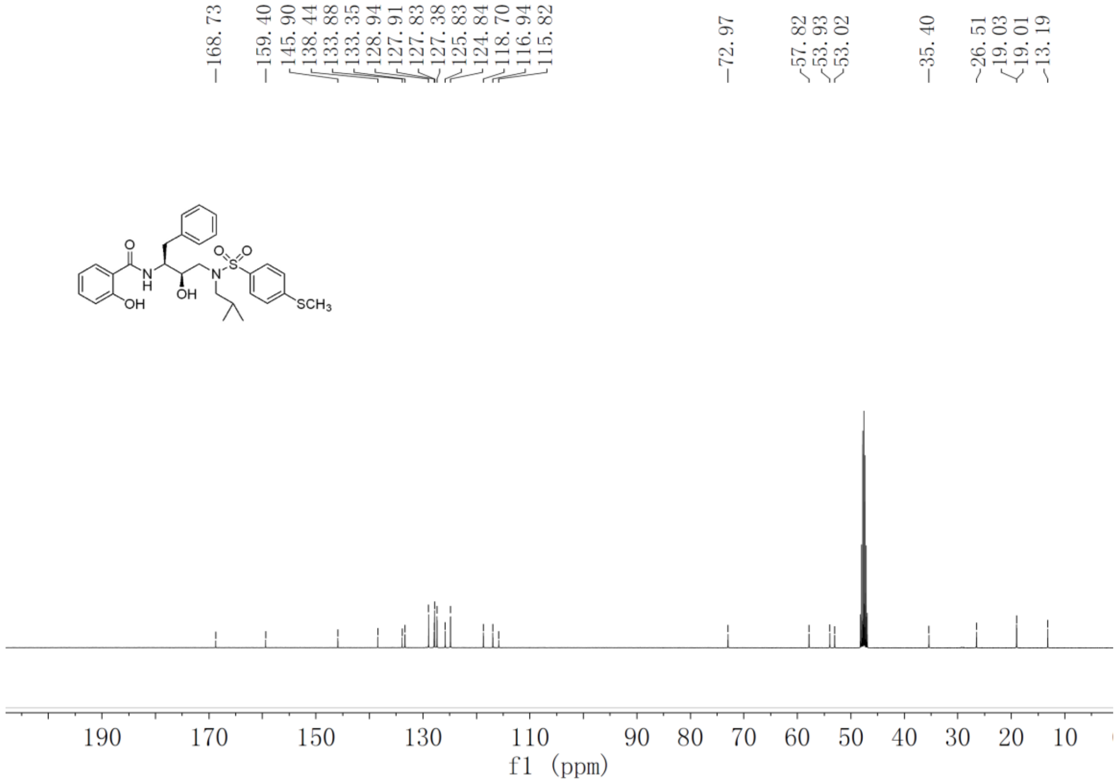

^{13}C NMR Spectrum of compound **17a**

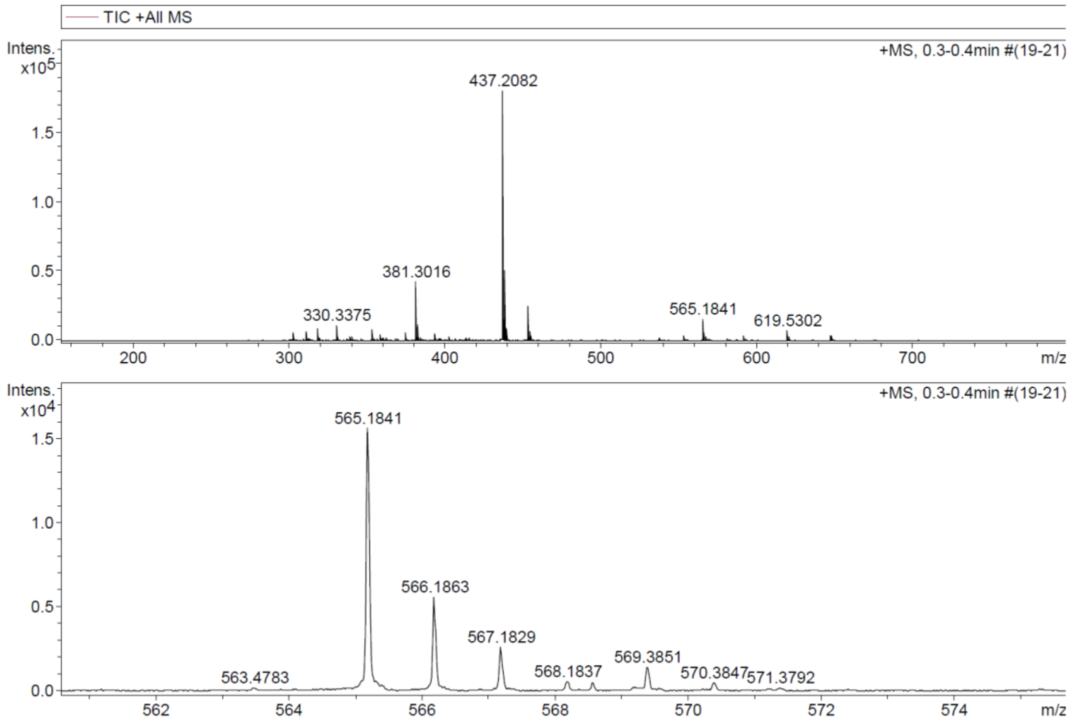

HR MS Spectrum of compound **17a**

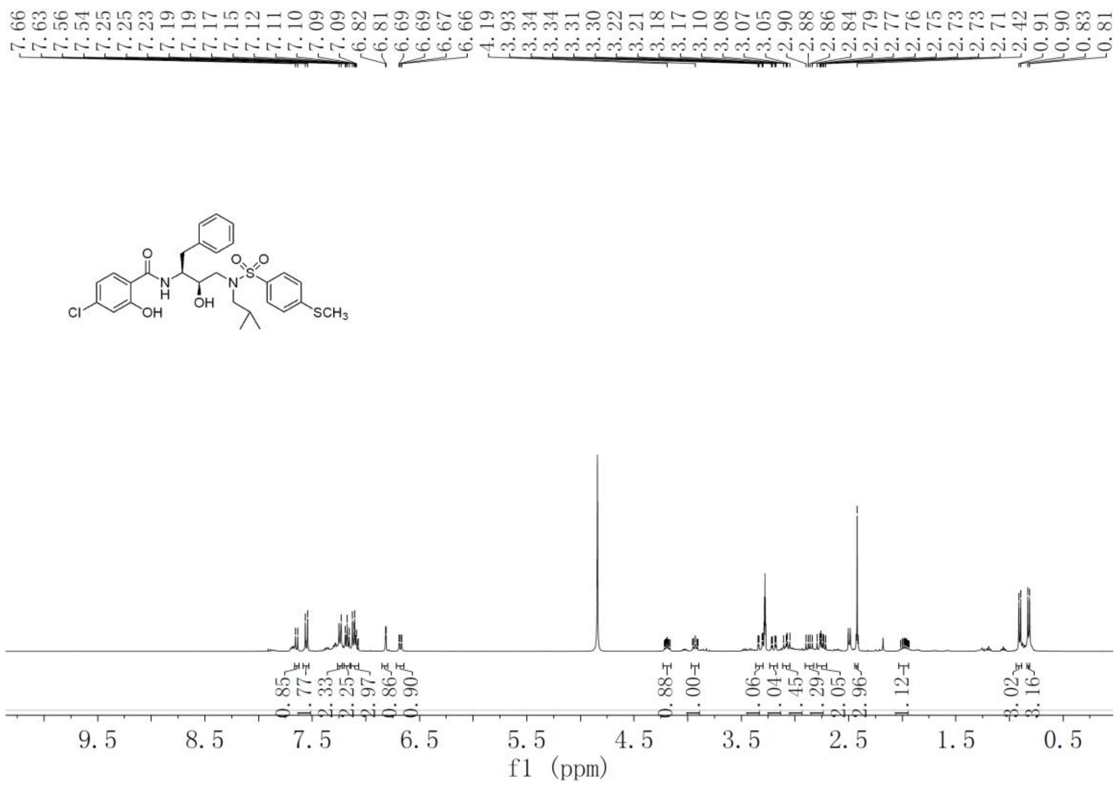

^{1}H NMR Spectrum of compound **17b**

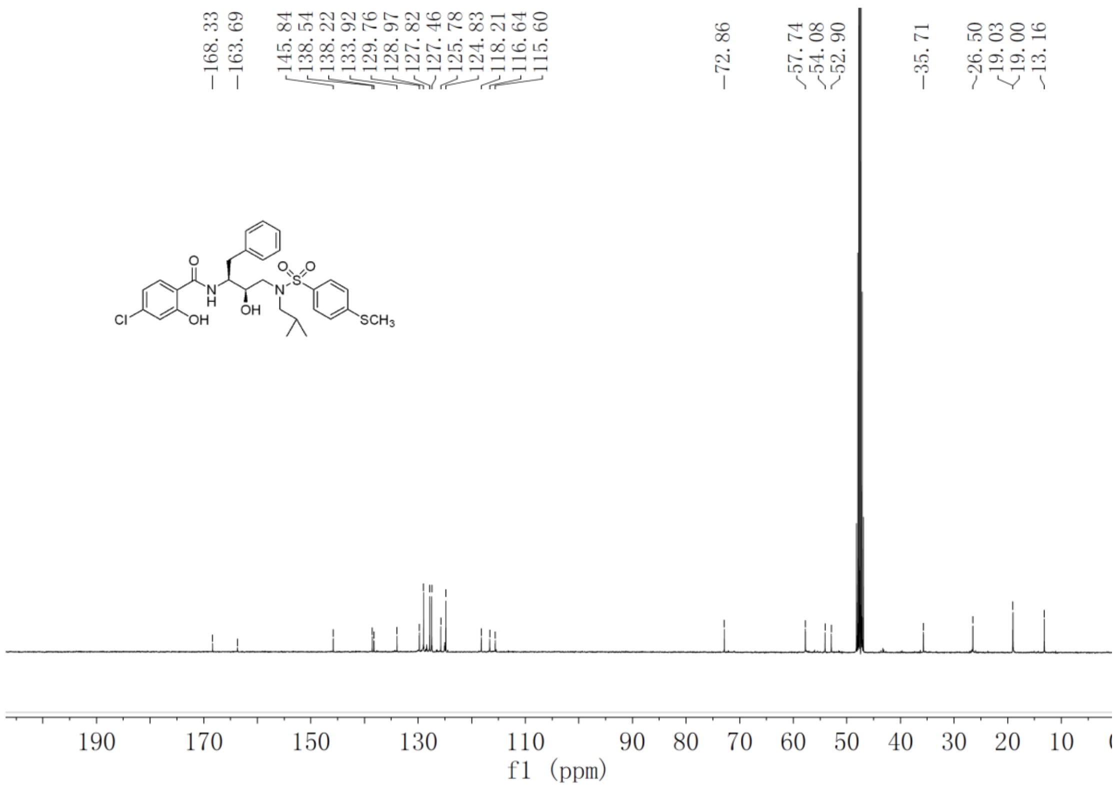

^{13}C NMR Spectrum of compound **17b**

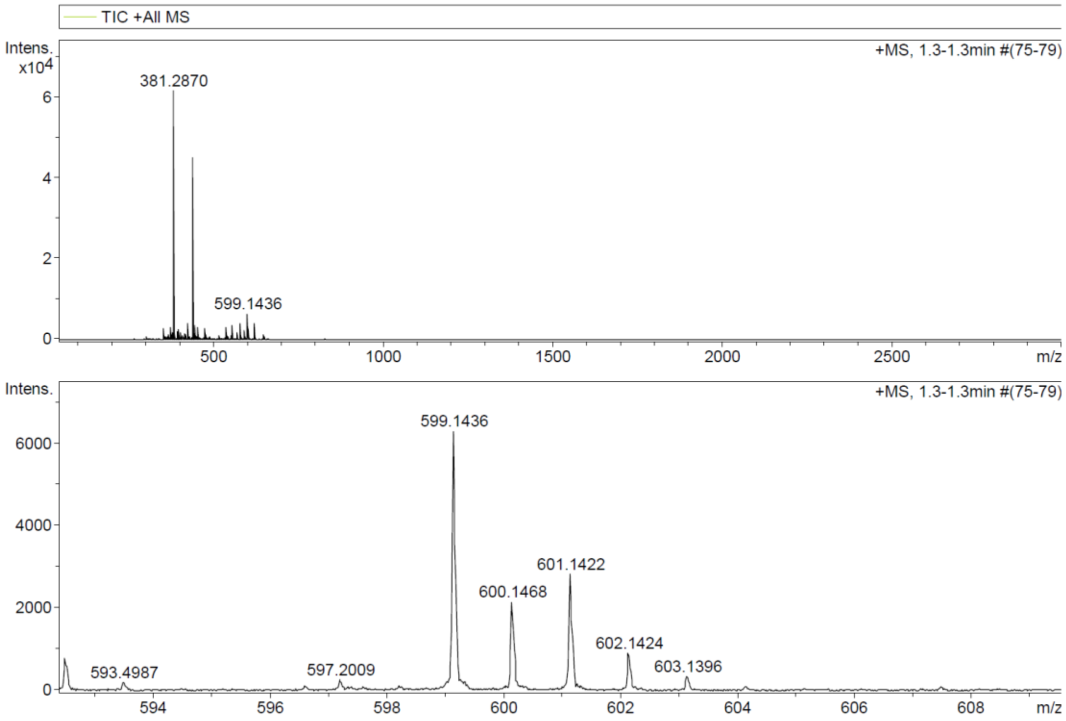

HR MS Spectrum of compound **17b**

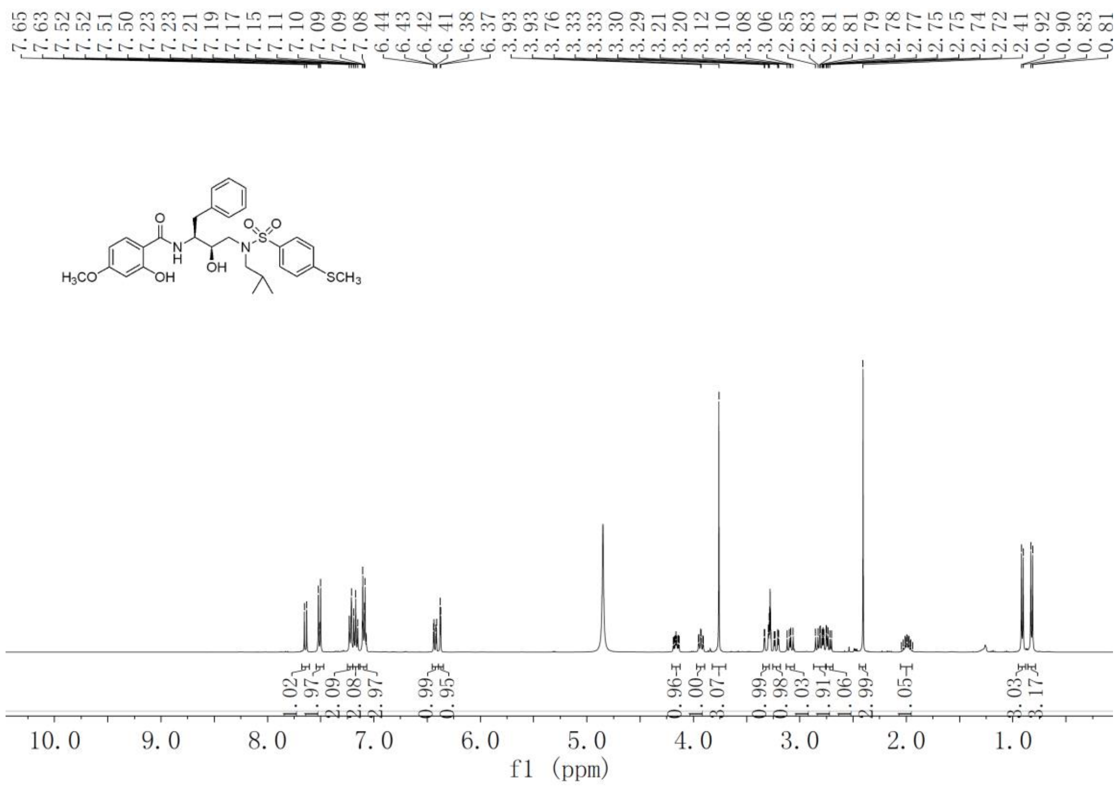

^{1}H NMR Spectrum of compound **17c**

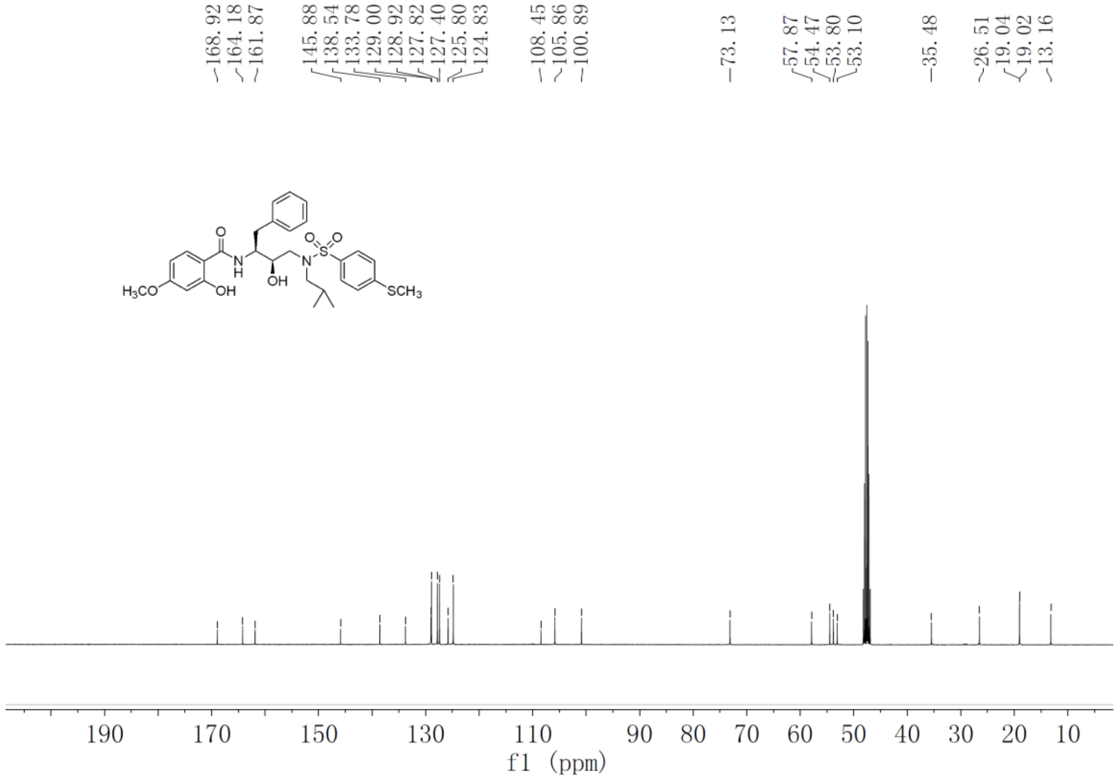

^{13}C NMR Spectrum of compound **17c**

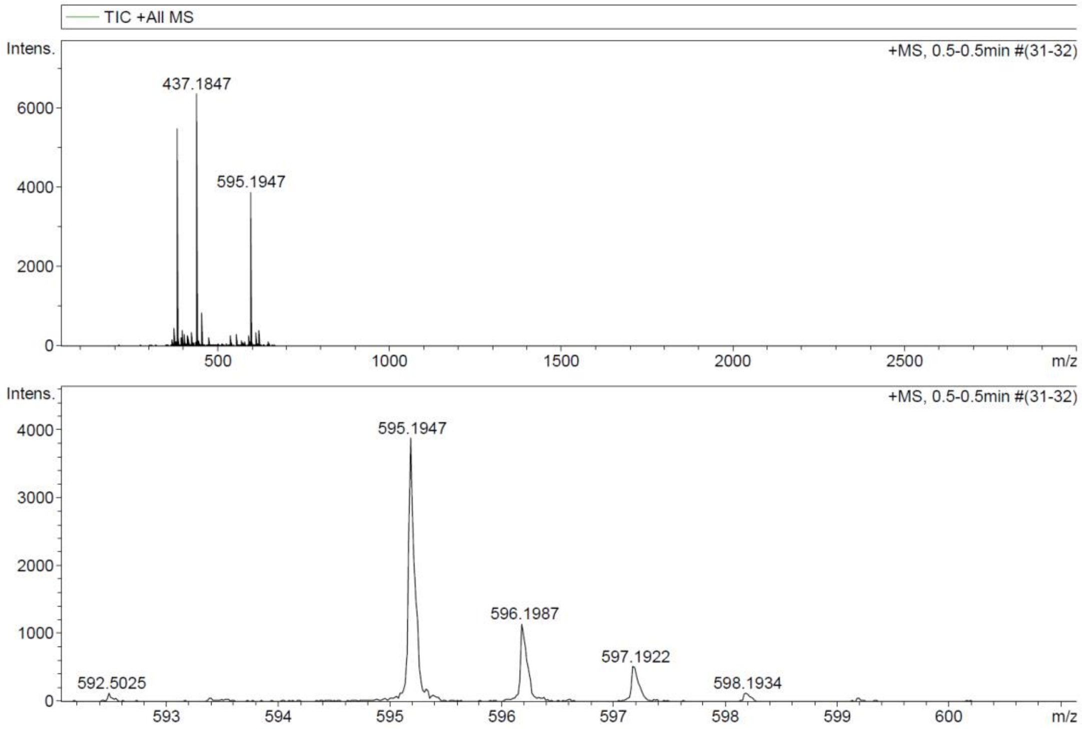

HR MS Spectrum of compound **17c**

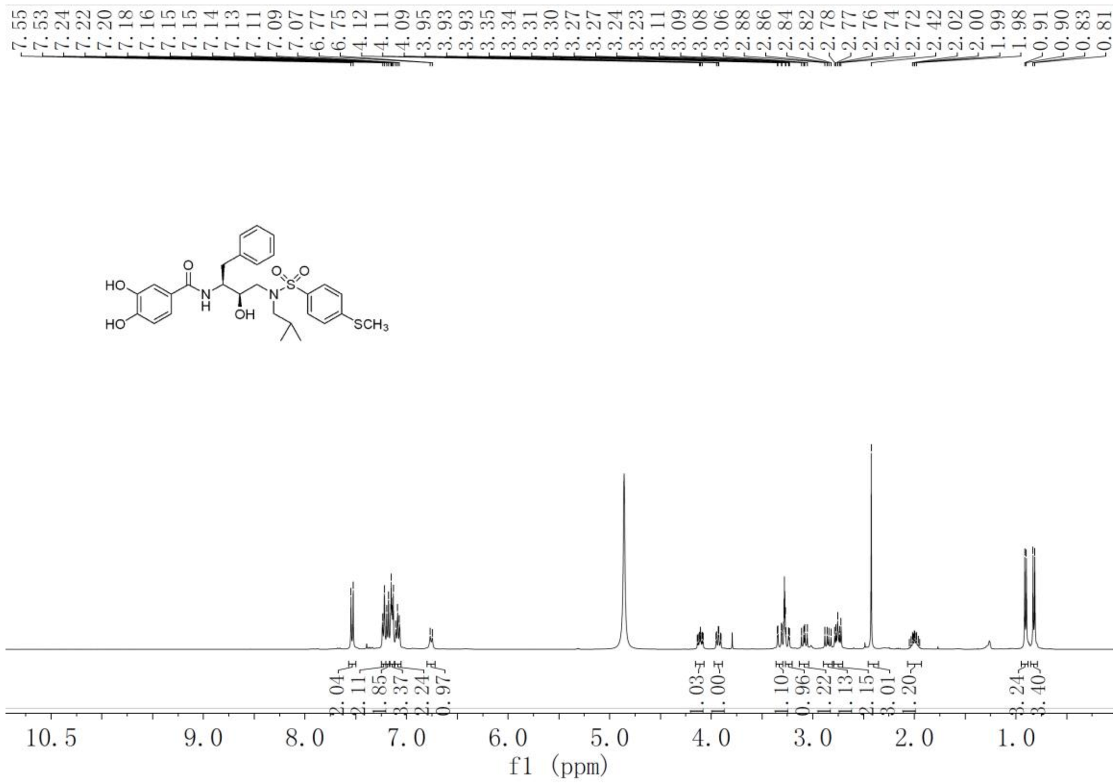

^{1}H NMR Spectrum of compound **17d**

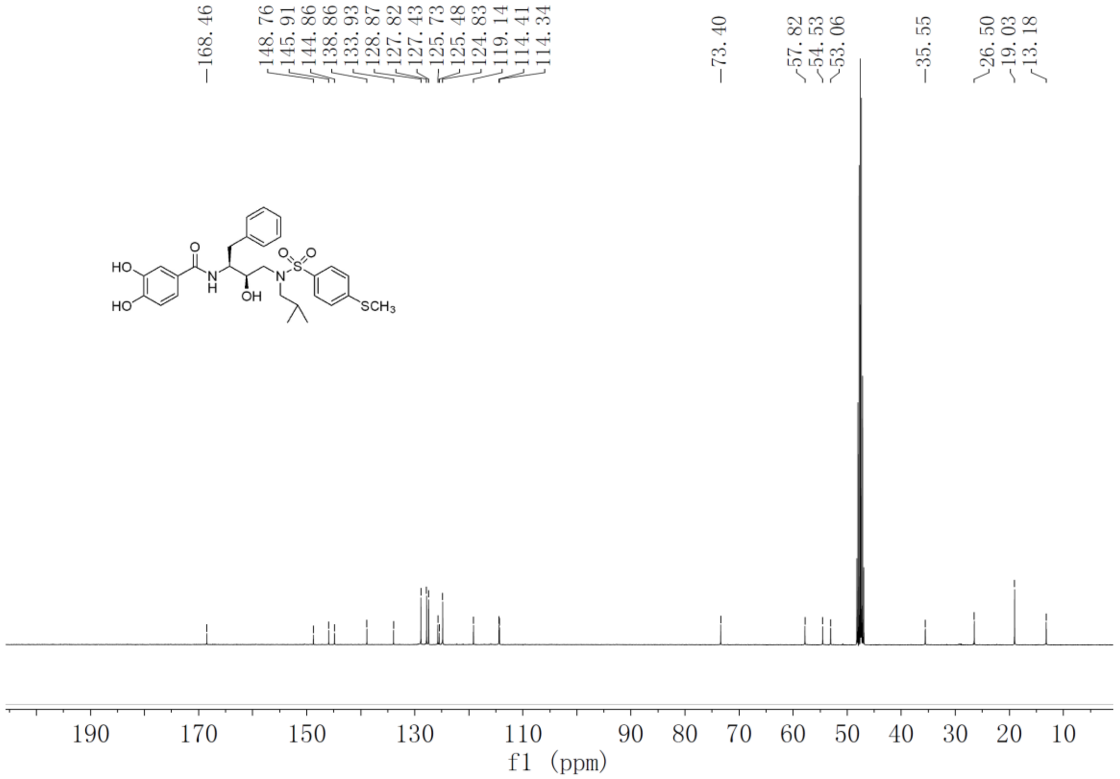

^{13}C NMR Spectrum of compound **17d**

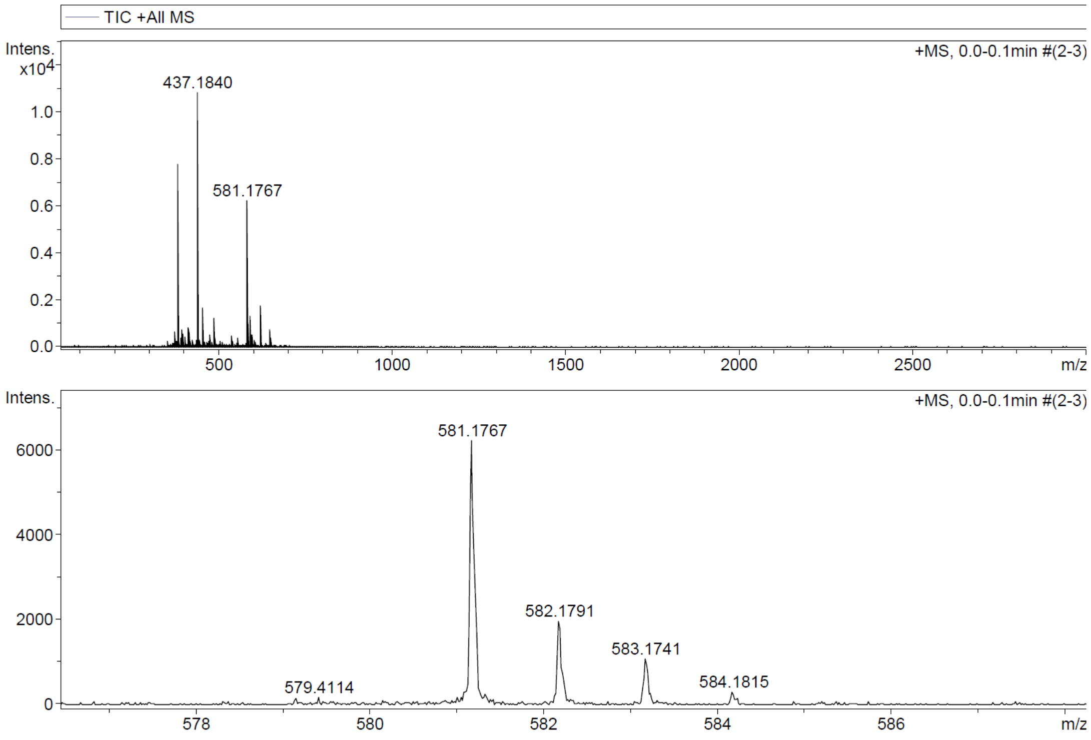

HR MS Spectrum of compound **17d**

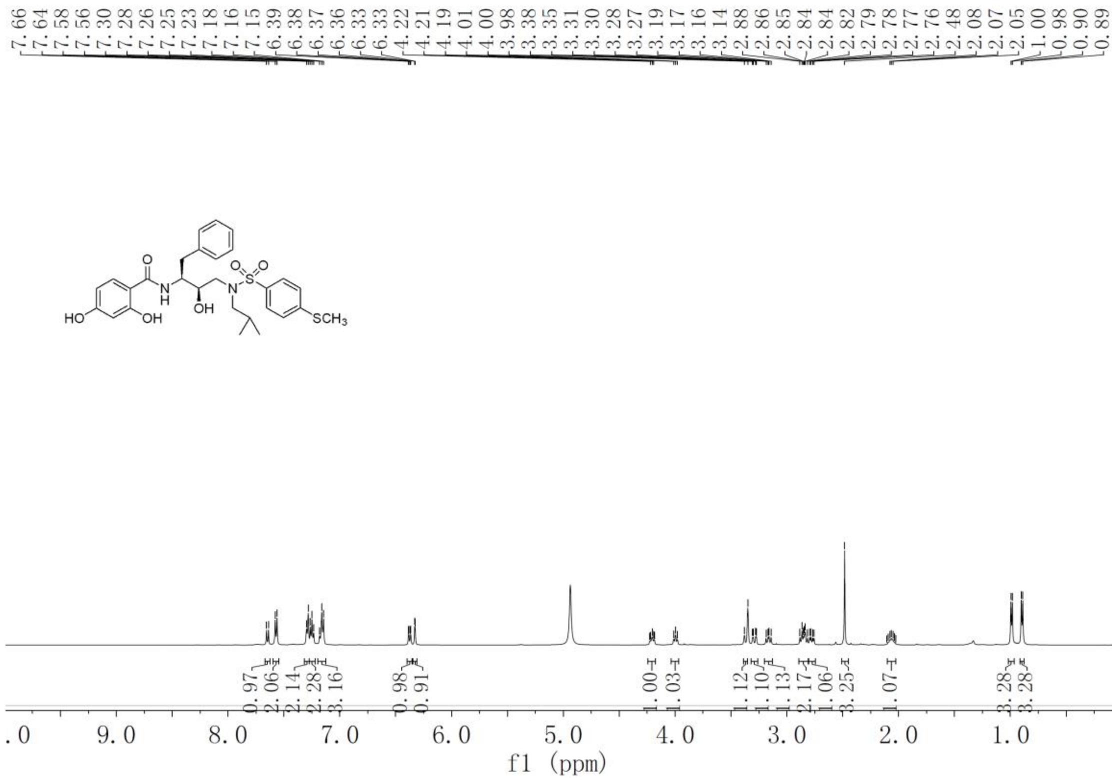

^{1}H NMR Spectrum of compound **17e**

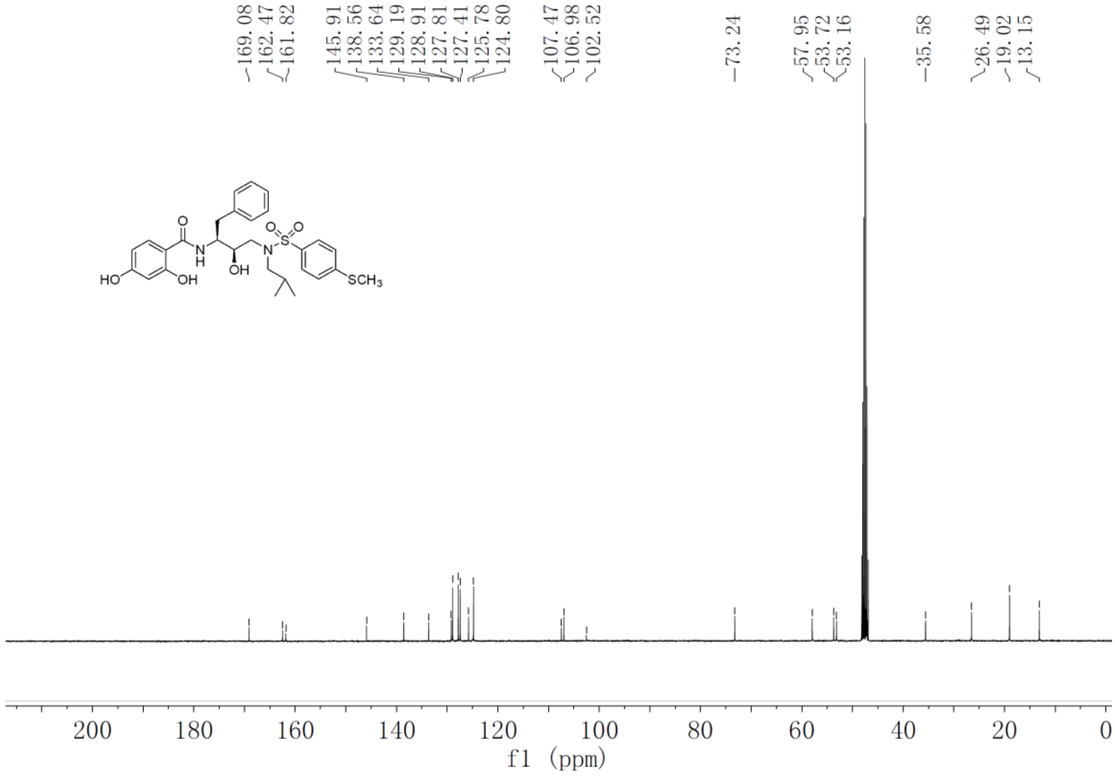

^{13}C NMR Spectrum of compound **17e**

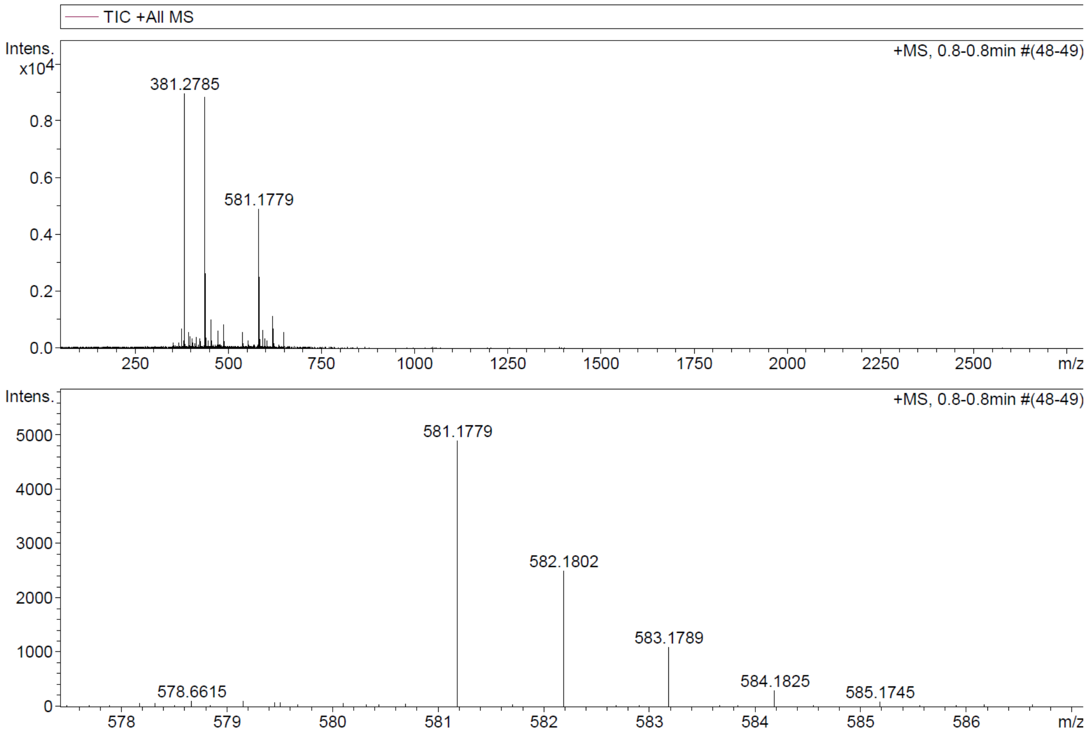

HR MS Spectrum of compound **17e**

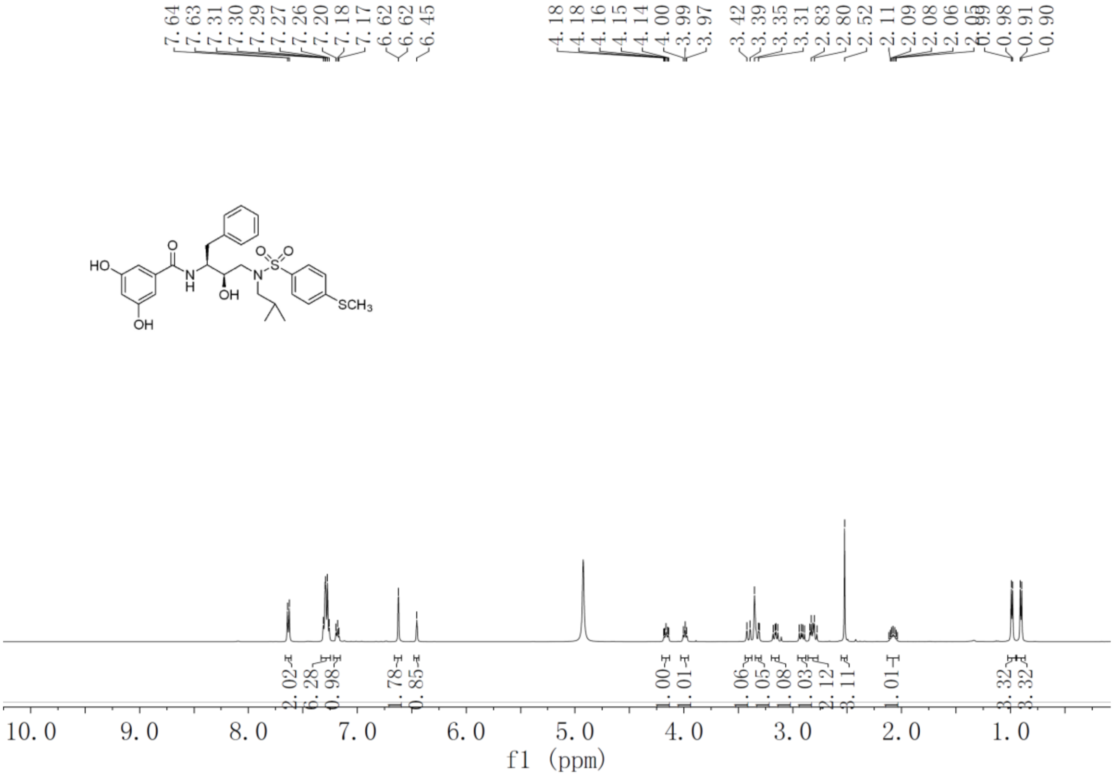

^{1}H NMR Spectrum of compound **17f**

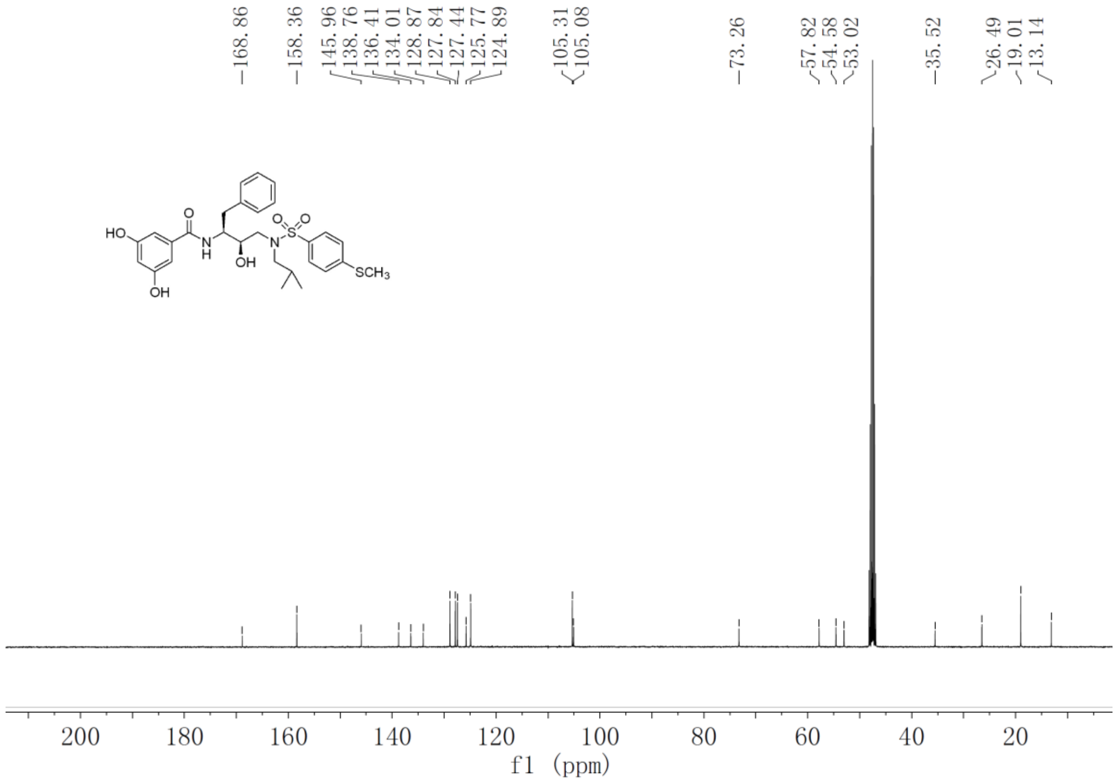

^{13}C NMR Spectrum of compound **17f**

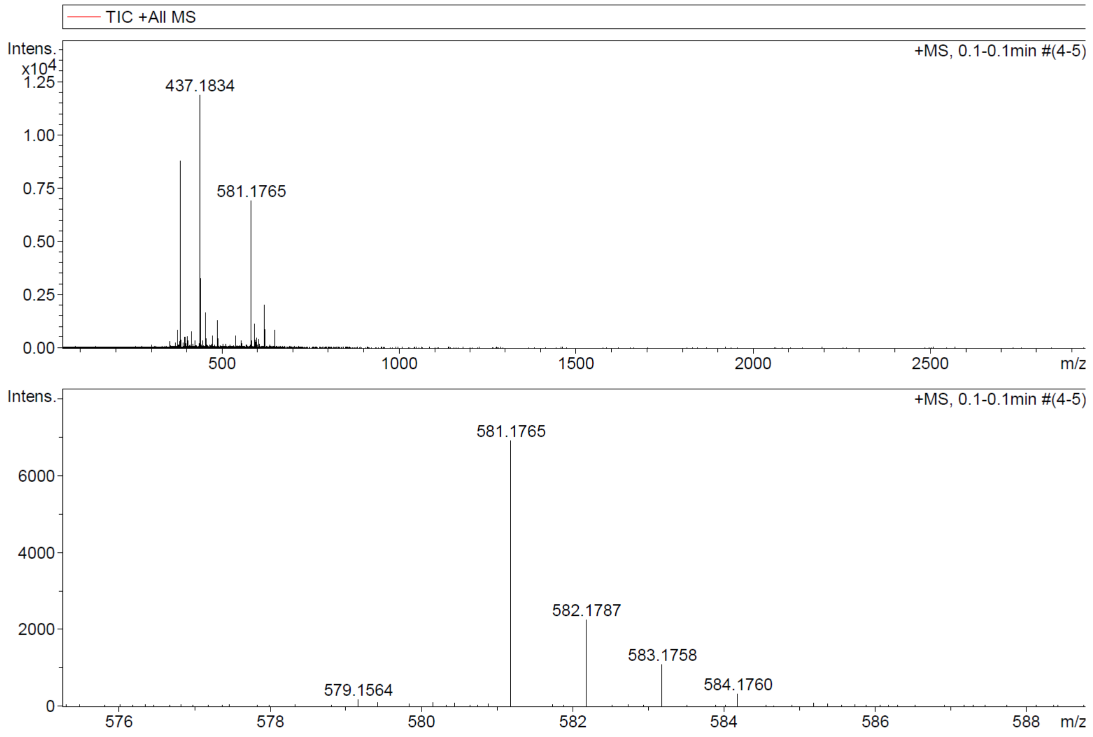

HR MS Spectrum of compound **17f**

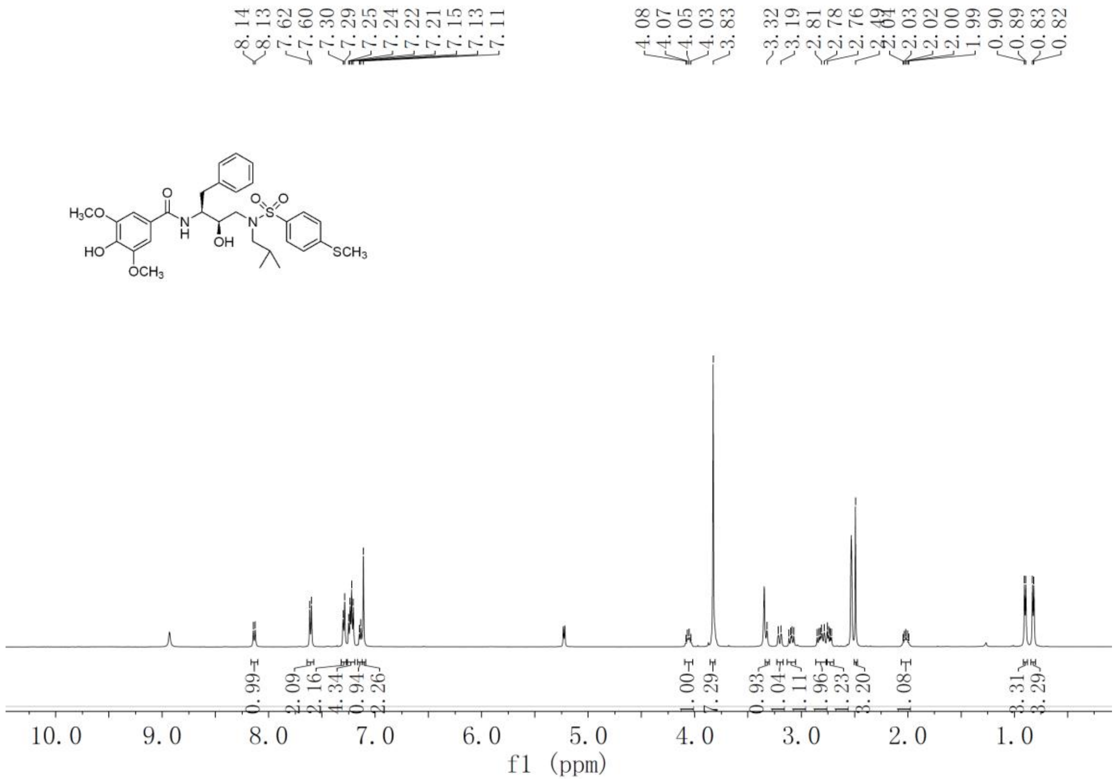

^{1}H NMR Spectrum of compound **17h**

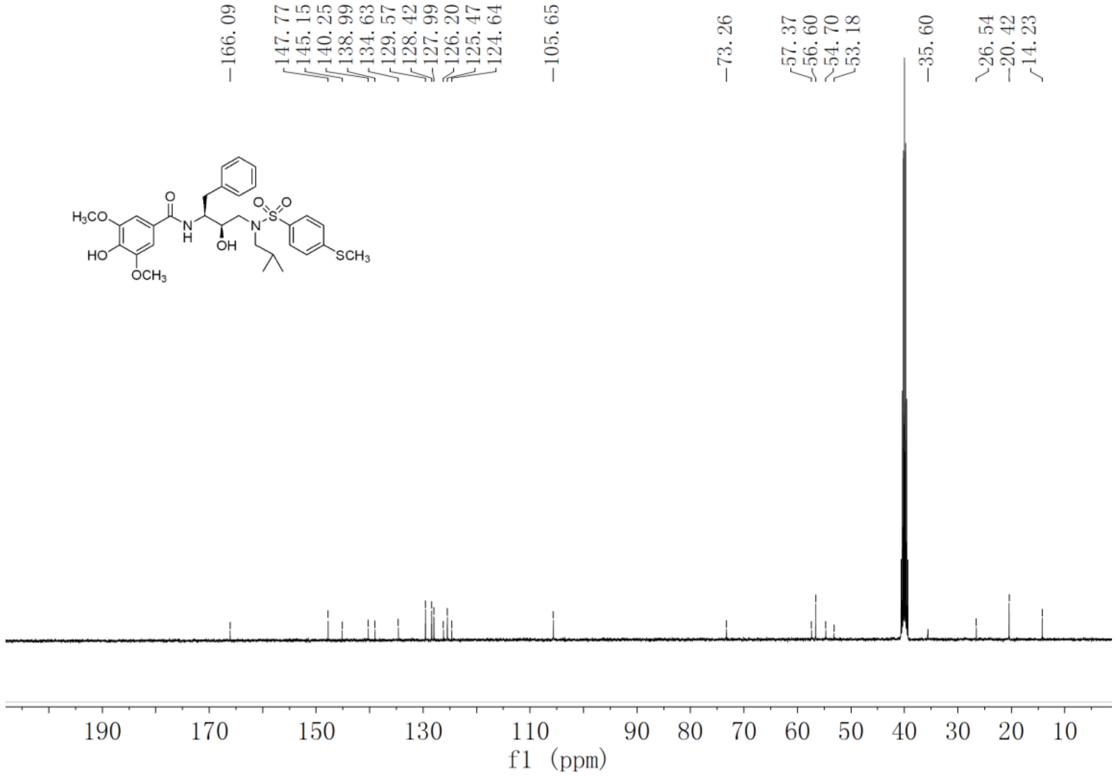

^{13}C NMR Spectrum of compound **17h**

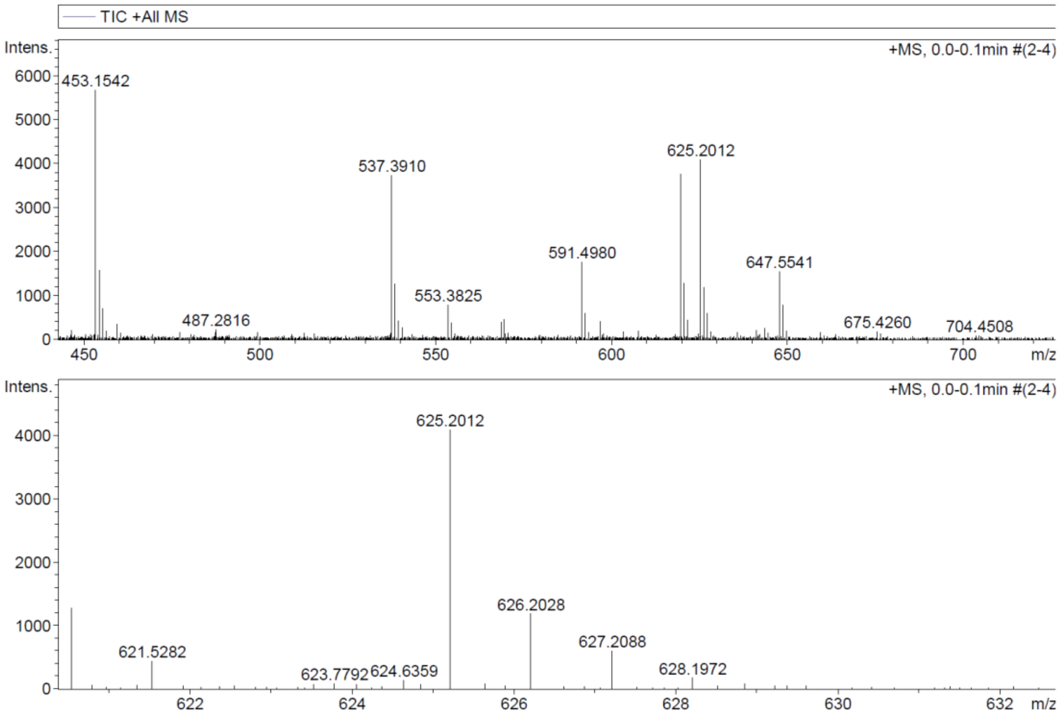

HR MS Spectrum of compound **17h**

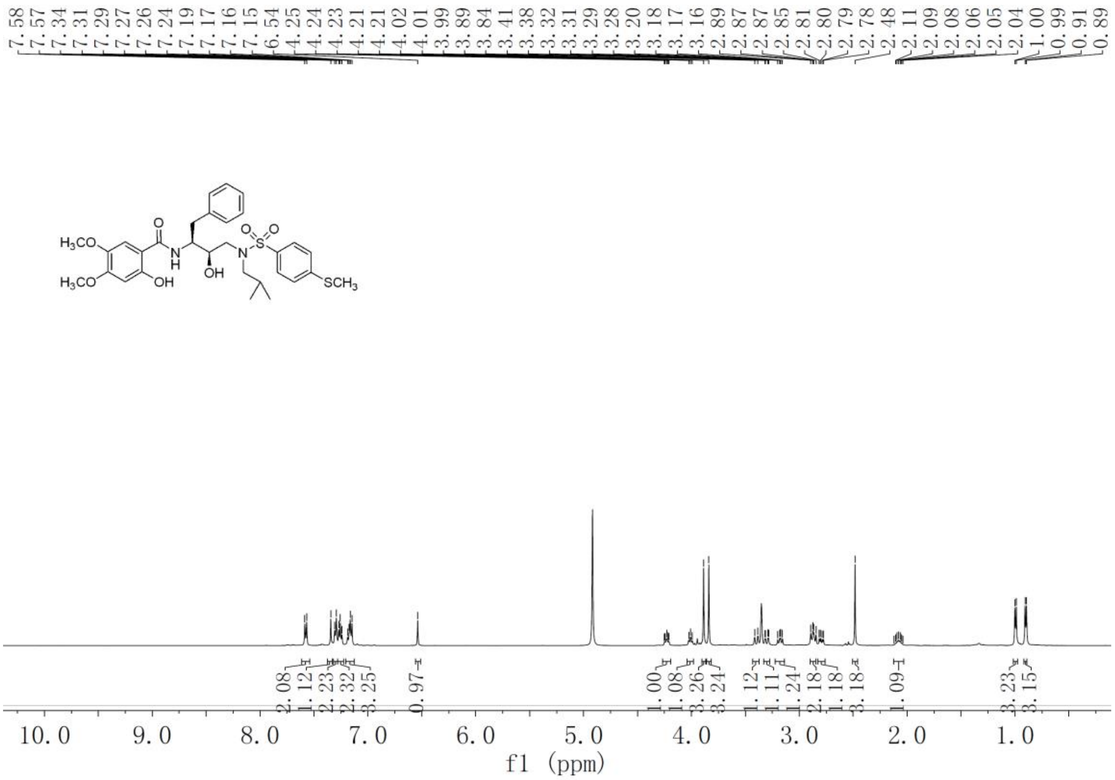

^{1}H NMR Spectrum of compound **17i**

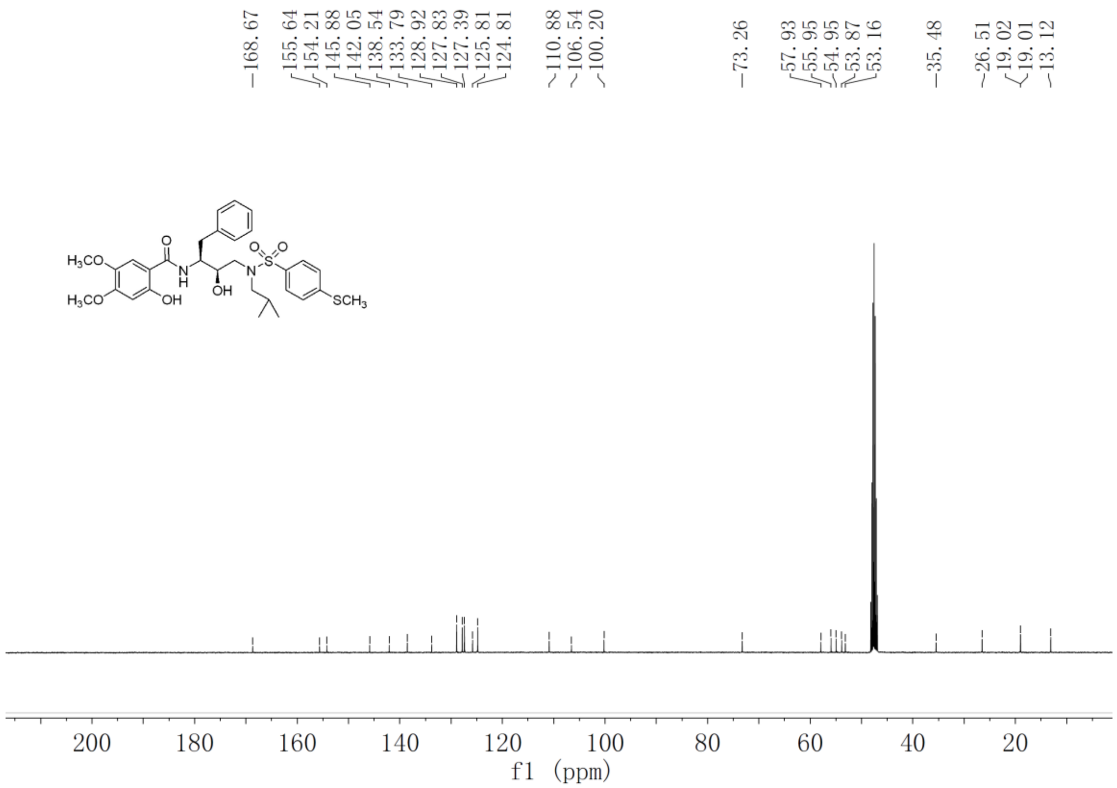

^{13}C NMR Spectrum of compound **17i**

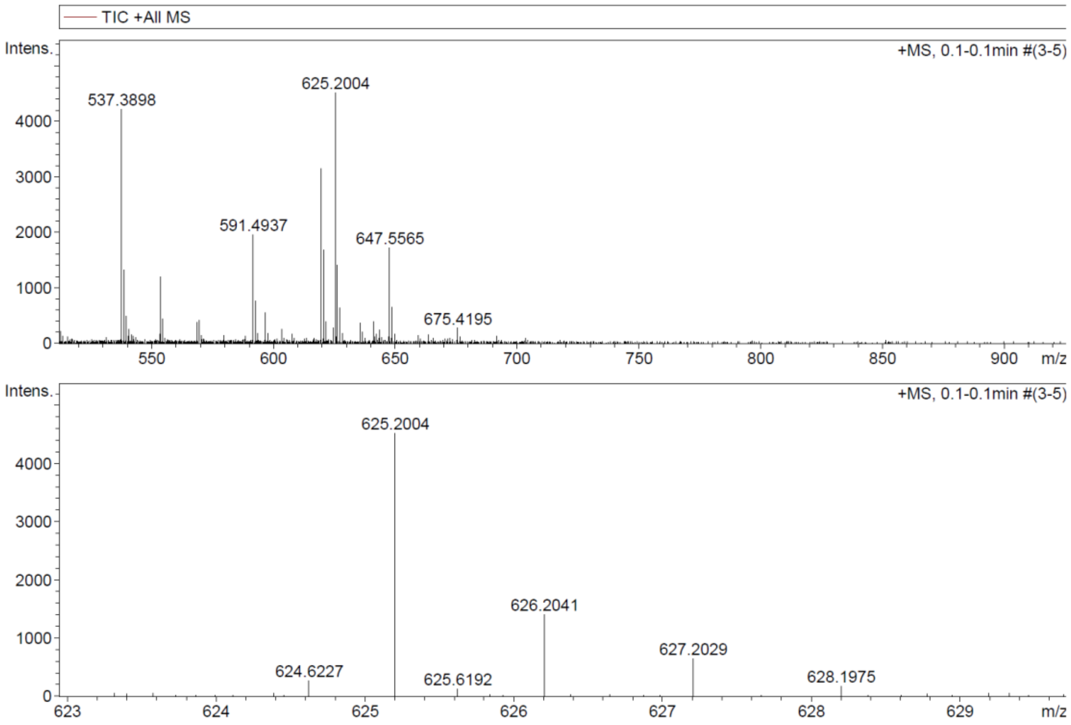

HR MS Spectrum of compound **17i**

II. Chemistry

All experiments requiring anhydrous conditions were conducted in flame-dried glassware fitted with rubber septa under a positive pressure of dry argon, unless otherwise noted. THF was distilled under argon from sodium-benzophenone ketyl, and CH2Cl2 was distilled under argon from calcium hydride. All reactions were monitored by thin-layer chromatography on silica gel plates (GF-254) and visualized with UV light. Flash column chromatography was performed on a CombiFlash®Rf 200 system employing silica gel (50–75 μm, Qingdao Haiyang Chemical Co., Ltd.). Melting points were taken on a revised MP70 Melting Point System. High-resolution mass spectra were obtained on an Autospee Ultima-TOF spectrometer. 1H NMR and 13C NMR spectra were recorded in CDCl3, CD3OD, (CD3)2CO, or DMSO-d6 on a Bruker AVANCE III 400 MHz, 500 MHz, or 600 MHz spectrometer (Bruker Inc.) with tetramethylsilane (TMS) as an internal reference. The chemical shifts are given in δ (ppm) referenced to the respective solvent peak (CDCl3: 1H, δ = 7.26 ppm, 13C, δ = 77.16 ppm; CD3OD: 1H, δ = 3.31 ppm, 13C, δ = 49.00 ppm; DMSO-d6: 1H, δ = 2.49 ppm, 13C, δ = 39.5 ppm), and coupling constants are reported in Hz. All the target compounds were characterized by 1H and 13C NMR and HRMS spectra.

2.1. Tert-butyl ((2S,3R)-3-hydroxy-4-(isobutylamino)-1-phenylbutan-2-yl)carbamate (3)

Isobutylamine (2, 19.0 mL, 189.46 mmol) was added to a stirred solution of (2S,3S)-1,2-epoxy-3-(Boc-amino)-4-phenylbutane (1, 20.0 g, 75.94 mmol) in acetonitrile (82 mL) at 25°C. The resulting mixture was heated at reflux for 6 h. After this period, the reaction mixture was concentrated under reduced pressure and the residue was purified by column chromatography on silica gel (10% MeOH in CH2Cl2 as the eluent) to provide the corresponding amine (21.2 g, 83%) as white amorphous solid: 1H NMR (400 MHz, CDCl3) δ 7.31–7.19 (m, 5H), 4.71 (d, J = 8.4 Hz, 1H), 3.87–3.75 (m, 1H), 3.48 (dd, J = 10.4, 6.0 Hz, 1H), 3.02 (d, J = 4.8 Hz, 1H), 2.99 (d, J = 4.8 Hz, 1H), 2.87 (dd, J = 13.6, 8.0 Hz, 1H), 2.75–2.68 (m, 2H), 2.49–2.38 (m, 2H), 1.83–1.66 (m, 1H),

1.35 (s, 9H), 0.93 (d, J = 6.8 Hz, 6H); 13C NMR (126 MHz, CDCl3) δ 155.9, 137.9, 129.5, 128.4, 126.3, 79.3, 70.6, 58.0, 54.2, 51.4, 36.7, 28.4, 28.3, 20.5; LC-MS (ESI, [M+H]+) m/z 337.2.

2.2. Tert-butyl ((2S,3R)-3-hydroxy-4-((N-isobutyl-4-methoxyphenyl)sulfonamido)-1-phenylbutan-2-yl)carbamate (7)

To a stirred solution of compound 3 (5.0 g, 14.86 mmol) in tetrahydrofuran (40 mL) at 0°C were added DIEA (3.68 mL, 16.34 mmol) and DMAP (0.18 g, 1.49 mmol) in batches, followed by a mixture of 4-methoxybenzenesulfonyl chloride (4, 90 mg, 0.409 mmol) and tetrahydrofuran (10 mL). The resulting mixture was stirred at 0°C for 0.5 h and at 25°C for another 3–5 h. The mixture was then concentrated under reduced pressure, extracted with ethyl acetate, and dried over anhydrous Na2SO4. Removal of solvent followed by column chromatography over a silica gel (20% EtOAc in n-hexane as the eluent) yielded compound 7 (6.14 g, 82%) as white amorphous solid: 1H NMR (400 MHz, DMSO-d6) δ 7.72 (d, J = 9.2 Hz, 2H), 7.25–7.12 (m, 5H), 7.09 (d, J = 9.2 Hz, 2H), 6.67 (d, J = 9.2 Hz, 1H), 4.95 (d, J = 6.4 Hz, 1H), 3.84 (s, 3H), 3.65–3.56 (m, 1H), 3.53–3.46 (m, 1H), 3.32 (dd, J = 14.8, 2.8 Hz, 1H), 3.01–2.96 (m, 2H), 2.83 (dd, J = 14.4, 8.4 Hz, 1H), 2.77 (dd, J = 13.6, 6.8 Hz, 1H), 2.02–1.92 (m, 1H), 1.25 (s, 9H), 0.84 (d, J = 6.4 Hz, 3H), 0.80 (d, J = 6.4 Hz, 3H); 13C NMR (101 MHz, CDCl3) δ 163.0, 156.0, 137.9, 130.0, 129.6, 129.5, 128.5, 126.4, 114.3, 79.7, 72.8, 58.6, 55.6, 54.7, 53.7, 35.5, 28.3, 27.2, 20.1, 19.9; LC-MS (ESI, [M+H]+) m/z 507.0.

2.3. Tert-butyl ((2S,3R)-3-hydroxy-4-((N-isobutyl-4-nitrophenyl)sulfonamido)-1-phenylbutan-2-yl)carbamate (8)

The compound was obtained by coupling compound 3 with 4-nitrobenzenesulfonyl chloride (5) in the presence of DIEA and DMAP in 91% yield (white amorphous solid) as described for 7: 1H NMR (400 MHz, DMSO-d6) δ 8.37 (d, J = 8.4 Hz, 2H), 8.06 (d, J = 8.4 Hz, 2H), 7.25–7.12 (m, 5H), 6.69 (d, J = 8.4 Hz, 1H), 4.96 (d, J = 6.4 Hz, 1H), 3.51–3.43 (m, 2H), 3.38–3.35 (m, 1H), 3.15 (dd, J = 13.6, 8.4 Hz, 1H), 3.08 (dd, J =

14.8, 8.8 Hz, 1H), 2.96–2.92 (m, 2H), 2.03–1.93 (m, 1H), 1.25 (s, 9H), 0.85 (d, J = 6.8 Hz, 3H), 0.83 (d, J = 6.8 Hz, 3H); 13C NMR (101 MHz, CDCl3) δ 156.4, 150.0, 145.0, 137.5, 129.4, 128.6, 128.5, 126.7, 124.3, 80.2, 72.2, 57.5, 55.2, 52.5, 35.6, 28.2, 26.9, 20.0, 19.8; LC-MS (ESI, [M+H]+) m/z 521.9.

2.4. Tert-butyl ((2S,3R)-3-hydroxy-4-((N-isobutyl-4-(methylthio)phenyl)sulfonamido)-1-phenylbutan-2-yl)carbamate (9)

The compound was obtained by coupling compound 3 with 4-(methylthio)benzenesulfonyl chloride (6) in the presence of DIEA and DMAP in 85% yield (white amorphous solid) as described for 7: 1H NMR (500 MHz, CDCl3) δ 7.65 (d, J = 7.0 Hz, 2H), 7.30–7.24 (m, 7H), 4.62 (s, 1H), 3.81–3.75 (m, 2H), 3.13–2.92 (m, 5H), 2.83 (s, 1H), 2.52 (s, 3H), 1.85 (s, 1H), 1.34 (s, 9H), 0.90 (d, J = 4.5 Hz, 3H), 0.87 (d, J = 4.5 Hz, 3H); 13C NMR (101 MHz, CDCl3) δ 156.1, 145.9, 137.8, 134.1, 129.6, 128.5, 127.7, 126.4, 125.4, 79.7, 72.7, 58.6, 54.7, 53.7, 35.5, 29.7, 28.3, 27.2, 20.1, 19.9, 14.8; LC-MS (ESI, [M+H]+) m/z 523.3.

2.5. N-((2R,3S)-3-amino-2-hydroxy-4-phenylbutyl)-N-isobutyl-4-methoxybenzenesulfonamide (10)

A solution of 7 (5.0 g, 9.87 mmol) in a mixture of 10 mL trifluoroacetic acid and 10 mL CH2Cl2 was stirred at 25°C for 3 h. After this period, the reaction mixture was concentrated under reduced pressure and the residue was redissolved in CH2Cl2 (10 mL). To this solution, saturated aqueous sodium bicarbonate was added dropwise to neutralize the superfluous acid. The mixture was then extracted with CH2Cl2 and dried over anhydrous Na2SO4. Removal of solvent under reduced pressure followed by column chromatography over a silica gel (10% CH3OH in CH2Cl2 as the eluent) yielded compound 10 (3.13 g, 78%) as white amorphous solid: 1H NMR (400 MHz, CDCl3) δ 7.89 (s, 2H), 7.67 (d, J = 8.8 Hz, 2H), 7.29–7.20 (m, 5H), 6.95 (d, J = 8.8 Hz, 2H), 4.22–4.15 (m, 1H), 3.85 (s, 3H), 3.81–3.78 (m, 1H), 3.23–3.18 (m, 1H), 3.13–3.00 (m, 3H), 2.81–2.70 (m, 2H), 1.69–1.59 (m, 1H), 0.75 (d, J = 2.8 Hz, 3H), 0.73 (d, J =

2.8 Hz, 3H); 13C NMR (101 MHz, CD3OD) δ 164.5, 140.2, 132.0, 130.6, 130.4, 129.6, 127.5, 115.3, 73.7, 58.9, 57.0, 56.2, 53.1, 39.1, 28.1, 20.5, 20.4; LC-MS (ESI, [M+H]+) m/z 407.3.

2.6. N-((2R,3S)-3-amino-2-hydroxy-4-phenylbutyl)-N-isobutyl-4-nitrobenzenesulfonamide (11)

The compound was obtained by exposing compound 8 to trifluoroacetic acid to remove the Boc group in 83% yield (white amorphous solid) as described for compound 10: 1H NMR (400 MHz, DMSO-d6) δ 8.38 (d, J = 8.0 Hz, 2H), 8.06 (d, J = 8.0 Hz, 2H), 7.82 (s, 2H), 7.38–7.27 (m, 5H), 5.64 (s, 1H), 3.91 (s, 1H), 3.49–3.37 (m, 2H), 3.07 (dd, J = 13.6, 8.4 Hz, 2H), 2.99 (dd, J = 14.0, 6.4 Hz, 1H), 2.87 (dd, J = 14.0, 6.4 Hz, 1H), 2.83–2.72 (m, 1H), 1.93–1.84 (m, 1H), 0.82 (d, J = 6.4 Hz, 3H), 0.76 (d, J = 6.4 Hz, 3H); 13C NMR (101 MHz, CD3OD) δ 151.4, 147.0, 140.0, 130.4, 129.9, 129.7, 127.5, 125.3, 72.8, 57.8, 57.2, 51.9, 39.3, 27.8, 20.3; LC-MS (ESI, [M+H]+) m/z 422.3.

2.7. 4-Amino-N-((2R,3S)-3-amino-2-hydroxy-4-phenylbutyl)-N-isobutylbenzenesulfonamide (12)

To a solution of compound 11 (1.26 g, 3.0 mmol) in CH3OH (10 mL) was added 10% Pd/C (0.13 g). The mixture was stirred at 25°C under H2 at a pressure of 50 psi for 2 h. The reaction mixture was filtered over Celite, and the filter cake was washed with CH3OH. Removal of solvent under reduced pressure followed by column chromatography on silica gel (10% CH3OH in CH2Cl2 as the eluent) afforded the corresponding aromatic amine (1.10 g, 94%) as white amorphous solid: 1H NMR (500 MHz, CD3OD) δ 7.52 (d, J = 8.6 Hz, 2H), 7.35–7.29 (m, 4H), 7.26–7.23 (m, 1H), 6.73 (d, J = 8.6 Hz, 2H), 3.83–3.80 (m, 1H), 3.37 (dd, J = 14.9, 3.8 Hz, 1H), 3.15 (dt, J = 8.8, 4.3 Hz, 1H), 3.08–3.00 (m, 2H), 2.95 (dd, J = 13.6, 7.8 Hz, 1H), 2.86 (dd, J = 13.6, 7.2 Hz, 1H), 2.58 (dd, J = 13.5, 9.4 Hz, 1H), 2.00–1.95 (m, 1H), 0.91 (d, J = 6.6 Hz, 3H), 0.88 (d, J = 6.6 Hz, 3H); 13C NMR (151 MHz, CDCl3) δ 150.5, 138.7, 130.9, 129.5, 129.3, 128.6, 126.5, 114.1, 73.0, 58.7, 55.7, 52.6, 39.0, 27.2, 20.2, 19.9; LC-MS (ESI)

[M+H]+ m/z 392.5.

2.8. N-((2R,3S)-3-amino-2-hydroxy-4-phenylbutyl)-N-isobutyl-4-(methylthio)benzenesulfonamide (13)

The compound was obtained by exposing compound 9 to trifluoroacetic acid to remove the Boc group in 81% yield (white amorphous solid) as described for compound 10: 1H NMR (500 MHz, CDCl3) δ 7.70 (d, J = 7.5 Hz, 2H), 7.30–7.21 (m, 7H), 3.75 (s, 1H), 3.30–3.21 (m, 2H), 3.14 (d, J = 4.0 Hz, 1H), 3.03–2.99 (m, 1H), 2.96 (d, J = 13.5 Hz, 1H), 2.90–2.86 (m, 1H), 2.52–2.48 (m, 4H), 1.93–1.86 (m, 1H), 0.92 (d, J = 5.0 Hz, 3H), 0.89 (d, J = 5.0 Hz, 3H); 13C NMR (101 MHz, CDCl3) δ 145.8, 138.7, 134.4, 129.3, 128.7, 127.7, 126.5, 125.5, 73.0, 58.5, 55.7, 52.6, 39.0, 27.2, 20.2, 19.9, 14.8; LC-MS (ESI) [M+H]+ m/z 423.2.

2.9. 2-Hydroxy-N-((2S,3R)-3-hydroxy-4-((N-isobutyl-4-methoxyphenyl)sulfonamido)-1-phenylbutan-2-yl)benzamide (15a)

First, N-(3-dimethylaminopropyl)-N′-ethylcarbodiimide hydrochloride (EDCI, 0.29 g, 1.5 mmol) and 1-hydroxybenzotriazole (HOBt, 0.15 g, 1.1 mmol) were sequentially added in batches to a stirred solution of 2-hydroxybenzoic acid (14a, 0.14 g, 1.0 mmol) and N-((2R,3S)-3-amino-2-hydroxy-4-phenylbutyl)-N-isobutyl-4-methoxybenzenesulfonamide (10, 0.43 g, 1.05 mmol) in dry DMF (3 mL) at 0°C under an argon atmosphere. The reaction mixture was stirred for 10 min at 0°C and then an additional 1 h at 25°C. 4-Dimethylaminopyridine (DMAP, 0.024 g, 0.20 mmol) was added and the reaction mixture was stirred for another 2 h at 25°C. The solvent was removed under reduced pressure. Water (6 mL) was added to the residue and extracted with CH2Cl2 (3 × 6 mL). The combined organic layers were dried over Na2SO4. Solvent was removed under reduced pressure followed by column chromatography over a silica gel column (30 × 6 cm). Elution with 25% ethyl acetate in n-hexane gave compound 15a (0.45 g, 85%) as white amorphous solid: 1H NMR (500 MHz, CD3OD) δ 7.74 (d, J = 7.5 Hz, 1H), 7.61 (d, J = 8.5 Hz, 2H), 7.38 (t, J = 7.5 Hz, 1H), 7.27 (d, J

= 7.5 Hz, 2H), 7.21 (t, J = 7.5 Hz, 2H), 7.13 (t, J = 7.5 Hz, 1H), 6.90 (d, J = 7.5 Hz, 2H), 6.83 (d, J = 8.5 Hz, 2H), 4.24 (dd, J = 12.0, 5.5 Hz, 1H), 3.97 (t, J = 7.5 Hz, 1H), 3.78 (s, 3H), 3.36 (d, J = 14.0 Hz, 1H), 3.26 (dd, J = 14.0, 2.5 Hz, 1H), 3.11 (dd, J = 13.0, 9.0 Hz, 1H), 2.89–2.83 (m, 2H), 2.77 (dd, J = 13.5, 6.0 Hz, 1H), 2.06–1.98 (m, 1H), 0.94 (d, J = 6.5 Hz, 3H), 0.86 (d, J = 6.5 Hz, 3H); 13C NMR (151 MHz, CD3OD) δ 170.2, 164.4, 160.7, 139.9, 134.8, 131.3, 130.6, 130.4, 129.3, 129.2, 127.3, 120.2, 118.3, 117.2, 115.2, 74.4, 59.3, 56.1, 55.4, 54.5, 36.8, 28.0, 20.5, 20.4; HRMS (ESI) m/z calculated for C28H33N2O6S ([M–H]−): 525.2054, found 525.2024.

2.10. 4-Chloro-2-hydroxy-N-((2S,3R)-3-hydroxy-4-((N-isobutyl-4-methoxyphenyl)sulfonamido)-1-phenylbutan-2-yl)benzamide (15b)

The target compound was obtained by coupling 4-chloro-2-hydroxybenzoic acid (14b) with N-((2R,3S)-3-amino-2-hydroxy-4-phenylbutyl)-N-isobutyl-4-methoxybenzenesulfonamide (10) through an EDCI/HOBt/DMAP coupling procedure in 75% yield (white amorphous solid) as described for 15a: 1H NMR (500 MHz, CD3OD) δ 7.70 (d, J = 8.5 Hz, 1H), 7.62 (d, J = 8.5 Hz, 2H), 7.26–7.19 (m, 4H), 7.12 (t, J = 7.0 Hz, 1H), 6.91–6.85 (m, 4H), 4.24 (t, J = 7.0 Hz, 1H), 3.95 (t, J = 7.0 Hz, 1H), 3.79 (s, 3H), 3.36 (d, J = 14.0 Hz, 1H), 3.24 (dd, J = 14.0, 2.5 Hz, 1H), 3.10 (dd, J = 13.5, 9.0 Hz, 1H), 2.86 (dd, J = 16.0, 7.0 Hz, 1H), 2.81–2.76 (m, 2H), 2.04–1.98 (m, 1H), 0.93 (d, J = 6.5 Hz, 3H), 0.85 (d, J = 6.5 Hz, 3H); 13C NMR (126 MHz, CD3OD) δ 167.9, 163.1, 160.1, 138.6, 138.4, 130.0, 129.3, 129.2, 129.0, 127.9, 125.9, 119.0, 116.8, 114.8, 113.8, 72.8, 57.8, 54.7, 54.0, 53.0, 35.2, 26.6, 19.1; HRMS (ESI) m/z calculated for C28H32ClN2O6S ([M–H]−): 559.1664, found 559.1668.

2.11. 2-Hydroxy-N-((2S,3R)-3-hydroxy-4-((N-isobutyl-4-methoxyphenyl)sulfonamido)-1-phenylbutan-2-yl)-4-methoxybenzamide (15c)

The target compound was obtained by coupling 2-hydroxy-4-methoxybenzoic acid (14c) with N-((2R,3S)-3-amino-2-hydroxy-4-phenylbutyl)-N-isobutyl-4-methoxybenzenesulfonamide (10) through an EDCI/HOBt/DMAP coupling procedure

in 83% yield (white amorphous solid) as described for 15a: 1H NMR (500 MHz, CD3OD) δ 7.67 (d, J = 9.0 Hz, 1H), 7.60 (d, J = 8.5 Hz, 2H), 7.27–7.19 (m, 4H), 7.13 (t, J = 7.0 Hz, 1H), 6.84 (d, J = 8.5 Hz, 2H), 6.46 (d, J = 9.0 Hz, 1H), 6.41 (s, 1H), 4.21 (t, J = 7.5 Hz, 1H), 3.96 (t, J = 7.5 Hz, 1H), 3.79 (s, 3H), 3.78 (s, 3H), 3.35 (d, J = 15.5 Hz, 1H), 3.26 (dd, J = 14.0, 2.5 Hz, 1H), 3.11 (dd, J = 13.5, 9.0 Hz, 1H), 2.88–2.80 (m, 2H), 2.76 (dd, J = 13.5, 6.0 Hz, 1H), 2.05–1.98 (m, 1H), 0.94 (d, J = 6.5 Hz, 3H), 0.86 (d, J = 6.5 Hz, 3H); 13C NMR (151 MHz, CD3OD) δ 170.4, 165.6, 164.4, 163.1, 140.0, 131.2, 130.6, 130.4, 129.2, 127.2, 115.2, 109.8, 107.4, 102.2, 74.6, 59.3, 56.1, 55.9, 55.2, 54.6, 36.8, 28.0, 20.5, 20.4; HRMS (ESI) m/z calculated for C29H35N2O7S ([M–H]−): 555.2159, found 555.2173.

2.12. 3,4-Dihydroxy-N-((2S,3R)-3-hydroxy-4-((N-isobutyl-4-methoxyphenyl)sulfonamido)-1-phenylbutan-2-yl)benzamide (15d)

The target compound was obtained by coupling 3,4-dihydroxybenzoic acid (14d) with N-((2R,3S)-3-amino-2-hydroxy-4-phenylbutyl)-N-isobutyl-4-methoxybenzenesulfonamide (10) through an EDCI/HOBt/DMAP coupling procedure in 72% yield (white amorphous solid) as described for 15a: 1H NMR (500 MHz, CD3OD) δ 7.62 (d, J = 8.5 Hz, 2H), 7.26 (d, J = 7.5 Hz, 2H), 7.21 (t, J = 7.5 Hz, 2H), 7.17 (s, 1H), 7.12 (t, J = 8.5 Hz, 2H), 6.87 (d, J = 8.5 Hz, 2H), 6.78 (d, J = 8.0 Hz, 1H), 4.15 (t, J = 7.5 Hz, 1H), 3.96 (t, J = 7.5 Hz, 1H), 3.79 (s, 3H), 3.35 (d, J = 15.0 Hz, 1H), 3.29 (d, J = 15.0 Hz, 1H), 3.09 (dd, J = 13.0, 9.0 Hz, 1H), 2.88 (dd, J = 15.0, 9.0 Hz, 1H), 2.81–2.75 (m, 2H), 2.04–1.99 (m, 1H), 0.93 (d, J = 6.5 Hz, 3H), 0.85 (d, J = 6.5 Hz, 3H); 13C NMR (151 MHz, CD3OD) δ 169.9, 164.4, 150.1, 146.2, 140.3, 131.3, 130.6, 130.3, 129.2, 127.2, 120.6, 115.9, 115.8, 115.2, 74.8, 59.3, 56.1, 55.9, 54.5, 37.0, 28.0, 20.5; HRMS (ESI) m/z calculated for C28H33N2O7S ([M–H]−): 541.2015, found 541.1991.

2.13. 2, 4-Dihydroxy-N-((2S,3R)-3-hydroxy-4-((N-isobutyl-4-methoxyphenyl)sulfonamido)-1-phenylbutan-2-yl)benzamide (15e)

The target compound was obtained by coupling 2,4-dihydroxybenzoic acid (14e) with N-((2R,3S)-3-amino-2-hydroxy-4-phenylbutyl)-N-isobutyl-4-methoxybenzenesulfonamide (10) through an EDCI/HOBt/DMAP coupling procedure in 70% yield (white amorphous solid) as described for 15a: 1H NMR (500 MHz, CD3OD) δ 7.64 (t, J = 9.0 Hz, 3H), 7.29 (d, J = 6.5 Hz, 2H), 7.25 (t, J = 7.5 Hz, 2H), 7.17 (t, J = 6.5 Hz, 1H), 6.86 (d, J = 9.0 Hz, 2H), 6.38 (dd, J = 8.5, 2.0 Hz, 1H), 6.32 (d, J = 2.0 Hz, 1H), 4.23–4.20 (m, 1H), 4.00 (t, J = 7.0 Hz, 1H), 3.82 (s, 3H), 3.35 (d, J = 1.5 Hz, 1H), 3.29 (dd, J = 14.0, 3.0 Hz, 1H), 3.15 (dd, J = 13.0, 9.0 Hz, 1H), 2.88–2.82 (m, 2H), 2.77 (dd, J = 13.0, 6.0 Hz, 1H), 2.09–2.05 (m, 1H), 0.99 (d, J = 6.5 Hz, 3H), 0.90 (d, J = 6.5 Hz, 3H); 13C NMR (101 MHz, CD3OD) δ 169.1, 163.0, 162.5, 161.9, 138.6, 129.6, 129.2, 128.9, 127.8, 125.8, 113.8, 107.5, 107.0, 102.5, 73.2, 58.0, 54.7, 53.7, 53.2, 35.5, 26.5, 19.0; HRMS (ESI) m/z calculated for C28H34N2NaO7S ([M+Na]+): 565.1984, found 565.2006.

2.14. 3,5-Dihydroxy-N-((2S,3R)-3-hydroxy-4-((N-isobutyl-4-methoxyphenyl)sulfonamido)-1-phenylbutan-2-yl)benzamide (15f)

The target compound was obtained by coupling 3,5-dihydroxybenzoic acid (14f) with N-((2R,3S)-3-amino-2-hydroxy-4-phenylbutyl)-N-isobutyl-4-methoxybenzenesulfonamide (10) through an EDCI/HOBt/DMAP coupling procedure in 72% yield (white amorphous solid) as described for 15a: 1H NMR (500 MHz, CD3OD) δ 7.68 (d, J = 9.0 Hz, 2H), 7.31–7.26 (m, 4H), 7.18 (t, J = 7.0 Hz, 1H), 6.97 (d, J = 9.0 Hz, 2H), 6.61 (d, J = 2.0 Hz, 2H), 6.45 (s, 1H), 4.19–4.15 (m, 1H), 3.99 (t, J = 7.5 Hz, 1H), 3.86 (s, 3H), 3.39 (dd, J = 15.0, 1.5 Hz, 1H), 3.35–3.31 (m, 1H), 3.14 (dd, J = 13.5, 9.0 Hz, 1H), 2.90 (dd, J = 15.0, 9.0 Hz, 1H), 2.83–2.78 (m, 2H), 2.11–2.03 (m, 1H), 0.98 (d, J = 6.5 Hz, 3H), 0.90 (d, J = 6.5 Hz, 3H); 13C NMR (101 MHz, CD3OD) δ 168.9, 163.1, 158.4, 138.8, 136.4, 129.9, 129.2, 128.9, 127.8, 125.8, 113.9, 105.3, 105.1, 73.3, 57.9, 54.7, 54.6, 53.1, 35.5, 26.5, 19.0; HRMS (ESI) m/z calculated for C28H34N2NaO7S ([M+Na]+): 565.1984, found 565.2006.

2.15. 3,4,5-Trihydroxy-N-((2S,3R)-3-hydroxy-4-((N-isobutyl-4-methoxyphenyl)sulfonamido)-1-phenylbutan-2-yl)benzamide (15g)

The target compound was obtained by coupling 3,4,5-trihydroxybenzoic acid (14g) with N-((2R,3S)-3-amino-2-hydroxy-4-phenylbutyl)-N-isobutyl-4-methoxybenzenesulfonamide (10) through an EDCI/HOBt/DMAP coupling procedure in 68% yield (white amorphous solid) as described for compound 15a: 1H NMR (500 MHz, CD3OD) δ 7.61 (d, J = 8.5 Hz, 2H), 7.26–7.20 (m, 4H), 7.12 (t, J = 7.0 Hz, 1H), 6.88 (d, J = 8.5 Hz, 2H), 6.75 (s, 2H), 4.11 (t, J = 7.5 Hz, 1H), 3.94 (t, J = 7.5 Hz, 1H), 3.86 (m, 1H), 3.80 (s, 3H), 3.27 (d, J = 14.5 Hz, 1H), 3.18 (brs, 1H), 3.09 (dd, J = 13.0, 9.0 Hz, 1H), 2.82 (dd, J = 15.5, 7.0 Hz, 1H), 2.75 (dd, J = 14.0, 7.5 Hz, 1H), 2.04–1.99 (m, 1H), 0.93 (d, J = 6.5 Hz, 3H), 0.85 (d, J = 6.5 Hz, 3H); 13C NMR (151 MHz, CD3OD) δ 170.1, 164.5, 146.7, 140.3, 138.1, 131.3, 130.7, 130.3, 129.3, 127.2, 126.1, 115.3, 107.9, 74.9, 59.4, 56.2, 55.9, 54.6, 37.0, 28.0, 20.5; HRMS (ESI) m/z calculated for C28H33N2O8S ([M–H]−): 557.1964, found 557.1968.

2.16. 4-Hydroxy-N-((2S,3R)-3-hydroxy-4-((N-isobutyl-4-methoxyphenyl)sulfonamido)-1-phenylbutan-2-yl)-3,5-dimethoxybenzamide (15h)

The target compound was obtained by coupling 4-hydroxy-3,5-dimethoxybenzoic acid (14h) with N-((2R,3S)-3-amino-2-hydroxy-4-phenylbutyl)-N-isobutyl-4-methoxybenzenesulfonamide (10) through an EDCI/HOBt/DMAP coupling procedure in 82% yield (white amorphous solid) as described for compound 15a: 1H NMR (500 MHz, DMSO-d6) δ 7.62 (d, J = 8.5 Hz, 2H), 7.27–7.19 (m, 5H), 7.07 (s, 2H), 6.89 (d, J = 8.5 Hz, 2H), 4.04 (d, J = 8.5 Hz, 1H), 3.86–3.83 (s, 1H), 3.79 (s, 6H), 3.77 (s, 3H), 3.31 (d, J = 14.5 Hz, 1H), 3.17 (d, J = 12.5 Hz, 1H), 3.04 (dd, J = 13.5, 9.0 Hz, 1H), 2.77 (dd, J = 20.0, 10.5 Hz, 2H), 2.68 (dd, J = 13.5, 6.0 Hz, 1H), 2.01–1.97 (m, 1H), 0.86 (d, J = 6.5 Hz, 3H), 0.79 (d, J = 6.5 Hz, 3H); 13C NMR (151 MHz, DMSO-d6) δ 166.2, 162.6, 147.8, 140.3, 139.0, 130.7, 129.7, 129.6, 128.4, 126.2, 124.8, 114.6, 105.7, 73.3, 57.4, 56.6, 56.0, 54.7, 53.2, 35.6, 26.6, 20.5; HRMS (ESI) m/z calculated for C30H37N2O8S ([M–H]−): 585.2265, found 585.2277.

2.17. 2-Hydroxy-N-((2S,3R)-3-hydroxy-4-((N-isobutyl-4-methoxyphenyl)sulfonamido)-1-phenylbutan-2-yl)-4,5-dimethoxybenzamide (15i)

The target compound was obtained by coupling 2-hydroxy-4,5-dimethoxybenzoic acid (14i) with N-((2R,3S)-3-amino-2-hydroxy-4-phenylbutyl)-N-isobutyl-4-methoxybenzenesulfonamide (10) through an EDCI/HOBt/DMAP coupling procedure in 80% yield (white amorphous solid) as described for compound 15a: 1H NMR (500 MHz, CD3OD) δ 7.63 (d, J = 8.5 Hz, 2H), 7.34 (s, 1H), 7.31–7.29 (m, 2H), 7.26–7.23 (m, 2H), 7.17 (t, J = 7.5 Hz, 1H), 6.86 (d, J = 8.5 Hz, 2H), 6.52 (s, 1H), 4.26–4.22 (m, 1H), 4.00 (t, J = 7.0 Hz, 1H), 3.87 (s, 3H), 3.83 (s, 3H), 3.81 (s, 3H), 3.40 (dd, J = 15.0, 2.0 Hz, 1H), 3.30 (dd, J = 14.0, 3.0 Hz, 1H), 3.15 (dd, J = 13.5, 9.0 Hz, 1H), 2.89–2.84 (m, 2H), 2.78 (dd, J = 13.5, 6.0 Hz, 1H), 2.10–2.02 (m, 1H), 0.98 (d, J = 6.5 Hz, 3H), 0.89 (d, J = 6.5 Hz, 3H); 13C NMR (101 MHz, CD3OD) δ 168.8, 163.0, 156.0, 154.2, 141.9, 138.6, 129.8, 129.2, 128.9, 127.8, 125.8, 113.8, 110.9, 106.6, 100.3, 73.3, 57.9, 56.0, 54.9, 54.7, 53.9, 53.2, 35.5, 26.5, 19.1, 19.0; HRMS (ESI) m/z calculated for C30H38N2NaO8S ([M+Na]+): 609.2247, found 609.2261.

2.18. N-((2S,3R)-4-((4-amino-N-isobutylphenyl)sulfonamido)-3-hydroxy-1-phenylbutan-2-yl)-2-hydroxybenzamide (16a)

The target compound was obtained by coupling 2-hydroxybenzoic acid (14a) with 4-amino-N-((2R,3S)-3-amino-2-hydroxy-4-phenylbutyl)-N-isobutylbenzenesulfonamide (12) through an EDCI/HOBt/DMAP coupling procedure in 80% yield (white amorphous solid) as described for compound 15a: 1H NMR (500 MHz, CD3OD) δ 7.73 (d, J = 8.0 Hz, 1H), 7.36 (t, J = 10.0 Hz, 3H), 7.27 (d, J = 7.5 Hz, 2H), 7.21 (t, J = 7.5 Hz, 2H), 7.13 (t, J = 7.0 Hz, 1H), 6.88 (t, J = 7.5 Hz, 2H), 6.54 (d, J = 8.0 Hz, 2H), 4.28 (t, J = 6.5 Hz, 1H), 3.97 (t, J = 6.5 Hz, 1H), 3.38 (d, J = 15.0 Hz, 1H), 3.26 (dd, J = 14.0, 2.5 Hz, 1H), 3.02 (dd, J = 13.0, 8.5 Hz, 1H), 2.88–2.81 (m, 2H), 2.73 (dd, J = 13.5, 6.0 Hz, 1H), 2.01–1.97 (m, 1H), 0.93 (d, J = 6.5 Hz, 3H), 0.85 (d, J = 6.5 Hz, 3H); 13C NMR (151 MHz, CD3OD) δ 170.2, 160.7, 154.2, 139.9, 134.7,

130.4, 129.2, 127.2, 125.5, 120.1, 118.2, 117.2, 114.4, 74.5, 59.5, 55.3, 54.6, 36.7, 28.1, 20.6, 20.5; HRMS (ESI) m/z calculated for C27H32N3O5S ([M–H]−): 510.2057, found 510.2071.

2.19. N-((2S,3R)-4-((4-amino-N-isobutylphenyl)sulfonamido)-3-hydroxy-1-phenylbutan-2-yl)-4-chloro-2-hydroxybenzamide (16b)

The target compound was obtained by coupling 4-chloro-2-hydroxybenzoic acid (14b) with 4-amino-N-((2R,3S)-3-amino-2-hydroxy-4-phenylbutyl)-N-isobutylbenzenesulfonamide (12) through an EDCI/HOBt/DMAP coupling procedure in 78% yield (white amorphous solid) as described for compound 15a: 1H NMR (500 MHz, CD3OD) δ 7.73 (d, J = 8.0 Hz, 1H), 7.39 (d, J = 8.5 Hz, 2H), 7.35 (t, J = 7.5 Hz, 1H), 7.27 (d, J = 7.5 Hz, 2H), 7.20 (t, J = 7.5 Hz, 2H), 7.12 (t, J = 7.5 Hz, 1H), 6.89–6.85 (m, 2H), 6.56 (d, J = 8.5 Hz, 2H), 4.29 (t, J = 6.5 Hz, 1H), 3.98 (t, J = 6.5 Hz, 1H), 3.38 (d, J = 15.0 Hz, 1H), 3.26 (dd, J = 14.0, 2.5 Hz, 1H), 3.02 (dd, J = 13.0, 9.0 Hz, 1H), 2.90–2.81 (m, 2H), 2.73 (dd, J = 13.0, 6.5 Hz, 1H), 2.00–1.96 (m, 1H), 0.91 (d, J = 6.5 Hz, 3H), 0.84 (d, J = 6.5 Hz, 3H); 13C NMR (126 MHz, CD3OD) δ 170.12, 160.6, 153.6, 139.9, 134.7, 130.4, 130.3, 129.2, 127.2, 125.9, 120.1, 118.2, 117.2, 114.7, 74.4, 59.4, 55.3, 54.5, 36.7, 28.0 20.5; HRMS (ESI) m/z calculated for C27H31ClN3O5S ([M–H]−): 544.1673, found 544.1693.

2.20. N-((2S,3R)-4-((4-amino-N-isobutylphenyl)sulfonamido)-3-hydroxy-1-phenylbutan-2-yl)-2-hydroxy-4-methoxybenzamide (16c)

The target compound was obtained by coupling 2-hydroxy-4-methoxybenzoic acid (14c) with 4-amino-N-((2R,3S)-3-amino-2-hydroxy-4-phenylbutyl)-N-isobutylbenzenesulfonamide (12) through an EDCI/HOBt/DMAP coupling procedure in 82% yield (white amorphous solid) as described for compound 15a: 1H NMR (500 MHz, CD3OD) δ 7.67 (d, J = 9.0 Hz, 1H), 7.39 (d, J = 8.5 Hz, 2H), 7.27 (d, J = 7.5 Hz, 2H), 7.21 (t, J = 7.5 Hz, 2H), 7.13 (t, J = 7.5 Hz, 1H), 6.57 (d, J = 8.5 Hz, 2H), 6.44 (d, J = 9.0 Hz, 1H), 6.41 (s, 1H), 4.26 (dd, J = 11.5, 5.0 Hz, 1H), 3.98 (t, J = 6.5 Hz, 1H),

3.77 (s, 3H), 3.38 (d, J = 14.0 Hz, 1H), 3.26 (dd, J = 14.0, 2.5 Hz, 1H), 3.02 (dd, J = 13.5, 8.5 Hz, 1H), 2.89–2.81 (m, 2H), 2.74 (dd, J = 13.5, 6.5 Hz, 1H), 2.04–1.96 (m, 1H), 0.92 (d, J = 6.5 Hz, 3H), 0.85 (d, J = 6.5 Hz, 3H); 13C NMR (151 MHz, CD3OD) δ 170.4, 165.5, 163.0, 154.1, 139.9, 130.4, 130.3, 129.2, 127.2, 125.5, 114.4, 109.8, 107.3, 102.2, 74.6, 59.4, 55.8, 55.2, 54.6, 36.7, 28.0, 20.5; HRMS (ESI) m/z calculated for C28H34N3O6S ([M–H]−): 540.2163, found 540.2191.

2.21. N-((2S,3R)-4-((4-amino-N-isobutylphenyl)sulfonamido)-3-hydroxy-1-phenylbutan-2-yl)-3,4-dihydroxybenzamide (16d)

The target compound was obtained by coupling 3,4-dihydroxybenzoic acid (14d) with 4-amino-N-((2R,3S)-3-amino-2-hydroxy-4-phenylbutyl)-N-isobutylbenzenesulfonamide (12) through an EDCI/HOBt/DMAP coupling procedure in 70% yield (white amorphous solid) as described for compound 15a: 1H NMR (500 MHz, CD3OD) δ 7.38 (d, J = 8.0 Hz, 2H), 7.27–7.19 (m, 5H), 7.14–7.07 (m, 3H), 6.56 (d, J = 8.0 Hz, 2H), 4.17–4.16 (m, 1H), 3.94 (t, J = 6.5 Hz, 1H), 3.36 (d, J = 13.0 Hz, 1H), 3.27 (d, J = 13.0 Hz, 1H), 3.13–3.12 (m, 1H), 2.88–2.71 (m, 3H), 2.02–1.96 (m, 1H), 0.91 (d, J = 6.5 Hz, 3H), 0.84 (d, J = 6.5 Hz, 3H); 13C NMR (151 MHz, CD3OD) δ 170.1, 156.5, 154.1, 148.5, 140.3, 130.4, 130.3, 129.2, 127.1, 125.6, 120.5, 115.8, 115.7, 114.4, 74.9, 59.4, 55.9, 54.6, 36.8, 28.0, 20.5; HRMS (ESI) m/z calculated for C27H32N3O6S ([M–H]−): 526.2018, found 526.2045.

2.22. N-((2S,3R)-4-((4-amino-N-isobutylphenyl)sulfonamido)-3-hydroxy-1-phenylbutan-2-yl)-2, 4-dihydroxybenzamide (16e)

The target compound was obtained by coupling 2,4-dihydroxybenzoic acid (14e) with 4-amino-N-((2R,3S)-3-amino-2-hydroxy-4-phenylbutyl)-N-isobutylbenzenesulfonamide (12) through an EDCI/HOBt/DMAP coupling procedure in 70% yield (white amorphous solid) as described for compound 15a: 1H NMR (500 MHz, CD3OD) δ 7.62 (d, J = 8.5 Hz, 1H), 7.41 (d, J = 8.5 Hz, 2H), 7.30 (d, J = 7.5 Hz, 2H), 7.25 (t, J = 7.5 Hz, 2H), 7.17 (t, J = 7.0 Hz, 1H), 6.58 (d, J = 8.5 Hz, 2H), 6.35 (dd,

J = 8.5, 2.0 Hz, 1H), 6.30 (d, J = 2.0 Hz, 1H), 4.27–4.23 (m, 1H), 3.98 (t, J = 6.5 Hz, 1H), 3.39 (dd, J = 15.0, 2.0 Hz, 1H), 3.27 (dd, J = 14.0, 3.0 Hz, 1H), 3.04 (dd, J = 13.5, 9.0 Hz, 1H), 2.89–2.82 (m, 2H), 2.75 (dd, J = 13.5, 6.0 Hz, 1H), 2.06–2.00 (m, 1H), 0.96 (d, J = 6.5 Hz, 3H), 0.89 (d, J = 6.5 Hz, 3H); 13C NMR (101 MHz, CD3OD) δ 169.2, 162.4, 161.8, 152.7, 138.6, 129.1, 129.0, 128.9, 127.8, 125.8, 124.1, 107.5, 106.9, 102.5, 73.2, 58.1, 53.7, 53.2, 35.4, 26.6, 19.1; HRMS (ESI) m/z calculated for C27H33N3NaO6S ([M+Na]+): 550.1988, found 550.1983.

2.23. N-((2S,3R)-4-((4-amino-N-isobutylphenyl)sulfonamido)-3-hydroxy-1-phenylbutan-2-yl)-3,5-dihydroxybenzamide (16f)

The target compound was obtained by coupling 3,5-dihydroxybenzoic acid (14f) with 4-amino-N-((2R,3S)-3-amino-2-hydroxy-4-phenylbutyl)-N-isobutylbenzenesulfonamide (12) through an EDCI/HOBt/DMAP coupling procedure in 71% yield (white amorphous solid) as described for compound 15a: 1H NMR (400 MHz, CD3OD) δ 7.38 (d, J = 8.5 Hz, 2H), 7.25–7.18 (m, 4H), 7.11 (t, J = 7.0 Hz, 1H), 6.58 (d, J = 8.5 Hz, 2H), 6.52 (s, 2H), 6.36 (s, 1H), 4.16–4.10 (m, 1H), 3.90 (t, J = 7.0 Hz, 1H), 3.33 (d, J = 15.0 Hz, 1H), 3.28–3.22 (m, 1H), 2.98 (dd, J = 13.5, 8.5 Hz, 1H), 2.87–2.81 (m, 1H), 2.77–2.69 (m, 2H), 1.96 (dt, J = 20.0, 6.5 Hz, 1H), 0.89 (d, J = 6.5 Hz, 3H), 0.83 (d, J = 6.5 Hz, 3H); 13C NMR (101 MHz, CD3OD) δ 169.0, 158.3, 152.8, 138.8, 136.6, 129.0, 128.9, 127.8, 125.8, 124.2, 113.0, 105.3, 105.0, 73.3, 58.0, 54.5, 53.2, 35.4, 26.6, 19.1, 19.0; HRMS (ESI) m/z calculated for C27H33N3NaO6S ([M+Na]+): 550.1988, found 550.1993.

2.24. N-((2S,3R)-4-((4-amino-N-isobutylphenyl)sulfonamido)-3-hydroxy-1-phenylbutan-2-yl)-3,4,5-trihydroxybenzamide (16g)

The target compound was obtained by coupling 3,4,5-trihydroxybenzoic acid (14g) with 4-amino-N-((2R,3S)-3-amino-2-hydroxy-4-phenylbutyl)-N-isobutylbenzenesulfonamide (12) through an EDCI/HOBt/DMAP coupling procedure in 70% yield (white amorphous solid) as described for compound 15a: 1H NMR (500

MHz, CD3OD) δ 7.38 (d, J = 8.0 Hz, 2H), 7.25–7.21 (m, 4H), 7.13–7.12 (m, 1H), 6.72 (s, 2H), 6.57 (d, J = 8.0 Hz, 2H), 4.14 (s, 1H), 3.92 (s, 1H), 3.35 (s, 1H), 3.25 (d, J = 13.5 Hz, 1H), 3.00 (dd, J = 12.0, 9.5 Hz, 1H), 2.86–2.70 (m, 3H), 1.99–1.93 (m, 1H), 0.91 (d, J = 5.5 Hz, 3H), 0.84 (d, J = 5.5 Hz, 3H); 13C NMR (126 MHz, CD3OD) δ 168.9, 152.7, 145.2, 138.9, 129.1, 129.0, 127.9, 125.8, 124.2, 113.1, 106.5, 73.5, 58.1, 54.5, 53.2, 35.5, 26.7, 19.1; HRMS (ESI) m/z calculated for C29H35N3O7S ([M–H]−): 570.2268, found 570.2307.

2.25. N-((2S,3R)-4-((4-amino-N-isobutylphenyl)sulfonamido)-3-hydroxy-1-phenylbutan-2-yl)-4-hydroxy-3,5-dimethoxybenzamide (16h)

The target compound was obtained by coupling 4-hydroxy-3,5-dimethoxybenzoic acid (14h) with 4-amino-N-((2R,3S)-3-amino-2-hydroxy-4-phenylbutyl)-N-isobutylbenzenesulfonamide (12) through an EDCI/HOBt/DMAP coupling procedure in 78% yield (white amorphous solid) as described for 15a: 1H NMR (500 MHz, CD3OD) δ 7.39 (d, J = 8.5 Hz, 2H), 7.27 (d, J = 7.0 Hz, 2H), 7.21 (t, J = 7.5 Hz, 2H), 7.12 (t, J = 7.0 Hz, 1H), 6.99 (s, 2H), 6.54 (d, J = 8.5 Hz, 2H), 4.23–4.19 (m, 1H), 3.99 (t, J = 7.0 Hz, 1H), 3.83 (s, 6H), 3.43 (d, J = 14.0 Hz, 1H), 3.30 (d, J = 7.0 Hz, 1H), 3.03 (dd, J = 13.0, 8.5 Hz, 1H), 2.89–2.79 (m, 2H), 2.74 (dd, J = 13.5, 6.0 Hz, 1H), 2.03–1.98 (m, 1H), 0.92 (d, J = 6.5 Hz, 3H), 0.84 (d, J = 6.5 Hz, 3H); 13C NMR (126 MHz, CD3OD) δ 170.1, 154.1, 148.9, 140.5, 140.3, 130.4, 130.3, 129.2, 127.2, 125.6, 114.3, 106.1, 75.1, 59.5, 56.8, 56.0, 54.7, 36.8, 28.1, 20.6, 20.5; HRMS (ESI) m/z calculated for C29H36N3O7S ([M–H]−): 570.2268, found 570.2307.

2.26. N-((2S,3R)-4-((4-amino-N-isobutylphenyl)sulfonamido)-3-hydroxy-1-phenylbutan-2-yl)-2-hydroxy-4,5-dimethoxybenzamide (16i)

The target compound was obtained by coupling 2-hydroxy-4,5-dimethoxybenzoic acid (14i) with 4-amino-N-((2R,3S)-3-amino-2-hydroxy-4-phenylbutyl)-N-isobutylbenzenesulfonamide (12) through an EDCI/HOBt/DMAP coupling procedure in 83% yield (white amorphous solid) as described for compound 15a: 1H NMR (500

MHz, CD3OD) δ 7.40 (d, J = 8.5 Hz, 2H), 7.33 (s, 1H), 7.31 (d, J = 7.5 Hz, 2H), 7.25 (t, J = 7.5 Hz, 2H), 7.17 (t, J = 7.5 Hz, 1H), 6.57 (d, J = 8.5 Hz, 2H), 6.51 (s, 1H), 4.30–4.25 (m, 1H), 4.00 (td, J = 8.0, 2.5 Hz, 1H), 3.88 (s, 3H), 3.83 (s, 3H), 3.43 (dd, J = 15.0, 2.5 Hz, 1H), 3.29 (dd, J = 14.0, 3.5 Hz, 1H), 3.06 (dd, J = 13.5, 8.5 Hz, 1H), 2.90–2.83 (m, 2H), 2.75 (dd, J = 13.5, 6.0 Hz, 1H), 2.07–2.02 (m, 1H), 0.97 (d, J = 6.5 Hz, 3H), 0.89 (d, J = 6.5 Hz, 3H); 13C NMR (101 MHz, CD3OD) δ 168.8, 155.9, 154.1, 152.8, 141.9, 138.6, 129.0, 127.8, 125.8, 124.0, 112.9, 110.8, 106.5, 100.2, 73.3, 58.1, 56.0, 54.9, 53.9, 53.3, 35.4, 26.6, 19.1; HRMS (ESI) m/z calculated for C29H37N3NaO7S ([M+Na]+): 594.2250, found 594.2267.

2.27. 2-Hydroxy-N-((2S,3R)-3-hydroxy-4-((N-isobutyl-4-(methylthio)phenyl)sulfonamido)-1-phenylbutan-2-yl)benzamide (17a)

The target compound was obtained by coupling 2-hydroxybenzoic acid (14a) with N-((2R,3S)-3-amino-2-hydroxy-4-phenylbutyl)-N-isobutyl-4-(methylthio)benzenesulfonamide (13) through an EDCI/HOBt/DMAP coupling procedure in 88% yield (white amorphous solid) as described for compound 15a: 1H NMR (400 MHz, CD3OD) δ 7.71 (dd, J = 8.0, 1.2 Hz, 1H), 7.52 (d, J = 8.4 Hz, 2H), 7.37–7.32 (m, 1H), 7.24–7.22 (m, 2H), 7.18 (t, J = 7.6 Hz, 2H), 7.11–7.09 (m, 3H), 6.85 (dd, J = 12.0, 4.4 Hz, 2H), 4.23–4.17 (m, 1H), 3.93 (td, J = 8.4, 2.4 Hz, 1H), 3.34 (dd, J = 15.2, 2.4 Hz, 1H), 3.23 (dd, J = 14.0, 3.6 Hz, 1H), 3.09 (dd, J = 13.2, 8.8 Hz, 1H), 2.85 (dd, J = 14.0, 7.2 Hz, 1H), 2.79 (dd, J = 11.6, 8.0 Hz, 1H), 2.74 (dd, J = 12.0, 4.8 Hz, 1H), 2.41 (s, 3H), 2.05–1.94 (m, 1H), 0.91 (d, J = 6.8 Hz, 3H), 0.82 (d, J = 6.8 Hz, 3H); 13C NMR (101 MHz, CD3OD) δ 168.7, 159.4, 145.9, 138.4, 133.9, 133.4, 128.9, 127.9, 127.8, 127.4, 125.8, 124.8, 118.7, 116.9, 115.8, 73.0, 57.8, 53.9, 53.0, 35.4, 26.5, 19.0, 13.2; HRMS (ESI) m/z calculated for C28H34N2NaO5S2 ([M+Na]+): 565.1807, found 565.1841.

2.28. 4-Chloro-2-hydroxy-N-((2S,3R)-3-hydroxy-4-((N-isobutyl-4-(methylthio)phenyl)sulfonamido)-1-phenylbutan-2-yl)benzamide (17b)

The target compound was obtained by coupling 4-chloro-2-hydroxybenzoic acid (14b) with N-((2R,3S)-3-amino-2-hydroxy-4-phenylbutyl)-N-isobutyl-4-(methylthio)benzenesulfonamide (13) through an EDCI/HOBt/DMAP coupling procedure in 85% yield (white amorphous solid) as described for compound 15a: 1H NMR (400 MHz, CD3OD) δ 7.65 (d, J = 8.4 Hz, 1H), 7.55 (d, J = 8.4 Hz, 2H), 7.25–7.23 (m, 2H), 7.18 (dd, J = 10.0, 4.8 Hz, 2H), 7.12–7.07 (m, 3H), 6.81 (d, J = 2.0 Hz, 1H), 6.68 (dd, J = 8.4, 2.0 Hz, 1H), 4.22–4.16 (m, 1H), 3.95–3.91 (m, 1H), 3.32 (dd, J = 15.2, 2.4 Hz, 1H), 3.20 (dd, J = 14.0, 4.0 Hz, 1H), 3.08 (dd, J = 13.2, 8.8 Hz, 1H), 2.87 (dd, J = 15.2, 8.8 Hz, 1H), 2.79–2.71 (m, 2H), 2.42 (s, 3H), 2.02–1.94 (m, 1H), 0.90 (d, J = 6.8 Hz, 3H), 0.82 (d, J = 6.8 Hz, 3H); 13C NMR (101 MHz, CD3OD) δ 168.3, 163.7, 145.8, 138.5, 138.2, 133.9, 129.8, 129.0, 127.8, 127.5, 125.8, 124.8, 118.2, 116.6, 115.6, 72.9, 57.7, 54.1, 52.9, 35.7, 26.5, 19.0, 13.2; HRMS (ESI) m/z calculated for C28H33ClN2NaO5S2 ([M+Na]+): 599.1417, found 599.1436.

2.29. 2-Hydroxy-N-((2S,3R)-3-hydroxy-4-((N-isobutyl-4-(methylthio)phenyl)sulfonamido)-1-phenylbutan-2-yl)-4-methoxybenzamide (17c)

The target compound was obtained by coupling 2-hydroxy-4-methoxybenzoic acid (14c) with N-((2R,3S)-3-amino-2-hydroxy-4-phenylbutyl)-N-isobutyl-4-(methylthio)benzenesulfonamide (13) through an EDCI/HOBt/DMAP coupling procedure in 85% yield (white amorphous solid) as described for compound 15a: 1H NMR (400 MHz, CD3OD) δ 7.64 (d, J = 9.0 Hz, 1H), 7.52–7.50 (m, 2H), 7.23–7.21 (m, 2H), 7.17 (dd, J = 10.0, 4.8 Hz, 2H), 7.11–7.07 (m, 3H), 6.43 (dd, J = 9.0, 2.4 Hz, 1H), 6.38 (d, J = 2.4 Hz, 1H), 4.19–4.13 (m, 1H), 3.93 (td, J = 8.4, 2.4 Hz, 1H), 3.76 (s, 3H), 3.33–3.29 (m, 1H), 3.22 (dd, J = 14.0, 3.6 Hz, 1H), 3.09 (dd, J = 13.2, 8.8 Hz, 1H), 2.85–2.77 (m, 2H), 2.75–2.70 (m, 1H), 2.41 (s, 3H), 2.04–1.94 (m, 1H), 0.91 (d, J = 6.8 Hz, 3H), 0.82 (d, J = 6.8 Hz, 3H); 13C NMR (101 MHz, CD3OD) δ 168.9, 164.2, 161.9, 145.9, 138.5, 133.8, 129.0, 128.9, 127.8, 127.4, 125.8, 124.8, 108.5, 105.9, 100.9, 73.1, 57.9, 54.5, 53.8, 53.1, 35.5, 26.5, 19.0, 13.2; HRMS (ESI) m/z calculated for C29H36N2NaO6S2 ([M+Na]+): 595.1912, found 595.1947.

2.30. 3,4-Dihydroxy-N-((2S,3R)-3-hydroxy-4-((N-isobutyl-4-(methylthio)phenyl)sulfonamido)-1-phenylbutan-2-yl)benzamide (17d)

The target compound was obtained by coupling 3,4-dihydroxybenzoic acid (14d) with N-((2R,3S)-3-amino-2-hydroxy-4-phenylbutyl)-N-isobutyl-4-(methylthio)benzenesulfonamide (13) through an EDCI/HOBt/DMAP coupling procedure in 78% yield (white amorphous solid) as described for compound 15a: 1H NMR (400 MHz, CD3OD) δ 7.54 (d, J = 8.4 Hz, 2H), 7.24–7.22 (m, 2H), 7.20–7.18 (m, 2H), 7.16–7.13 (m, 3H), 7.09 (t, J = 7.2 Hz, 2H), 6.76 (d, J = 8.0 Hz, 1H), 4.11 (ddd, J = 11.2, 8.0, 3.6 Hz, 1H), 3.93 (td, J = 8.8, 2.4 Hz, 1H), 3.33 (dd, J = 15.2, 2.4 Hz, 1H), 3.27–3.23 (m, 1H), 3.08 (dd, J = 13.6, 8.8 Hz, 1H), 2.85 (dd, J = 15.2, 8.8 Hz, 1H), 2.78–2.72 (m, 2H), 2.42 (s, 3H), 2.05–1.95 (m, 1H), 0.91 (d, J = 6.8 Hz, 3H), 0.82 (d, J = 6.8 Hz, 3H); 13C NMR (101 MHz, CD3OD) δ 168.5, 148.8, 145.9, 144.9, 138.9, 133.9, 128.9, 127.8, 127.4, 125.7, 125.5, 124.8, 119.1, 114.4, 114.3, 73.4, 57.8, 54.5, 53.1, 35.6, 26.5, 19.0, 13.2; HRMS (ESI) m/z calculated for C28H34N2NaO6S2 ([M+Na]+): 581.1756, found 581.1767.

2.31. 2, 4-Dihydroxy-N-((2S,3R)-3-hydroxy-4-((N-isobutyl-4-(methylthio)phenyl)sulfonamido)-1-phenylbutan-2-yl)benzamide (17e)

The target compound was obtained by coupling 2,4-dihydroxybenzoic acid (14e) with N-((2R,3S)-3-amino-2-hydroxy-4-phenylbutyl)-N-isobutyl-4-(methylthio)benzenesulfonamide (13) through an EDCI/HOBt/DMAP coupling procedure in 72% yield (white amorphous solid) as described for compound 15a: 1H NMR (500 MHz, CD3OD) δ 7.65 (d, J = 8.5 Hz, 1H), 7.57 (d, J = 8.5 Hz, 2H), 7.29 (d, J = 7.5 Hz, 2H), 7.26–7.23 (m, 2H), 7.18–7.15 (m, 3H), 6.38 (dd, J = 8.5, 2.0 Hz, 1H), 6.33 (d, J = 2.0 Hz, 1H), 4.23–4.18 (m, 1H), 4.00 (t, J = 7.5 Hz, 1H), 3.38–3.35 (m, 1H), 3.29 (dd, J = 14.0, 3.0 Hz, 1H), 3.16 (dd, J = 14.0, 9.0 Hz, 1H), 2.88–2.82 (m, 2H), 2.78 (dd, J = 13.5, 6.0 Hz, 1H), 2.48 (s, 3H), 2.10–2.03 (m, 1H), 0.99 (d, J = 6.5 Hz, 3H), 0.90 (d, J = 6.5 Hz, 3H); 13C NMR (101 MHz, CD3OD) δ 169.1, 162.5, 161.8, 145.9,

138.6, 133.6, 129.2, 128.9, 127.8, 127.4, 125.8, 124.8, 107.5, 107.0, 102.5, 73.2, 58.0, 53.7, 53.2, 35.6, 26.5, 19.0, 13.2; HRMS (ESI) m/z calculated for C28H34N2NaO6S2 ([M+Na]+): 581.1756, found 581.1779.

2.32. 3,5-Dihydroxy-N-((2S,3R)-3-hydroxy-4-((N-isobutyl-4-(methylthio)phenyl)sulfonamido)-1-phenylbutan-2-yl)benzamide (17f)

The target compound was obtained by coupling 3,5-dihydroxybenzoic acid (14f) with N-((2R,3S)-3-amino-2-hydroxy-4-phenylbutyl)-N-isobutyl-4-(methylthio)benzenesulfonamide (13) through an EDCI/HOBt/DMAP coupling procedure in 74% yield (white amorphous solid) as described for compound 15a: 1H NMR (500 MHz, CD3OD) δ 7.63 (d, J = 8.5 Hz, 2H), 7.31–7.26 (m, 6H), 7.18 (t, J = 7.0 Hz, 1H), 6.62 (d, J = 2.0 Hz, 2H), 6.45 (s, 1H), 4.18–4.14 (m, 1H), 3.99 (t, J = 7.5 Hz, 1H), 3.41 (d, J = 13.5 Hz, 1H), 3.31 (d, J = 3.0 Hz, 1H), 3.16 (dd, J = 13.5, 9.0 Hz, 1H), 2.92 (dd, J = 15.0, 9.0 Hz, 1H), 2.84–2.78 (m, 2H), 2.52 (s, 3H), 2.12–2.04 (m, 1H), 0.99 (d, J = 6.5 Hz, 3H), 0.90 (d, J = 6.5 Hz, 3H); 13C NMR (101 MHz, CD3OD) δ 168.9, 158.4, 146.0, 138.8, 136.4, 134.0, 128.9, 127.8, 127.4, 125.8, 124.9, 105.3, 105.1, 73.3, 57.8, 54.6, 53.0, 35.5, 26.5, 19.0, 13.1; HRMS (ESI) m/z calculated for C28H34N2NaO6S2 ([M+Na]+): 581.1756, found 581.1765.

2.33. 4-Hydroxy-N-((2S,3R)-3-hydroxy-4-((N-isobutyl-4-(methylthio)phenyl)sulfonamido)-1-phenylbutan-2-yl)-3,5-dimethoxybenzamide (17h)

The target compound was obtained by coupling 4-hydroxy-3,5-dimethoxybenzoic acid (14h) with N-((2R,3S)-3-amino-2-hydroxy-4-phenylbutyl)-N-isobutyl-4-(methylthio)benzenesulfonamide (13) through an EDCI/HOBt/DMAP coupling procedure in 83% yield (white amorphous solid) as described for compound 15a: 1H NMR (500 MHz, DMSO-d6) δ 8.14 (d, J = 9.0 Hz, 1H), 7.61 (d, J = 8.5 Hz, 2H), 7.29 (d, J = 7.5 Hz, 2H), 7.25–7.21 (m, 4H), 7.14 (d, J = 7.0 Hz, 1H), 7.11 (s, 2H), 4.06 (q, J = 8.0 Hz, 1H), 3.83 (s, 7H), 3.32 (s, 1H), 3.20 (d, J = 12.0 Hz, 1H), 3.09 (dd, J = 13.5, 9.0 Hz, 1H), 2.85–2.78 (m, 2H), 2.74 (dd, J = 14.0, 6.0 Hz, 1H), 2.49 (s, 3H), 2.04–1.99

(m, 1H), 0.90 (d, J = 6.5 Hz, 3H), 0.82 (d, J = 6.5 Hz, 3H); 13C NMR (101 MHz, DMSO-d6) δ 166.1, 147.8, 145.2, 140.3, 139.0, 134.6, 129.6, 128.4, 128.0, 126.2, 125.5, 124.6, 105.7, 73.3, 57.4, 56.6, 54.7, 53.2, 35.6, 26.5, 20.4, 14.2; HRMS (ESI) m/z calculated for C30H38N2NaO7S2 ([M+Na]+): 625.2018, found 625.2012.

2.34. 2-Hydroxy-N-((2S,3R)-3-hydroxy-4-((N-isobutyl-4-(methylthio)phenyl)sulfonamido)-1-phenylbutan-2-yl)-4,5-dimethoxybenzamide (17i)

The target compound was obtained by coupling 2-hydroxy-4,5-dimethoxybenzoic acid (14i) with N-((2R,3S)-3-amino-2-hydroxy-4-phenylbutyl)-N-isobutyl-4-(methylthio)benzenesulfonamide (13) through an EDCI/HOBt/DMAP coupling procedure in 85% yield (white amorphous solid) as described for compound 15a: 1H NMR (500 MHz, CD3OD) δ 7.58 (d, J = 8.5 Hz, 2H), 7.34 (s, 1H), 7.30 (d, J = 7.0 Hz, 2H), 7.27–7.24 (m, 2H), 7.19–7.15 (m, 3H), 6.54 (s, 1H), 4.25–4.21 (m, 1H), 4.01 (t, J = 7.5 Hz, 1H), 3.89 (s, 3H), 3.84 (s, 3H), 3.40 (d, J = 14.0 Hz, 1H), 3.30 (dd, J = 14.0, 3.0 Hz, 1H), 3.18 (dd, J = 13.5, 9.0 Hz, 1H), 2.89–2.85 (m, 2H), 2.79 (dd, J = 13.5, 6.0 Hz, 1H), 2.48 (s, 3H), 2.12–2.04 (m, 1H), 0.99 (d, J = 6.5 Hz, 3H), 0.90 (d, J = 6.5 Hz, 3H); 13C NMR (101 MHz, CD3OD) δ 168.7, 155.6, 154.2, 145.9, 142.1, 138.5, 133.8, 128.9, 127.8, 127.4, 125.8, 124.8, 110.9, 106.5, 100.2, 73.3, 57.9, 56.0, 55.0, 53.9, 53.2, 35.5, 26.5, 19.0, 13.1; HRMS (ESI) m/z calculated for C30H38N2NaO7S2 ([M+Na]+): 625.2018, found 625.2004.
